# A scalable and green one-minute synthesis of substituted phenols†

**DOI:** 10.1039/d0ra08580d

**Published:** 2020-11-07

**Authors:** Vijayaragavan Elumalai, Jørn H. Hansen

**Affiliations:** UiT The Arctic University of Norway, Department of Chemistry, Chemical Synthesis and Analysis Group N9037 Tromsø Norway jorn.h.hansen@uit.no

## Abstract

A mild, green and highly efficient protocol was developed for the synthesis of substituted phenols *via ipso*-hydroxylation of arylboronic acids in ethanol. The method utilizes the combination of aqueous hydrogen peroxide as the oxidant and H_2_O_2_/HBr as the reagent under unprecedentedly simple and convenient conditions. A wide range of arylboronic acids were smoothly transformed into substituted phenols in very good to excellent yields without chromatographic purification. The reaction is scalable up to at least 5 grams at room temperature with one-minute reaction time and can be combined in a one-pot sequence with bromination and Pd-catalyzed cross-coupling to generate more diverse, highly substituted phenols.

## Abstract 2

The synthesis of highly substituted phenols is advanced *via* a green one minute hydroxylation and subsequent one-pot functionalizations.

## Graphical abstract

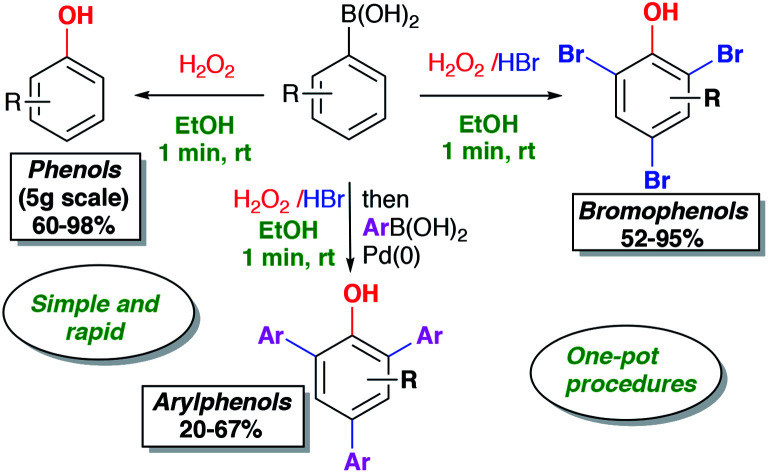

## Introduction

**Scheme 1:**
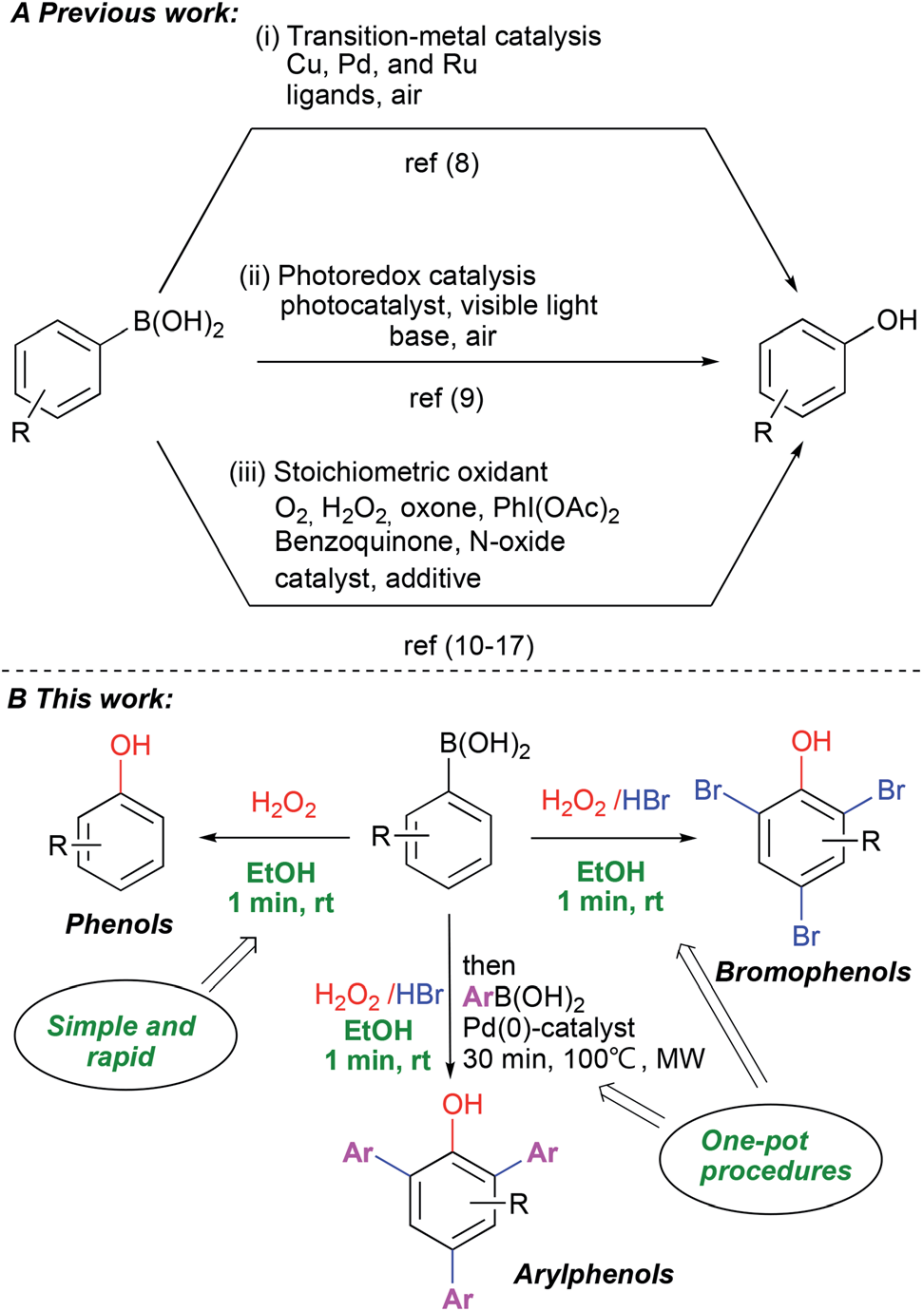
*Ipso*-hydroxylation of phenylboronic acids.

Phenols are indispensable in synthesis and constitute a privileged structural scaffold present in pharmaceuticals, natural products and synthetic polymers, among many other applications.^1^ Consequently, considerable attention has been focused on the synthesis of phenol and its derivatives.^2^ Traditional methods include nucleophilic aromatic substitution of aryl halides,^3^ transition-metal catalyzed processes,^4^ hydroxylation of benzene,^5^ oxidation of cumene^6^ and hydrolysis of diazo compounds.^7^ These methods have some major limitations, including harsh reaction conditions, reagent toxicities and the need for additives and complex ligands. The *ipso*-hydroxylation of boronic acids is an alternative route for the synthesis of phenols which avoids many of the problems with previous approaches. The boronic acids have low toxicity, higher stability in air, are easy to handle and display high functional group diversity. Aryl and heteroarylboronic acids are readily available and have been employed in several studies on *ipso*-hydroxylation chemistry. These mainly involved transition metal catalysis^8^ (Scheme 1A-i), photocatalysis^9^ (Scheme 1A-ii) or stoichiometric oxidants such as H_2_O_2_,^10^ oxone,^11^*N*-oxides,^12^ organic hypervalent iodine(iii),^13^ benzoquinone,^14^ mCPBA,^15^ NaBO_3_ (ref. 16) and molecular oxygen^17^ (Scheme 1A-iii). Moreover, numerous variants have been reported recently for the synthesis of substituted phenols from boronic acids.^18^ Despite the high efficiency of many of these protocols, they are often characterized by high temperatures and long reaction times, the use of transition metals, necessity of strong base addition and the use of harmful solvents. Furthermore, in many of these methods, scaled-up synthesis of phenols has not been demonstrated. When summarizing published papers on the use of hydrogen peroxide,^18*g*^ as one of the simplest and most available green oxidants, surprisingly, we find that virtually all of them report using extra additives of some kind and relatively long reaction times.

Recognizing the power of the transformation in the synthesis of diverse phenols, we set out to find mild, practical and simple reaction conditions with a minimum of additives, high chemical yields and with a clear green profile. Herein we report a very rapid, catalyst/additive-free and practical protocol for the synthesis of phenols *via* the *ipso*-hydroxylation of arylboronic acids using hydrogen peroxide in ethanol. The protocol represents a significant advance in the field as it works with unprecedented efficiency under ambient, green conditions and affords substantially improved chemical yields of phenols with only one minute reaction time and without flash column chromatography. Furthermore, adding hydrogen bromide yields bromophenols directly in a completely novel one-pot system which advances a truly practical and scalable approach to access highly substituted phenols (Scheme 1B).

## Results and discussion

Previous reports on the use of hydrogen peroxide as oxidant for the *ipso*-hydroxylation were found to be of high interest because of the simplicity and clear green profile of H_2_O_2_.^10,18*g*^ Although several solvent systems have been reported with hydrogen peroxide, we decided to screen a range of solvents to ascertain reaction performance at ambient temperature and atmosphere with only one-minute reaction time. These reaction conditions underline our search for a highly efficient and truly practical transformation. In the solvent survey, conducted with the reaction between phenylboronic acid 1a and hydrogen peroxide to generate phenol 2a (Table 1), we were pleased to observe that medium to excellent yields were obtainable across the board. A range of commonly employed solvents such as methanol, THF, acetone, ethyl acetate and acetonitrile afforded high yields (82–89%), whereas water and DCM gave medium yields (50–55%). The major issue appears to be poor solubility of the boronic acid in the latter. The highest isolated yield of 94% was obtained in ethanol, a readily accessible, convenient and green solvent. Therefore, ethanol was the solvent of choice for further studies. The most striking result from our studies is that the reaction time of only one minute in ethanol provides excellent yields at ambient temperature in an open flask. Our observations and simple conditions have not been reported in previous works to the best of our knowledge.

**Table 1:** Survey of solvents

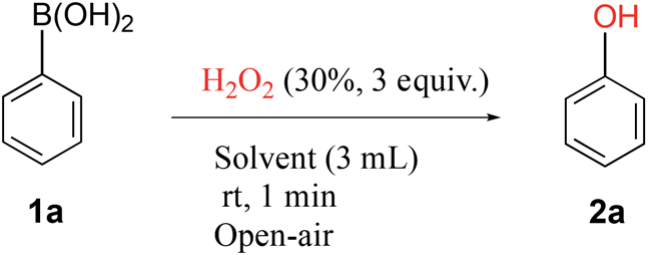		
Entry^a^	Solvent	Yield 2a^b^ (%)
1	MeOH	87
**2**	**EtOH**	**92**
3	THF	82
4	EtOAc	89
5	H_2_O	55^c^^,^^d^
6	Acetone	84
7	MeCN	86
8	DCM	50^c^^,^^d^

a Reaction procedure: to a stirred solution of phenylboronic acid 1a (1.0 mmol) in EtOH (3 mL) was added H_2_O_2_ (30%, 3 equiv.) and stirred for one minute at room temperature.

b Isolated yield.

c NMR-yield.

d Poor solubility of 1a.

The effect of oxidant equivalency was briefly investigated next (Table 2). Using one equivalent of peroxide yielded 50% (NMR) of the product after a set one minute reaction time. Increasing to two equivalents gave full conversion to product in 99% NMR-yield after one minute. Prolonging the reaction time to 5 minutes did not induce any observable changes. Also, further increasing the oxidant equivalency to 3 retained 99% NMR-yield of the desired product. In further scope studies we decided to employ 3 equivalents of hydrogen peroxide in order to ensure full conversion at one minute reaction time. However, it should be noted that near equimolar amounts of oxidant may be sufficient with somewhat prolonged reaction times.

**Table 2:** Effect of H_2_O_2_ ratio with reaction time on overall product yield

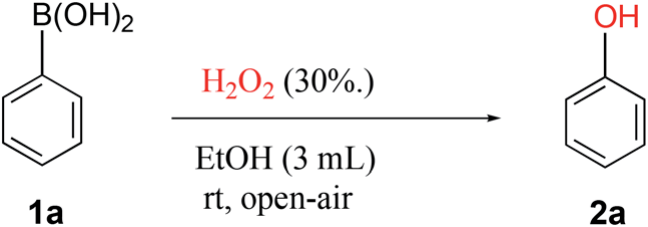			
Entry^a^	H_2_O_2_ (equiv.)	Time (min)	Yield 2a^b^(%)
1	1	1	50
2	2	1	99
3	2	5	99
**4**	**3**	**1**	**99**

a Reaction procedure: to a stirred solution of phenylboronic acid 1 (1.0 mmol) in EtOH (3 mL) was added H_2_O_2_ (30%) and stirred for one minute at room temperature.

b NMR-yield.

**Scheme 2:**
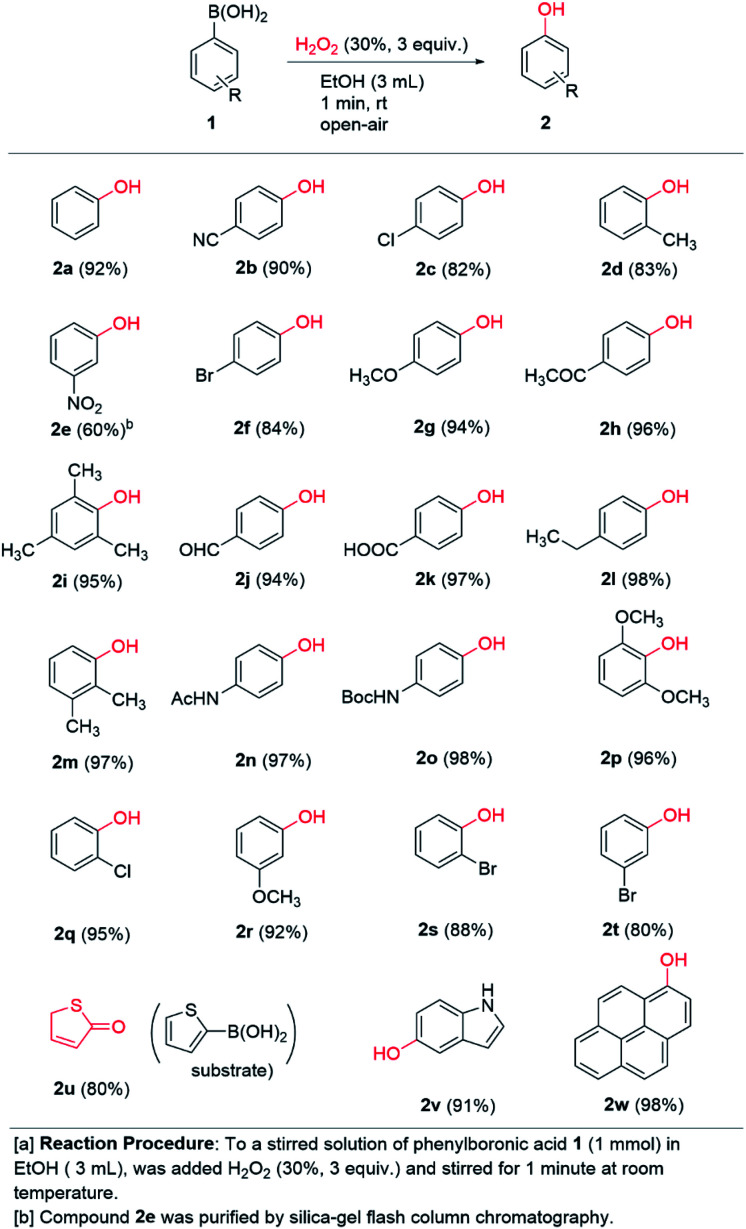
Scope of *ipso*-hydroxylation chemistry.

A range of commercially available boronic acids were surveyed as substrates for the chemistry in order to ascertain the generality of our conditions. To our delight, good to excellent chemical yields (60–98%) were obtained across the board in 23 examples detailed in Scheme 2. Notably, most of the examples were isolated by simple extraction in pure form and further purification was not necessary. A range of *p*-substituted phenylboronic acids containing electron donating (methoxy, ethyl, acetamido, Boc-amino) and electron withdrawing groups (chloro, bromo, cyano, acetyl, carboxaldehyde, carboxylic acid) afforded 94–98% and 82–97% yields, respectively. Thus, electronic diversity in the substituents are well tolerated in the transformation. Notably, 2n is the commercial pharmaceutical paracetamol and was formed in 97% isolated yield from our one-minute protocol. Even *ortho*-substituted boronic acids work well in high to excellent chemical yields (83–96%). Notably, 2i and 2p were formed in excellent 95% and 96% yields, respectively, thus demonstrating very high tolerance to steric hindrance around the boronic acid moiety. Likewise, *meta*-substituted substrates were well tolerated across electronic diversity (80–92%), except of the *meta*-nitro substituted boronic acid 1e, which afforded 60% yield of 2e. This was the only entry that required flash column chromatography to obtain pure material. Furthermore, the chemistry is compatible with heterocycles as demonstrated with thiophene-2-boronic acid 1u and indole-5-boronic acid 1v. The former was isolated as the keto-form 2u, which is a more stable tautomer of the corresponding aromatic hydroxy compound, in 80% yield. The 5-hydroxyindole product 2v was isolated in 91% yield. To our delight, the polyaromatic pyrene boronic acid 1w underwent a highly efficient transformation to 2w in 98% yield. Thus, in addition to very short reaction time and practical conditions, the chemistry appears to be relatively independent of the electronic and steric nature of the substrate. The transformation under our conditions clearly has a broad scope and potential great synthetic utility in the formation of aromatic hydroxy compounds.

**Scheme 3:**
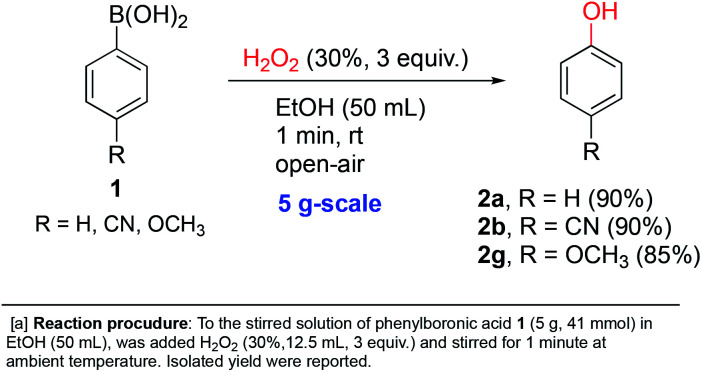
Gram-scale synthesis of phenols.

In order to further demonstrate the applicability of our reaction conditions, three of the *ipso*-hydroxylations were conducted at 5 gram-scale by simple scale-up of *p*-substituted systems. 85–90% isolated yields were obtained with electron-donating (methoxy), electron-withdrawing (cyano) and electron-neutral (H) examples shown in Scheme 3 (2a, 2b and 2g). This suggests that our conditions should be the method of choice for such a transformation conducted at larger scales for synthetic purposes.

**Scheme 4:**
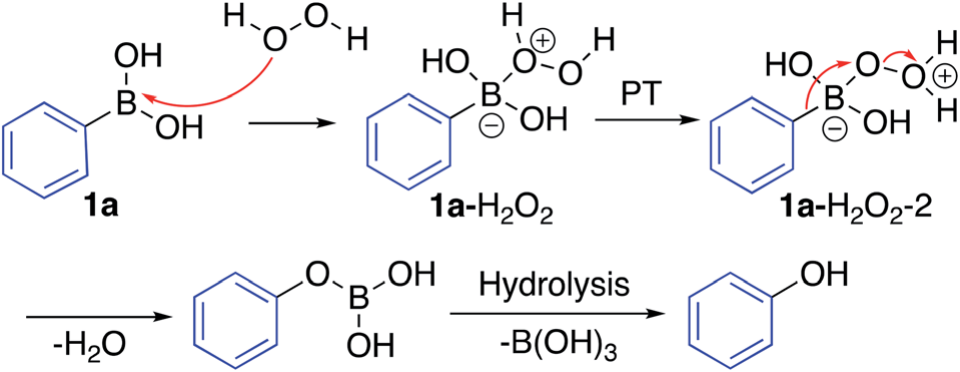
Hypothesized reaction mechanism for the *ipso*-hydroxylation of boronic acids.

The mechanism for the *ipso*-hydroxylation of boronic acids is presumed to be as outlined in Scheme 4.^19^ The boronic acid can form an adduct with hydrogen peroxide which can undergo rapid proton transfer (particularly in protic solvents) to afford intermediate 1a–H_2_O_2_–2, set up for 1,2-aryl migration with departure of water, which gives the boronic ester readily amenable to hydrolysis to afford the phenol.

**Scheme 5:**
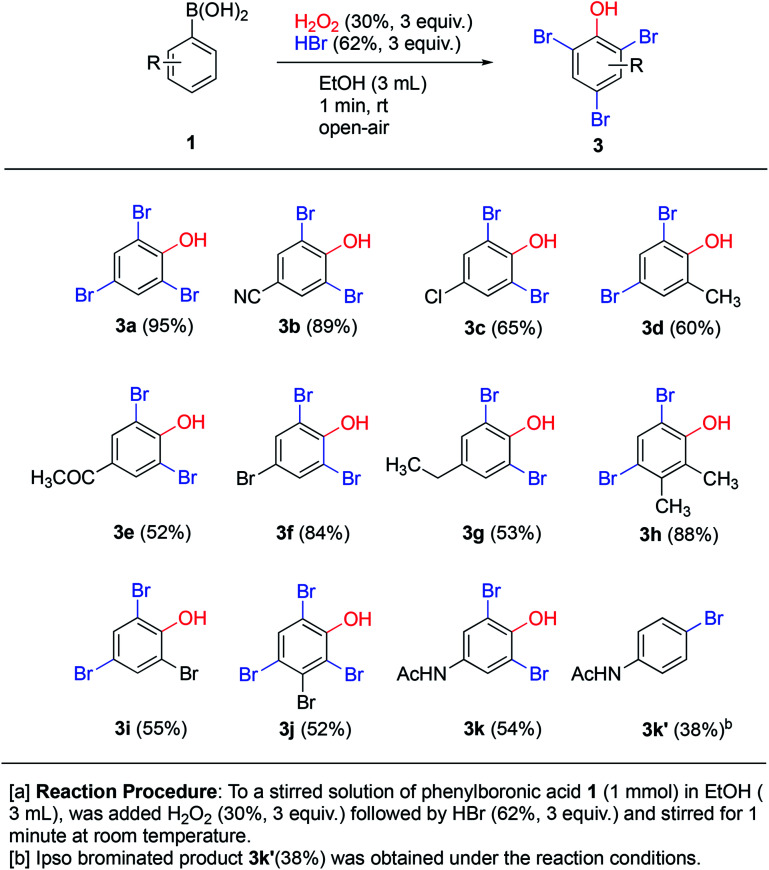
Tandem *ipso*-hydroxylation-bromination.

Treatment of phenols with HBr under oxidative conditions is known to yield bromophenols *via* electrophilic aromatic substitution.^10*f*^ To our delight, the addition of hydrogen bromide to the *ipso*-hydroxylation reaction from the very start yielded brominated phenols 3a–k directly (Scheme 5), even though the reaction time was still kept at one minute. This direct one-pot protocol for generation of bromophenols from arylboronic acids is novel. The reaction likely proceeds by tandem *ipso*-hydroxylation-bromination, both processes which are facilitated by hydrogen peroxide. All the reactions involved must be rapid, since only a minute total reaction time afforded medium to excellent yields of the bromophenols (53–95% yield). The simple phenylboronic acid 1a afforded the tribrominated phenol 3a in excellent 95% yield. The electron-withdrawing *p*-cyano system 1b gave only slightly diminished yield (89%) of 3b. The remaining *p*-substituted systems afforded medium to good yields of *o*-dibrominated products in 52–84% yields. *Ortho*- and *meta*-substituted systems afforded *ortho*,*para*-dibrominated products in 55–88% yields. The *meta*-brominated substrate 1j afforded the tetrabromophenol 3j in 52% yield. The *para*-acetamidoboronic acid 1k afforded the *ortho*-dibrominated phenol 3k in 54% yield, which represents a formal synthesis of bromo-functionalized pharmaceutical agent paracetamol. In this case, another product 3k′ was also isolated which turned out to be *para*-bromoacetamidobenzene in 38% yield. This represents a formal *ipso*-bromination of the boronic acid.^20^ The tandem *ipso*-hydroxylation – bromination procedure appears to work quite well with a range of substrate and affords medium to excellent yields of bromophenols. This represents an attractive and direct route to this class of compounds due to its high efficiency and convenient procedure.

**Scheme 6:**
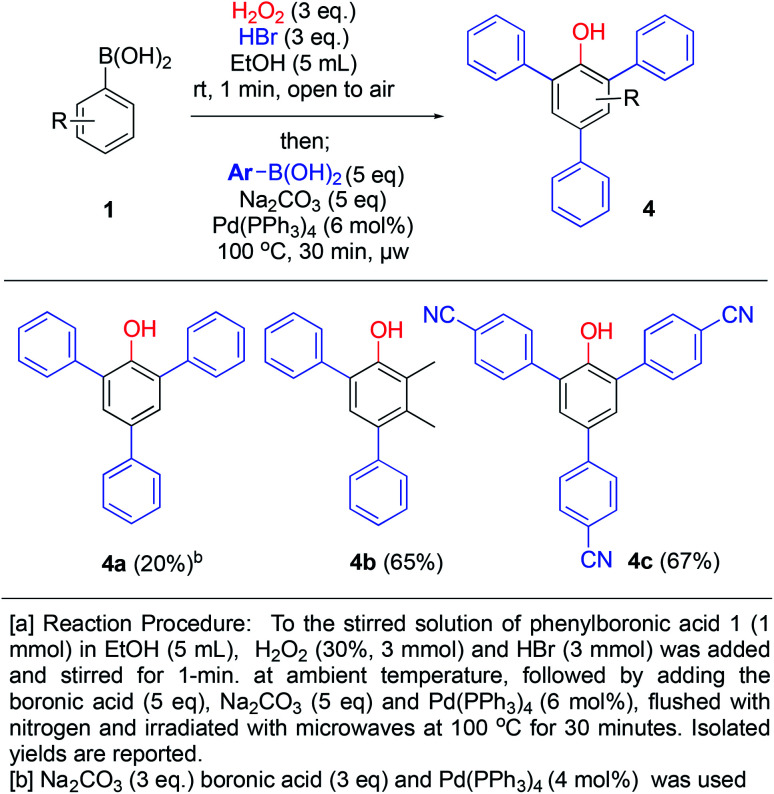
One-pot *ipso*-hydroxylation-bromination and Suzuki coupling to generate aryl-substituted phenols.

Our development of the one minute route to bromophenols, triggered us to investigate whether another reaction could be combined in the same pot in order to access further functionalized phenols. The presence of bromo-substituents would allow access to arylated phenols *via* cross-coupling chemistry. By generating bromophenols as described above, followed by addition of base, 5 equivalents of a new boronic acid, tetrakispalladiumtriphenylphosphine (6 mol%) and degassing with nitrogen, the Suzuki–Miyaura coupling products 4a–c were generated in 20–67% yields (over 3 steps) upon microwave heating for 30 min (Scheme 6). 4a was formed in diminished 20% yield using 3 equivalents of boronic acid and base with 4 mol% of Pd-catalyst loading. However, by increasing to 5 equivalents of boronic acid and base with 6 mol% of catalyst loading (2 mol% per coupling), the yields increased to 65% and 67% for 4b and 4c, respectively. Over 3 steps this is close to 90% average yield per step (unoptimized), thus, it constitutes an impressive one-pot procedure for generating such highly substituted phenols. Furthermore, 4b is not symmetrical and demonstrates that this methodology can be applied to generate relatively complex phenols.

## Conclusions

In summary, we have developed a very rapid and green synthesis of phenols from boronic acids *via ipso*-hydroxylation mediated by hydrogen peroxide in ethanol at ambient conditions. The method appears quite general and affords very good to excellent yields across a range of sterically and electronically diverse substituent patterns. By addition of hydrogen bromide to the reaction mixture, diverse bromophenols are available through a tandem hydroxylation/bromination process. The chemistry has been applied to develop a synthesis of highly substituted arylphenols *via* a one-pot *ipso*-hydroxylation/bromination/Suzuki–Miyaura coupling sequence. The results should be of broad interest to the chemical community and represent major advances in the synthesis of complex phenols.

## Conflicts of interest

There are no conflicts to declare.

## Supplementary Material

RA-010-D0RA08580D-s001
