# Supplementary material for: A scalable and green one-minute synthesis of substituted phenols
Source: RSC Adv. 2020 Nov 7;10(66):40582–7. doi: 10.1039/d0ra08580d (PMC9057563; doi:10.1039/d0ra08580d)

Electronic Supplementary Material (ESI) for RSC Advances.
This journal is © The Royal Society of Chemistry 2020

A scalable and green one-minute synthesis of substituted phenols

Vijayaragavan Elumalai and Jørn H. Hansen*

UiT The Arctic University of Norway, Department of Chemistry, Chemical Synthesis and Analysis Group, N9037 Tromsø, Norway

*Corresponding author email: jorn.h.hansen@uit.no

Table of contents:

[I] General methods S2

[II] Experimental Section S2

(a) General Procedure for the synthesis phenols (**open-air**) S2

(b) General Procedure for the synthesis of bromophenols (one-pot ipso-hydroxylation/bromination) (**open-air**) .S2

(c) General Procedure for the synthesis of phenols (**Scale-up**) S3

(e) Phenol derivatives **[2a-w]**..S4

(f) Bromophenol derivatives **[3a-k']**..S8

(g) Aryl substituted Phenols (one-pot ipso-hydroxylation/bromination/Suzuki coupling) **[4a-4c]**..........S11

[III] ^{1}H & ^{13}C-NMR , GC-MS spectra.. S13

[I] General Methods

All reagents and solvents were purchased commercially and used directly without any further purification. ^{1}H and ^{13}C-NMR spectra were recorded at ambient temperature at a frequency of 400 and 101 MHz, respectively. Flash-column chromatography was performed by using the indicated solvent system and silicagel (40–63 mm). TLC was performed on Merck silica gel 60 F254 plates, using UV light at 254 nm for a detection. GC-MS chromatograms were recorded on a Thermo Scientific Trace GC Ultra with a Thermo Scientific ITQ 1100 detector. The chemical shifts are reported in ppm relative to residual $CDCl_3$ for proton (δ = 7.26 ppm) and $CDCl_3$ for carbon (δ = 77.0 ppm) and with DMSO-d_6 for proton (δ = 2.50 ppm) and for carbon (δ = 39.0 ppm) and CD_3OD for proton (δ = 3.31 ppm) and for carbon (δ = 41.0 ppm) with tetramethylsilane as an external reference. The splitting patterns were recorded as a singlet (s), doublet (d), triplet (t), quartet (q), doublet of doublet (dd), doublet of triplet (dt), triplet of doublets (td), doublet of doublet of doublets (ddd), multiplet (m). High-resolution mass spectra (HRMS) were recorded using MeOH solution on LTQ Orbitrap XL in a positive or negative electrospray ionization (ESI) method.

[II] Experimental Section

(a) General Procedure for the synthesis of aryl phenols (open-air)

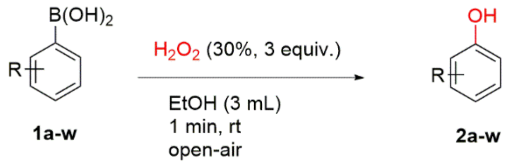

In a RB-flask (25 mL), arylphenylboronic acid **1a-w** (1 mmol) was dissolved in EtOH (3 mL). To the stirred solution, H_2O_2 (30%, 3 mmol) was added and stirred for 1 minute at ambient temperature. After the reaction time, the reaction was quenched with water (10 mL), extracted with ethyl acetate (50 mL) and washed with water (40 mL). The water layer was extracted with ethyl acetate (2 × 30 mL). The organic layers were combined and dried over anhydrous Na_2SO_4. The drying agent was removed by filtration and the solvent was evaporated under reduced pressure to obtain the desired phenol compound **2a-d, 2f-w** without further purification.

(b) General Procedure for the synthesis of aryl bromophenols (open-air)

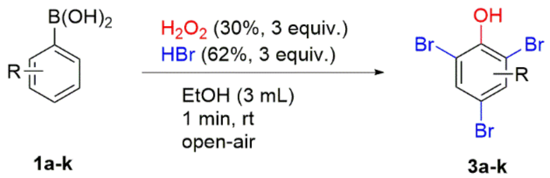

In a RB-flask (25 mL), arylphenylboronic acid **1a-k** (1 mmol) was dissolved in EtOH (3 mL). To the stirred solution, H_2O_2 (30%, 3 mmol) followed by HBr (62%, 3 mmol) was added and stirred for 1 minute at ambient temperature. After the reaction time, the reaction was quenched with water (10 mL), extracted with ethyl acetate (50 mL) and washed with water (40 mL). The water layer was extracted with ethyl acetate (2 × 30 mL). The organic layers were combined and dried over anhydrous Na_2SO_4. The drying agent was removed by filtration and the solvent was evaporated under reduced pressure to obtain the crude product and further purified by silica-gel flash column chromatography using the ethyl acetate/pentane to obtain the desired phenol compounds **3a-k.**

(c) General Procedure for the synthesis of aryl phenols (Scale-up)

Example: 1

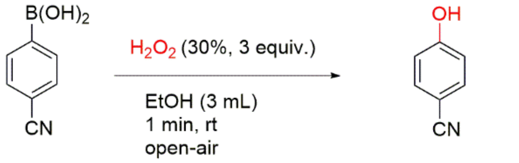

1b **2b (90%)**

In a RB-flask (25 mL), arylphenylboronic acid **1b** (5 g, 34 mmol) was dissolved in EtOH (3 mL). To the stirred solution, H_2O_2 (30%, 10.4 mL, 102 mmol) was added and stirred for 1 minute at ambient temperature. After the reaction time, the reaction was quenched with water (10 mL), extracted with ethyl acetate (50 mL) and washed with water (40 mL). The water layer was extracted with ethyl acetate (2 × 30 mL). The organic layers were combined and dried over anhydrous Na_2SO_4. The drying agent was removed by filtration and the solvent was evaporated under reduced pressure to obtain the desired phenol compound **2b** (3.65 g, 90%) as a pale-yellow crystals**.** **^{1}H NMR (400 MHz, $CDCl_3$)** δ 7.59 – 7.52 (m, 2H), 6.98 – 6.91 (m, 2H), 6.86 (s, 1H). **^{13}C NMR (101 MHz, $CDCl_3$)** δ 160.37, 134.39, 119.32, 116.54, 102.90. **HR-MS** Calcd (M-H)$^-$ for $C_7H_4NO^-$ 118.0298; Found 118.0302.

Example: 2

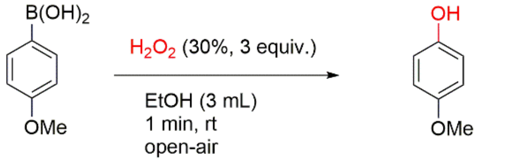

1g **2g (85%)**

In a RB-flask (25 mL), arylphenylboronic acid **1g** (5 g, 32.89 mmol) was dissolved in EtOH (3 mL). To the stirred solution, H_2O_2 (30%, 10 mL, 98.67 mmol) was added and stirred for 1 minute at ambient temperature. After the reaction time, the reaction was quenched with water (10 mL), extracted with ethyl acetate (50 mL) and washed with water (40 mL). The water layer was extracted with ethyl acetate (2 × 30 mL). The organic layers were combined and dried over anhydrous Na_2SO_4. The drying agent was removed by filtration and the solvent was evaporated under reduced pressure to obtain the desired phenol compound **2g** (3.46 g, 85%) as a brown solid**.** **^{1}H NMR (400 MHz, $CDCl_3$)** δ 6.83 – 6.72 (m, 4H), 4.90 (s, 1H), 3.77 (s, 3H). **^{13}C NMR (101 MHz, $CDCl_3$)** δ 153.74, 149.51, 116.11, 114.93, 55.87. **HR-MS** Calcd (M-H)$^-$ for $C_7H_7O_2^-$ 123.0452; Found 123.0454.

Example: 3

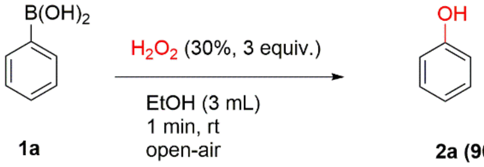

1a **2a (90%)**

In a RB-flask (25 mL), arylphenylboronic acid **1a** (5 g, 41 mmol) was dissolved in EtOH (3 mL). To the stirred solution, H_2O_2 (30%, 12.5 mL,123 mmol) was added and stirred for 1 minute at ambient temperature. After the reaction time, the reaction was quenched with water (10 mL), extracted with ethyl acetate (50 mL) and washed with water (40 mL). The water layer was extracted with ethyl acetate (2 × 30 mL). The organic layers were combined and dried over anhydrous Na_2SO_4. The drying agent was removed by filtration and the solvent was evaporated under reduced pressure to obtain the desired phenol compound **2a** (3.48 g, 90%) as a brown solid**.** **^{1}H NMR (400 MHz, $CDCl_3$)** δ 7.30 – 7.22 (m, 2H), 6.95 (t, *J* = 7.4 Hz, 1H), 6.85 (d, *J* = 7.9 Hz, 2H), 4.77 (s, 1H). **^{13}C NMR (101 MHz, $CDCl_3$)** δ 155.41, 129.71, 120.87, 115.30. **HR-MS** Calcd (M-H)$^-$ for $C_6H_5O^-$ 93.0346; Found 93.0351.

Aryl-phenol derivatives [2a-w]:

Phenol (2a) [108-95-2].

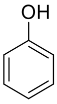

Following the general procedure (a), phenylboronic acid **1a** (150 mg, 1.23 mmol), H_2O_2 (30%, 0.38 mL, 3.69 mmol, 3 equiv.). The desired product **2a** obtained as a white solid (107 mg, 92%). R_f = 0.33 [(EtOAc:Heptane, 20:80)]. **^{1}H NMR (400 MHz, $CDCl_3$)** δ 7.30 – 7.19 (m, 2H), 6.94 (tt, *J* = 7.4, 1.1 Hz, 1H), 6.88 – 6.79 (m, 2H), 4.79 (s, 1H). **^{13}C NMR (101 MHz, $CDCl_3$)** δ 155.47, 129.69, 120.82, 115.29. **HR-MS** Calcd $(M-H)^-$ for $C_6H_5O^-$ 93.0346; Found 93.0351. **GC-MS**: 95 (8), 94 (100, M^+), 66 (72), 65 (46), 63 (10), 50 (8).

4-hydroxybenzonitrile (2b) [767-00-0]

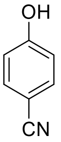

Following the general procedure (a), 4-cyanophenylboronic acid **1b** (184 mg, 1.23 mmol), H_2O_2 (30%, 0.38 mL, 3.69 mmol, 3 equiv.). The desired product **2b** obtained as a pale-white solid (132 mg, 90%). R_f = 0.50 [(EtOAc:Pentane, 40:60)]. **^{1}H NMR (400 MHz, $CDCl_3$)** δ 7.71 – 7.44 (m, 2H), 7.06 – 6.86 (m, 2H), 6.53 (s, 1H). **^{13}C NMR (101 MHz, $CDCl_3$)** δ 160.10, 134.37, 119.27, 116.48, 103.22. **HR-MS** Calcd $(M-H)^-$ for $C_7H_4NO^-$ 118.0298; Found 118.0302.

4-chlorophenol (2c) [106-48-9]

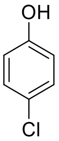

Following the general procedure (a), 4-chlorophenylboronic acid **1c** (192 mg, 1.23 mmol), H_2O_2 (30%, 0.38 mL, 3.69 mmol, 3 equiv.). The desired product **2c** obtained as a brownish liquid (130 mg, 82%). R_f = 0.75 [(EtOAc:Pentane, 40:60)]. **^{1}H NMR (400 MHz, MeOD)** δ 7.18 – 7.10 (m, 2H), 6.79 – 6.71 (m, 2H). **^{13}C NMR (101 MHz, MeOD)** δ 157.39, 130.19, 125.05, 117.64. **HR-MS** Calcd $(M-H)^-$ for $C_6H_4ClO^-$ 126.9956; Found 126.9960.

***O*-cresol (2d) [95-48-7]**

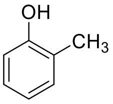

Following the general procedure (a), 2-methylphenylboronic acid **1d** (167 mg, 1.23 mmol), H_2O_2 (30%, 0.38 mL, 3.69 mmol, 3 equiv.). The desired product **2d** obtained as a brownish liquid (110 mg, 83%). R_f = 0.69 [(EtOAc:Pentane, 30:70)]. **^{1}H NMR (400 MHz, Chloroform-*d*)** δ 7.16 – 7.06 (m, 2H), 6.82 (dt, *J* = 33.5, 5.9 Hz, 2H), 4.68 (s, 1H), 2.26 (s, 3H). **^{13}C NMR (101 MHz, $CDCl_3$)** δ 153.75, 131.03, 127.12, 120.79, 114.91, 15.72. **HR-MS** Calcd $(M-H)^-$ for $C_7H_7O^-$ 107.0502; Found 107.0508.

3-nitrophenol (2e) [554-84-7]

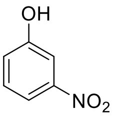

Following the general procedure (a), 3-nitrophenylboronic acid **1e** (170 mg, 1.23 mmol), H_2O_2 (30%, 0.38 mL, 3.69 mmol, 3 equiv.). The crude product was isolated using [(EtOAc:Pentane, 20:80)] and obtained the compound **2e** as a pale-white solid (85 mg, 60%). R_f = 0.45 [(EtOAc:Pentane, 30:70)]. **^{1}H NMR (400 MHz, DMSO)** δ 10.43 (s, 1H), 7.66 (dd, *J* = 8.5, 2.3 Hz, 1H), 7.55 (t, *J* = 2.3 Hz, 1H), 7.47 (t, *J* = 8.2 Hz, 1H), 7.26 – 7.18 (m, 1H). **^{13}C NMR (101 MHz, DMSO)** δ 158.70, 149.13, 131.10, 122.86, 114.27, 110.02. **HR-MS** Calcd $(M-H)^-$ for $C_6H_4NO_3^-$ 138.0197; Found 138.0199.

4-bromophenol (2f) [106-41-2]

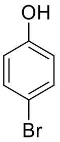

Following the general procedure (a), 4-bromophenylboronic acid **1f** (247 mg, 1.23 mmol), H_2O_2 (30%, 0.38 mL, 3.69 mmol, 3 equiv.). The desired product **2f** obtained as a yellowish liquid (178 mg, 84%). R_f = 0.54 [(EtOAc:Pentane, 40:60)]. **^{1}H NMR (400 MHz, Chloroform-*d*)** δ 7.40 – 7.29 (m, 2H), 6.78 – 6.69 (m, 2H), 4.92 (s, 1H). **^{13}C NMR (101 MHz, Chloroform-*d*)** δ 154.57, 132.52, 117.23, 112.97. **HR-MS** Calcd (M-H)$^-$ for $C_6H_4BrO^-$ 170.9451; Found 170.9454.

4-methoxyphenol (2g) [150-76-5]

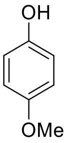

Following the general procedure (a), 4-methoxyphenylboronic acid **1g** (187 mg, 1.23 mmol), H_2O_2 (30%, 0.38 mL, 3.69 mmol, 3 equiv.). The desired product **2g** obtained as a brown solid (151 mg, 94%). R_f = 0.65 [(EtOAc:Pentane, 40:60)]. **^{1}H NMR (400 MHz, MeOD)** δ 6.79 – 6.68 (m, 4H), 3.72 (s, 3H). **^{13}C NMR (101 MHz, MeOD)** δ 154.48, 152.20, 116.77, 115.72, 56.14. **HR-MS** Calcd (M-H)$^-$ for $C_7H_7O_2^-$ 123.0452; Found 123.0454.

1-(4-hydroxyphenyl)ethan-1-one (2h) [99-93-4]

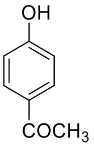

Following the general procedure (a), (4-acetylphenyl)boronic acid **1h** (202 mg, 1.23 mmol), H_2O_2 (30%, 0.38 mL, 3.69 mmol, 3 equiv.). The desired product **2h** obtained as a brown solid (160 mg, 96%). R_f = 0.60 [(EtOAc:Pentane, 50:50)]. **^{1}H NMR (400 MHz, DMSO)** δ 10.32 (s, 1H), 7.97 – 7.69 (m, 2H), 6.92 – 6.76 (m, 2H), 2.47 (s, 3H). **^{13}C NMR (101 MHz, DMSO)** δ 196.49, 162.46, 131.18, 129.06, 115.61, 26.72. **HR-MS** Calcd (M-H)$^-$ for $C_8H_7O_2^-$ 135.0452; Found 135.0455.

2,4,6-trimethylphenol (2i) [527-60-6]

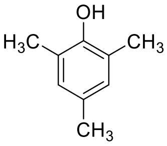

Following the general procedure (a), 2,4,6-trimethylphenylboronic acid **1i** (202 mg, 1.23 mmol), H_2O_2 (30%, 0.38 mL, 3.69 mmol, 3 equiv.). The desired product **2i** obtained as a white solid (160 mg, 95%). **^{1}H NMR (400 MHz, DMSO)** δ 7.89 (s, 1H), 6.70 (s, 2H), 2.13 (d, *J* = 5.0 Hz, 9H). **^{13}C NMR (101 MHz, DMSO)** δ 150.84, 128.72, 127.41, 124.05, 20.15, 16.61. **HR-MS** Calcd (M-H)$^-$ for $C_9H_{11}O^-$ 135.0815; Found 135.0818.

4-hydroxybenzaldehyde (2j) [123-08-0]

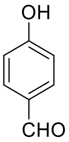

Following the general procedure (a), 4-formylphenylboronic acid **1j** (164 mg, 1.09 mmol), H_2O_2 (30%, 0.34 mL, 3.27 mmol, 3 equiv.). The desired product **2j** obtained as a white solid (125 mg, 94%). **^{1}H NMR (400 MHz, DMSO)** δ 10.59 (s, 1H), 9.78 (s, 1H), 7.85 – 7.65 (m, 2H), 7.02 – 6.84 (m, 2H). **^{13}C NMR (101 MHz, DMSO-d_6)** δ 190.99, 163.34, 132.14, 128.47, 115.87. **HR-MS** Calcd (M-H)$^-$ for $C_7H_5O_2^-$ 121.0295; Found 121.0299.

4-hydroxybenzoic acid (2k) [99-96-7]

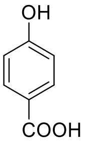

Following the general procedure (a), 4-carboxyphenylboronic acid **1k** (204 mg, 1.23 mmol), H_2O_2 (30%, 0.38 mL, 3.69 mmol, 3 equiv.). The desired product **2k** obtained as a white solid (165 mg, 97%). R_f = 0.42 [(EtOAc: 100)]. **^{1}H NMR (400 MHz, DMSO-*d*$_6$)** δ 12.46 (s, 1H), 10.21 (s, 1H), 7.83 – 7.74 (m, 2H), 6.88 – 6.76 (m, 2H). **^{13}C NMR (101 MHz, DMSO-*d*$_6$)** δ 167.21, 161.64, 131.57, 121.39, 115.16. **HR-MS** Calcd $(M-H)^-$ for $C_7H_5O_3^-$ 137.0244; Found 137.0247.

4-ethylphenol (2l) [123-07-9]

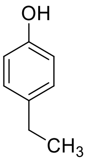

Following the general procedure (a), 4-ethylphenylboronic acid **1l** (184 mg, 1.23 mmol), H_2O_2 (30%, 0.38 mL, 3.69 mmol, 3 equiv.). The desired product **2l** obtained as a brownish liquid (147 mg, 98%). R_f = 0.80 [(EtOAc:Pentane, 30:70)]. **^{1}H NMR (400 MHz, DMSO-*d*$_6$)** δ 9.04 (s, 1H), 7.02 – 6.94 (m, 2H), 6.70 – 6.62 (m, 2H), 2.46 (q, *J* = 7.6 Hz, 2H), 1.12 (t, *J* = 7.6 Hz, 3H). **^{13}C NMR (101 MHz, DMSO-*d*$_6$)** δ 155.67, 134.27, 128.97, 115.48, 27.79, 16.49. **HR-MS** Calcd $(M-H)^-$ for $C_8H_9O^-$ 121.0659; Found 121.0663.

2,3-dimethylphenol (2m) [526-75-0]

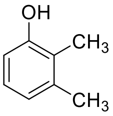

Following the general procedure (a), 2,3-dimethylphenylboronic acid **1m** (184 mg, 1.23 mmol), H_2O_2 (30%, 0.38 mL, 3.69 mmol, 3 equiv.). The desired product **2m** obtained as a brownish liquid (145 mg, 97%). R_f = 0.83 [(EtOAc:Pentane, 20:80)]. **^{1}H NMR (400 MHz, Chloroform-*d*)** δ 6.97 (t, *J* = 7.8 Hz, 1H), 6.76 (d, *J* = 7.5 Hz, 1H), 6.64 (d, *J* = 8.0 Hz, 1H), 4.67 (s, 1H), 2.28 (s, 3H), 2.17 (s, 3H). **^{13}C NMR (101 MHz, Chloroform-*d*)** δ 153.51, 138.35, 136.29, 126.03, 122.41, 112.63, 20.12, 11.46. **HR-MS** Calcd $(M-H)^-$ for $C_8H_9O^-$ 121.0659; Found 121.0663.

***N*-(4-hydroxyphenyl)acetamide (2n) [103-90-2]**

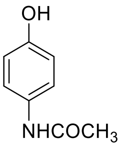

Following the general procedure (a), 4-acetamidophenylboronic acid **1n** (220 mg, 1.23 mmol), H_2O_2 (30%, 0.38 mL, 3.69 mmol, 3 equiv.) and ethanol (5 mL used in total, due to solubility issues). The desired product **2n** obtained as a brownish solid (180 mg, 97%). R_f = 0.33 [(EtOAc:100)]. **^{1}H NMR (400 MHz, DMSO-*d*$_6$)** δ 9.63 (s, 1H), 9.10 (s, 1H), 7.47 – 7.11 (m, 2H), 6.87 – 6.50 (m, 2H), 1.97 (s, 3H). **^{13}C NMR (101 MHz, DMSO-*d*$_6$)** δ 167.53, 153.14, 131.05, 120.83, 115.01, 23.76. **HR-MS** Calcd $(M-H)^-$ for $C_8H_8NO_2^-$ 150.0561; Found 150.0564.

tert-butyl (4-hydroxyphenyl)carbamate (2o) [54840-15-2]

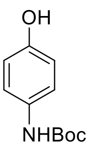

Following the general procedure (a), 4-*N*-Boc-phenylboronic acid **1o** (292 mg, 1.23 mmol), H_2O_2 (30%, 0.38 mL, 3.69 mmol, 3 equiv.) and ethanol (5 mL used in total, due to solubility issues). The desired product **2o** obtained as a brownish solid (252 mg, 98%). R_f = 0.50 [(EtOAc: Pentane, 40:60)]. **^{1}H NMR (400 MHz, DMSO-*d*$_6$)** δ 9.02 (s, 1H), 8.97 (s, 1H), 7.21 (d, *J* = 8.3 Hz, 2H), 6.71 – 6.58 (m, 2H), 1.45 (s, 9H). **^{13}C NMR (101 MHz, DMSO-*d*$_6$)** δ 153.02, 152.52, 131.03, 120.02, 115.03, 78.43, 28.21. **HR-MS** Calcd $(M-H)^-$ for $C_{11}H_{14}NO_3^-$ 208.0979; Found 208.0981.

2,6-dimethoxyphenol (2p) [91-10-1]

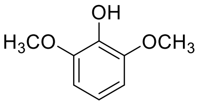

Following the general procedure (a), 2,6-dimethoxyphenylboronic acid **1p** (224 mg, 1.23 mmol), H_2O_2 (30%, 0.38 mL, 3.69 mmol, 3 equiv.). The desired product **2p** obtained as a brown solid (182 mg, 96%). R_f = 0.64 [(EtOAc: Pentane, 40:60)]. **^{1}H NMR (400 MHz, Chloroform-*d*)** δ 6.84 – 6.76 (m, 1H), 6.58 (d, *J* = 8.3 Hz, 2H), 5.53 (s, 1H), 3.88 (s, 6H). **^{13}C NMR (101 MHz, Chloroform-*d*)** δ 147.27, 134.86, 119.09, 104.92, 56.28. **HR-MS** Calcd $(M+H)^+$ for $C_8H_{10}NaO_3^+$ 177.0522; Found 177.0523.

2-chlorophenol (2q) [95-57-8]

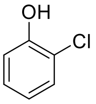

Following the general procedure (a), 2-chlorophenylboronic acid **1q** (192 mg, 1.23 mmol), H_2O_2 (30%, 0.38 mL, 3.69 mmol, 3 equiv.). The desired product **2q** obtained as a pale-white solid (150 mg, 95%). **^{1}H NMR (400 MHz, DMSO-*d*$_6$)** δ 10.10 (s, 1H), 7.31 (dd, *J* = 7.9, 1.6 Hz, 1H), 7.14 (td, *J* = 7.7, 1.6 Hz, 1H), 6.96 (dd, *J* = 8.1, 1.6 Hz, 1H), 6.79 (td, *J* = 7.6, 1.6 Hz, 1H). **^{13}C NMR (101 MHz, DMSO-*d*$_6$)** δ 153.52, 130.32, 128.49, 120.47, 120.07, 117.14. **HR-MS** Calcd $(M-H)^-$ for $C_6H_4ClO^-$ 126.9956; Found 126.9960.

3-methoxyphenol (2r) [150-19-6]

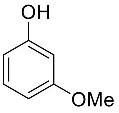

Following the general procedure (a), 3-methoxyphenylboronic acid **1r** (187 mg, 1.23 mmol), H_2O_2 (30%, 0.38 mL, 3.69 mmol, 3 equiv.). The desired product **2r** obtained as a brownish liquid (140 mg, 92%). R_f = 0.42 [(EtOAc: Pentane, 30:70)]. **^{1}H NMR (400 MHz, Chloroform-*d*)** δ 7.14 (t, *J* = 8.1 Hz, 1H), 6.51 (d, *J* = 8.4 Hz, 1H), 6.47 – 6.41 (m, 2H), 5.21 (s, 1H), 3.78 (s, 3H). **^{13}C NMR (101 MHz, Chloroform-*d*)** δ 160.89, 156.70, 130.20, 107.90, 106.53, 101.60, 55.34. **HR-MS** Calcd $(M-H)^-$ for $C_7H_7O_2^-$ 123.0452; Found 123.0455.

2-bromophenol (2s) [95-56-7]

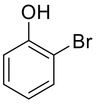

Following the general procedure (a), 2-bromophenylboronic acid **1s** (247 mg, 1.23 mmol), H_2O_2 (30%, 0.38 mL, 3.69 mmol, 3 equiv.). The desired product **2s** obtained as a brown solid (188 mg, 88%). R_f = 0.74 [(EtOAc: Pentane, 30:70)]. **^{1}H NMR (400 MHz, Chloroform-*d*)** δ 7.46 (dd, *J* = 8.0, 1.6 Hz, 1H), 7.22 (ddd, *J* = 8.7, 7.3, 1.6 Hz, 1H), 7.03 (dd, *J* = 8.1, 1.6 Hz, 1H), 6.81 (td, *J* = 7.6, 1.5 Hz, 1H), 5.49 (s, 1H). **^{13}C NMR (101 MHz, Chloroform-*d*)** δ 152.27, 132.03, 129.22, 121.84, 116.15, 110.28. **HR-MS** Calcd $(M-H)^-$ for $C_6H_4BrO^-$ 170.9451; Found 170.9454.

3-bromophenol (2t) [591-20-8]

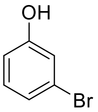

Following the general procedure (a), 3-bromophenylboronic acid **1t** (247 mg, 1.23 mmol), H_2O_2 (30%, 0.38 mL, 3.69 mmol, 3 equiv.). The desired product **2t** obtained as a brown solid (170 mg, 80%). R_f = 0.71 [(EtOAc: Pentane, 30:70)]. **^{1}H NMR (400 MHz, DMSO-*d*$_6$)** δ 9.86 (s, 1H), 7.12 (t, *J* = 8.3 Hz, 1H), 6.98 – 6.91 (m, 2H), 6.76 (ddd, *J* = 8.2, 2.2, 1.1 Hz, 1H). **^{13}C NMR (101 MHz, DMSO-*d*$_6$)** δ 159.08, 131.64, 122.30, 122.13, 118.58, 115.05 **HR-MS** Calcd $(M-H)^-$ for $C_6H_4BrO^-$ 170.9451; Found 170.9454.

Thiophen-2(5*H*)-one (2u) [3354-32-3]

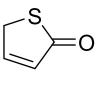

Following the general procedure (a), 2-thienylboronic acid **1u** (157 mg, 1.23 mmol), H_2O_2 (30%, 0.38 mL, 3.69 mmol, 3 equiv.). The desired product **2u** obtained as a yellowish liquid (98 mg, 80%). **^{1}H NMR (400 MHz, Chloroform-*d*)** δ

7.57 (dt, *J* = 5.8, 2.8 Hz, 1H), 6.39 (dt, *J* = 6.1, 2.1 Hz, 1H), 4.22 – 4.00 (m, 2H). **^{13}C NMR (101 MHz, Chloroform-*d*)** δ 200.51, 153.89, 133.54, 38.58.

1*H*-indol-5-ol (2v) [1953-54-4]

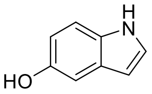

Following the general procedure (a), 5-indolylboronic acid **1v** (198 mg, 1.23 mmol), H_2O_2 (30%, 0.38 mL, 3.69 mmol, 3 equiv.). The desired product **2v** obtained as a brown solid (150 mg, 91%). **^{1}H NMR (400 MHz, DMSO-*d*$_6$)** δ 10.74 (s, 1H), 8.60 (s, 1H), 7.26 – 7.16 (m, 2H), 6.87 (d, *J* = 2.4 Hz, 1H), 6.62 (dd, *J* = 8.6, 2.3 Hz, 1H), 6.24 (t, *J* = 2.5 Hz, 1H). **^{13}C NMR (101 MHz, DMSO-*d*$_6$)** δ 150.55, 130.51, 128.44, 125.53, 111.66, 111.34, 103.90, 100.27.

pyren-1-ol (2w) [5315-79-7]

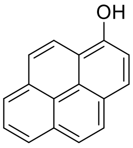

Following the general procedure (a), pyrene-1-boronic acid **1w** (303 mg, 1.23 mmol), H_2O_2 (30%, 0.38 mL, 3.69 mmol, 3 equiv.). The desired product **2w** obtained as a brown solid (262 mg, 98%). R_f = 0.50 [(EtOAc: Pentane, 30:70)]. **^{1}H NMR (400 MHz, Chloroform-*d*)** δ 8.34 (d, *J* = 9.2 Hz, 1H), 8.10 (t, *J* = 7.5 Hz, 2H), 8.04 (t, *J* = 8.3 Hz, 2H), 8.00 – 7.94 (m, 2H), 7.90 (d, *J* = 8.9 Hz, 1H), 7.46 (d, *J* = 8.2 Hz, 1H), 5.60 (s, 1H). **^{13}C NMR (101 MHz, CDCl$_3$)** δ 149.71, 131.76, 131.64, 127.43, 127.28, 126.62, 126.22, 126.09, 125.68, 125.54, 125.02, 124.52, 124.25, 120.52, 118.66, 113.07. **HR-MS** Calcd (M-H)$^-$ for $C_{16}H_9O^-$ 217.0659; Found 217.0659.

Aryl-halophenol derivatives [3a-k]

2,4,6-tribromophenol (3a) [118-79-6]

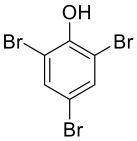

Following the general procedure (b), phenylboronic acid **1a** (150 mg, 1.23 mmol), H_2O_2 (30%, 0.38 mL, 3.69 mmol, 3 equiv.) and HBr (62%, 0.28 mL, 3.69 mmol, 3 equiv.). The desired product **3a** obtained as a pale-white solid (385 mg, 95%) without column purification. **^{1}H NMR (400 MHz, CDCl$_3$)** δ 7.58 (s, 2H), 5.86 (s, 1H). **^{13}C NMR (101 MHz, CDCl$_3$)** δ 148.96, 135.66, 134.25, 128.00, 112.72, 110.42. **HR-MS** Calcd (M-H)$^-$ for $C_6H_2Br_3O^-$ 326.7661; Found 326.7661. **GC-MS**: 334 (32), 332 (90), 330 (100), 328 (30, M+), 254 (7), 253 (10), 251 (12), 250 (20), 248 (10), 226 (6), 224 (10), 223 (13), 220 (10), 143 (11), 141 (13), 63 (9), 62 (24), 61 (19).

2,4,6-tribromophenol (3b) [1689-84-5]

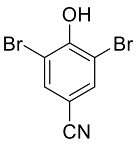

Following the general procedure (b), 4-cyanophenylboronic acid **1b** (150 mg, 1.23 mmol), H_2O_2 (30%, 0.38 mL, 3.69 mmol, 3 equiv.) and HBr (62%, 0.28 mL, 3.69 mmol, 3 equiv.). The crude product was isolated using [(EtOAc: Pentane, 30:70)] eluent and obtained the compound **3b** as a white solid (300 mg, 89%). R_f = 0.35 [(EtOAc: Pentane, 40:60)]. **^{1}H NMR (400 MHz, DMSO-*d*$_6$)** δ 11.28 (s, 1H), 8.13 (s, 2H). **^{13}C NMR (101 MHz, DMSO-*d*$_6$)** δ 155.96, 136.67, 117.23, 112.29, 104.94. **HR-MS** Calcd (M-H)$^-$ for $C_7H_2Br_2NO^-$ 273.8509; Found 273.8509.

2,6-dibromo-4-chlorophenol (3c) [5324-13-0]

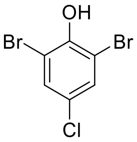

Following the general procedure (b), 4-chlorophenylboronic acid **1c** (192 mg, 1.23 mmol), H_2O_2 (30%, 0.38 mL, 3.69 mmol, 3 equiv.) and HBr (62%, 0.28 mL, 3.69 mmol, 3 equiv.). The crude product was isolated using [(EtOAc: Pentane, 10:90)] eluent and obtained the compound **3c** as a pale-yellow solid (230 mg, 65%). R_f = 0.87 [(EtOAc: Pentane, 40:60)]. **^{1}H NMR (400 MHz, MeOD)** δ 7.51 (s, 2H). **^{13}C NMR (101 MHz, MeOD)** δ 151.77, 132.74, 126.31, 112.58. **HR-MS** Calcd $(M-H)^-$ for $C_6H_2Br_2ClO^-$ 282.8166; Found 282.8166.

2,4-dibromo-6-methylphenol (3d) [609-22-3]

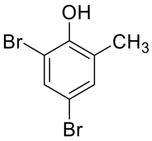

Following the general procedure (b), *o*-tolylboronic acid **1d** (80 mg, 0.59 mmol), H_2O_2 (30%, 0.18 mL, 3 equiv.) and HBr (62%, 0.1 mL, 2.2 equiv.). The crude product was isolated using [(Pentane, 100%)] eluent and obtained the compound **3d** as a pale-white solid (95 mg, 60%). R_f = 0.75 [(EtOAc: Pentane, 30:70)]. **^{1}H NMR (400 MHz, $CDCl_3$)** δ 7.43 (d, *J* = 2.3 Hz, 1H), 7.20 (d, *J* = 2.3 Hz, 1H), 5.52 (s, 1H), 2.27 (s, 3H). **^{13}C NMR (101 MHz, $CDCl_3$)** δ 149.77, 133.09, 131.28, 127.69, 112.05, 110.44, 16.56. **HR-MS** Calcd $(M-H)^-$ for $C_7H_5Br_2O^-$ 262.8713; Found 262.8714.

1-(3,5-dibromo-4-hydroxyphenyl)ethan-1-one (3e) [2887-72-1]

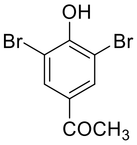

Following the general procedure (b), 4-acetylphenylboronic acid **1h** (202 mg, 1.23 mmol), H_2O_2 (30%, 0.38 mL, 3 equiv.) and HBr (62%, 0.2 mL, 2.2 equiv.). The crude product was isolated using [(EtOAc: Pentane, 30:70)]eluent and obtained the compound **3e** as a white solid (185 mg, 52%). R_f = 0.41 [(EtOAc: Pentane, 40:60)]. **^{1}H NMR (400 MHz, Methanol-*d*$_4$)** δ 8.09 (s, 2H), 2.52 (s, 3H). **^{13}C NMR (101 MHz, MeOD)** δ 196.72, 156.75, 134.03, 132.32, 111.95, 26.28. **HR-MS** Calcd $(M-H)^-$ for $C_8H_5Br_2O_2^-$ 290.8662; Found 290.8659.

2,4,6-tribromophenol (3f) [118-79-6]

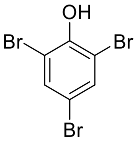

Following the general procedure (b), **4-bromophenylboronic acid** (**1f**) (247 mg, 1.23 mmol), H_2O_2 (30%, 0.38 mL, 3.69, 3 equiv.) and HBr (62%, 0.28 mL, 3.69 mmol, 3 equiv.). The desired product **3f** obtained as a pale-white solid (325 mg, 80%) without column purification. R_f = 0.79 [(EtOAc: Pentane, 20:80)]. **^{1}H NMR (400 MHz, Chloroform-*d*)** δ 7.57 (s, 2H), 5.90 (s, 1H). **^{13}C NMR (101 MHz, Chloroform-*d*)** δ 149.03, 134.31, 112.81, 110.52. **HR-MS** Calcd $(M-H)^-$ for $C_6H_2Br_3O^-$ 326.7661; Found 326.7659.

2,6-dibromo-4-ethylphenol (3g) [57018-12-9]

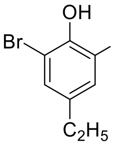

Following the general procedure (b), 4-ethylphenylboronic acid (**1l**) (247 mg, 1.23 mmol), H_2O_2 (30%, 0.38 mL, 3.69, 3 equiv.) and HBr (62%, 0.28 mL, 3.69 mmol, 3 equiv.). The crude product was isolated using [(EtOAc: Pentane, 10:90)] eluent and obtained the compound **3g** as a pale-yellow solid (180 mg, 53%). R_f = 0.82 [(EtOAc: Pentane, 30:70)]. **^{1}H NMR (400 MHz, Chloroform-*d*)** δ 7.27 (d, *J* = 0.7 Hz, 2H), 5.71 (s, 1H), 2.55 (q, *J* = 7.6 Hz, 1H), 1.20 (t, *J* = 7.6 Hz, 3H). **^{13}C NMR**

(101 MHz, Chloroform-*d*) δ 147.24, 138.85, 131.33, 109.55, 27.54, 15.46. **HR-MS** Calcd (M-H)$^-$ for $C_8H_7Br_2O^-$ 276.8869; Found 276.8867.

4,6-dibromo-2,3-dimethylphenol (3h) [15460-16-9]

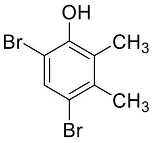

Following the general procedure (b), 2,3-dimethylphenylboronic acid (**1m**) (184 mg, 1.23 mmol), H_2O_2 (30%, 0.38 mL, 3.69, 3 equiv.) and HBr (62%, 0.28 mL, 3.69 mmol, 3 equiv.). The desired product **3h** obtained as a pale-yellow solid (300 mg, 88%) without column purification. R_f = 0.84 [(EtOAc: Pentane, 20:80)]. **^{1}H NMR (400 MHz, Chloroform-*d*)** δ 7.51 (s, 1H), 5.44 (s, 1H), 2.32 (s, 3H), 2.28 (s, 3H). **^{13}C NMR (101 MHz, Chloroform-*d*)** δ 149.44, 137.19, 131.28, 125.75, 115.66, 107.57, 19.77, 14.06. **HR-MS** Calcd (M-H)$^-$ for $C_8H_7Br_2O^-$ 276.8869; Found 276.88963.

2,4-dibromo-6-chlorophenol (3i) [4526-56-1]

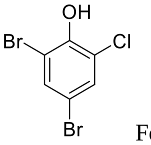

Following the general procedure (b), 2-chlorophenylboronic acid (**1q**) (192 mg, 1.23 mmol), H_2O_2 (30%, 0.38 mL, 3.69, 3 equiv.) and HBr (62%, 0.28 mL, 3.69 mmol, 3 equiv.). The crude product was isolated using [(EtOAc: Pentane, 10:90)] eluent and obtained the compound **3i** obtained as a white solid (192 mg, 55%). R_f = 0.68 [(EtOAc: Pentane, 20:80)]. **^{1}H NMR (400 MHz, Chloroform-*d*)** δ 7.55 (d, *J* = 2.3 Hz, 1H), 7.44 (d, *J* = 2.3 Hz, 1H), 5.86 (s, 1H). **^{13}C NMR (101 MHz, Chloroform-*d*)** δ 148.19, 133.61, 131.44, 121.53, 112.26, 110.83. **HR-MS** Calcd (M-H)$^-$ for $C_6H_2Br_2ClO^-$ 282.8166; Found 282.8156.

2,3,4,6-tetrabromophenol (3j) [14400-94-3]

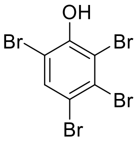

Following the general procedure (b), 3-bromophenylboronic acid (**1t**) (247 mg, 1.23 mmol), H_2O_2 (30%, 0.38 mL, 3.69, 3 equiv.) and HBr (62%, 0.28 mL, 3.69 mmol, 3 equiv.). The crude product was isolated using [(EtOAc: Pentane, 10:90)] eluent and obtained the compound **3j** obtained as a white solid (261 mg, 52%). R_f = 0.50 [(EtOAc: Pentane, 20:80)]. **^{1}H NMR (400 MHz, Chloroform-*d*)** δ 7.78 (s, 1H), 6.03 (s, 1H). **^{13}C NMR (101 MHz, Chloroform-*d*)** δ 149.91, 134.88, 127.00, 115.98, 114.27, 108.74. **HR-MS** Calcd (M-H)$^-$ for $C_6HBr_4O^-$ 404.6766; Found 404.6784.

N-(3,5-dibromo-4-hydroxyphenyl)acetamide (3k) [63558-07-6] & *N*-(4-bromophenyl)acetamide (3k') [103-88-8]

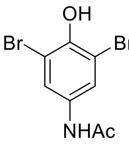

3k

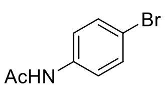

3k'

Following the general procedure (b), 4-acetamidophenylboronic acid (**1n**) (220 mg, 1.23 mmol), H_2O_2 (30%, 0.38 mL, 3.69, 3 equiv.) and HBr (62%, 0.28 mL, 3.69 mmol, 3 equiv.) ethanol (5 mL used in total, due to solubility issues). The crude product was isolated using [(EtOAc: Pentane, 80:20)] eluent and obtained the compound **3k** as a pale-yellow solid (205 mg, 54%) and in addition ipso-brominated compound **3k'** (75 mg, 28%) was obtained as a pale-white solid. **^{1}H NMR (400 MHz, DMSO-*d*$_6$)** δ 9.96 (s, 1H), 9.63 (s, 1H), 7.78 (s, 2H), 2.02 (s, 3H).**^{13}C NMR (101 MHz, DMSO-*d*$_6$)** δ 168.74, 146.80, 131.94, 123.00, 112.37, 24.33. **HR-MS** Calcd (M-H)$^-$ for $C_8H_6Br_2NO_2^-$ 305.8771; Found 305.8764. **Compound 3k'- ^{1}H NMR (400 MHz, DMSO-*d*$_6$)** δ 10.06 (s, 1H), 7.60 – 7.49 (m, 2H), 7.51 – 7.42 (m, 2H), 2.04 (s, 3H). **^{13}C NMR (101 MHz, DMSO)** δ 168.94, 139.14, 131.94, 121.32, 114.95, 24.49. **HR-MS** Calcd (M-H)$^-$ for $C_8H_7BrNO^-$ 211.9717; Found 211.9719.

5'-phenyl-[1,1':3',1''-terphenyl]-2'-ol (4a) [3140-01-0]

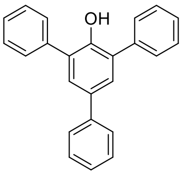

To a stirred solution of phenylboronic acid **1a** (150 mg, 1.23 mmol) in EtOH (3 mL), H_2O_2 (30%, 0.38 mL, 3.69 mmol, 3 equiv.) and HBr (62%, 0.28 mL, 3.69 mmol, 3 equiv.) was added at room temperature for 1 minutes, after the reaction time, phenylboronic acid (450 mg, 3 eq), Na_2CO_3 (391 mg, 3 eq) and $Pd(PPh_3)_4$ (57 mg, 4 mol%) were added. The reaction mixture was flushed with nitrogen and irradiated under microwave at 100 ^oC for 30 minutes. After the reaction time, the reaction was quenched with water (10 mL), extracted with ethyl acetate (50 mL) and washed with water (40 mL). The water layer was extracted with ethyl acetate (2 × 30 mL). The organic layers were combined and dried over anhydrous Na_2SO_4. The drying agent was removed by filtration and the solvent was evaporated under reduced pressure to obtain the crude product and further purified by silica-gel flash column chromatography using the ethyl acetate/pentane (10:90) to obtain the desired phenol **4a** in 20% yield. **^{1}H NMR** (400 MHz, $CDCl_3$) δ 7.52 (dt, *J* = 8.1, 1.4 Hz, 5H), 7.45 – 7.37 (m, 6H), 7.32 (tt, *J* = 7.2, 5.1 Hz, 5H), 7.25 – 7.18 (m, 1H), 5.34 (s, 1H).**^{13}C NMR** (101 MHz, $CDCl_3$) δ 148.99, 140.58, 137.59, 133.90, 129.45, 129.19, 128.99, 128.84, 128.70, 127.87, 126.95, 126.85.

5',6'-dimethyl-[1,1':3',1''-terphenyl]-4'-ol (4b) [NEW]

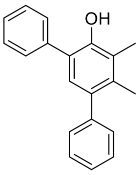

To a stirred solution of 2,3-dimethylphenylboronic acid (**1m**) (184 mg, 1.23 mmol), H_2O_2 (30%, 0.38 mL, 3.69, 3 equiv.) and HBr (62%, 0.28 mL, 3.69 mmol, 3 equiv.) was added at room temperature for 1 minutes, after the reaction time, Phenylboronic acid (750 mg, 5 eq), Na_2CO_3 (651 mg, 5 eq) and $Pd(PPh_3)_4$ (85 mg, 6 mol%) were added. The reaction mixture was flushed with nitrogen and irradiated under microwave at 100 ^oC for 30 minutes. After the reaction time, the reaction was quenched with water (10 mL), extracted with ethyl acetate (50 mL) and washed with water (40 mL). The water layer was extracted with ethyl acetate (2 × 30 mL). The organic layers were combined and dried over anhydrous Na_2SO_4. The drying agent was removed by filtration and the solvent was evaporated under reduced pressure to obtain the crude product and further purified by silica-gel flash column chromatography using the ethyl acetate/pentane (10:90) to obtain the desired phenol **4b** in 65% yield. **^{1}H NMR** (400 MHz, $CDCl_3$) δ 7.40 – 7.38 (m, 4H), 7.31 – 7.27 (m, 3H), 7.25 – 7.21 (m, 3H), 6.92 (s, 1H), 5.23 (s, 1H), 2.23 (s, 3H), 2.14 (s, 3H). **^{13}C NMR** (101 MHz, $CDCl_3$) δ 149.54, 142.38, 137.41, 135.24, 134.87, 129.74, 129.44, 129.22, 128.55, 128.03, 127.85, 126.47, 125.04, 123.56, 17.60, 12.58.

5'-(4-cyanophenyl)-2'-hydroxy-[1,1':3',1''-terphenyl]-4,4''-dicarbonitrile (4c) [NEW]

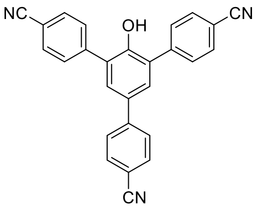

To a stirred solution of phenylboronic acid **1a** (150 mg, 1.23 mmol) in EtOH (3 mL), H_2O_2 (30%, 0.38 mL, 3.69 mmol, 3 equiv.) and HBr (62%, 0.28 mL, 3.69 mmol, 3 equiv.) was added at room temperature for 1 minutes, after the reaction time, Phenylboronic acid (750 mg, 5 eq,), Na_2CO_3 (651 mg, 5 eq) and $Pd(PPh_3)_4$ (85 mg, 6 mol%) were added. The reaction mixture was flushed with nitrogen and irradiated under microwave at 100oC for 30 minutes. After the reaction time, the reaction was quenched with water (10 mL), extracted with ethyl acetate (50 mL) and washed with water (40 mL). The water layer was extracted with ethyl acetate (2 × 30 mL). The organic layers were combined and dried over anhydrous Na_2SO_4. The drying agent was removed by filtration and the solvent was evaporated under reduced pressure to obtain the crude product and further purified by silica-gel flash column chromatography using the ethyl acetate/pentane (15:85) to obtain the desired phenol **4c** in 67% yield. **^{1}H NMR** (400 MHz, $CDCl_3$) δ 7.75 – 7.70 (m, 1H), 7.67 – 7.60 (m, 1H), 7.51 – 7.43 (m, 6H), 6.92 – 6.84 (m, 6H).**^{13}C NMR** (101 MHz, $CDCl_3$) δ 160.62, 134.37, 133.02, 128.06, 119.38, 116.56, 102.62.

^{1}H-NMR

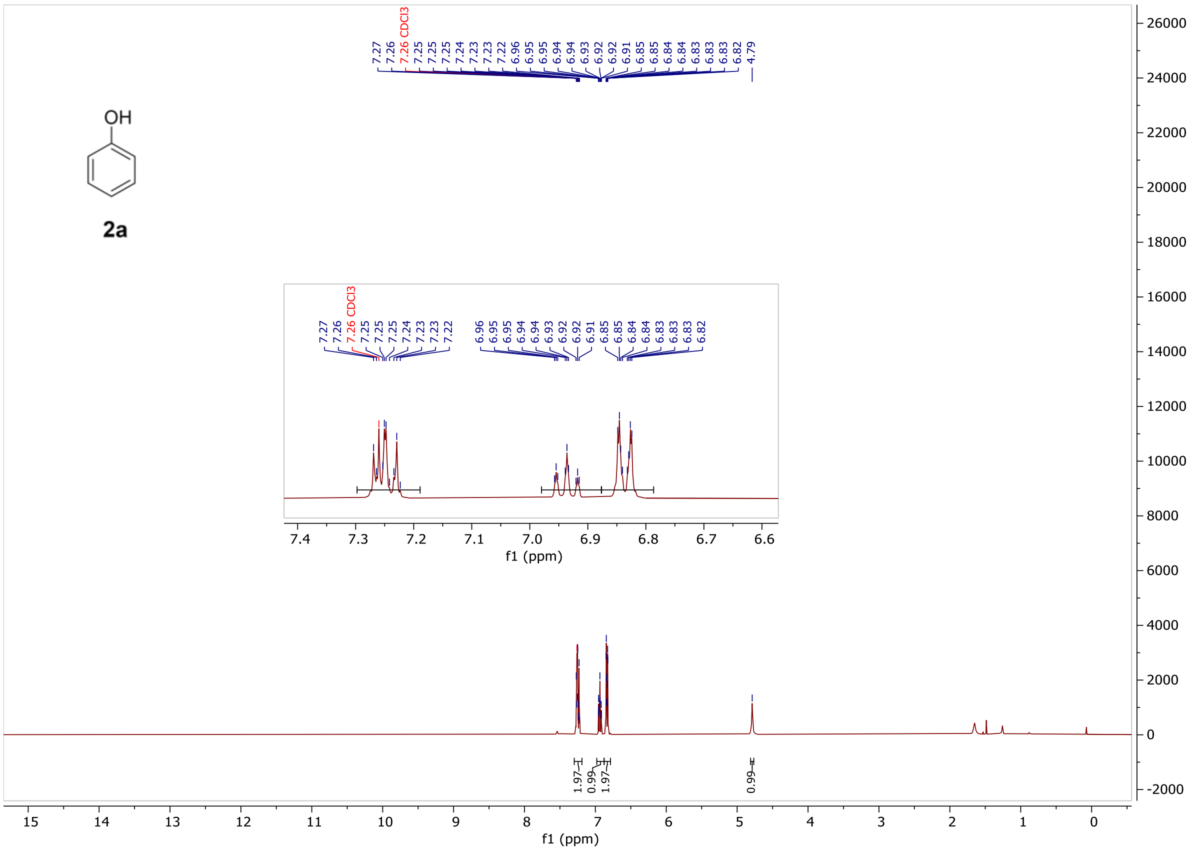

^{13}C-NMR

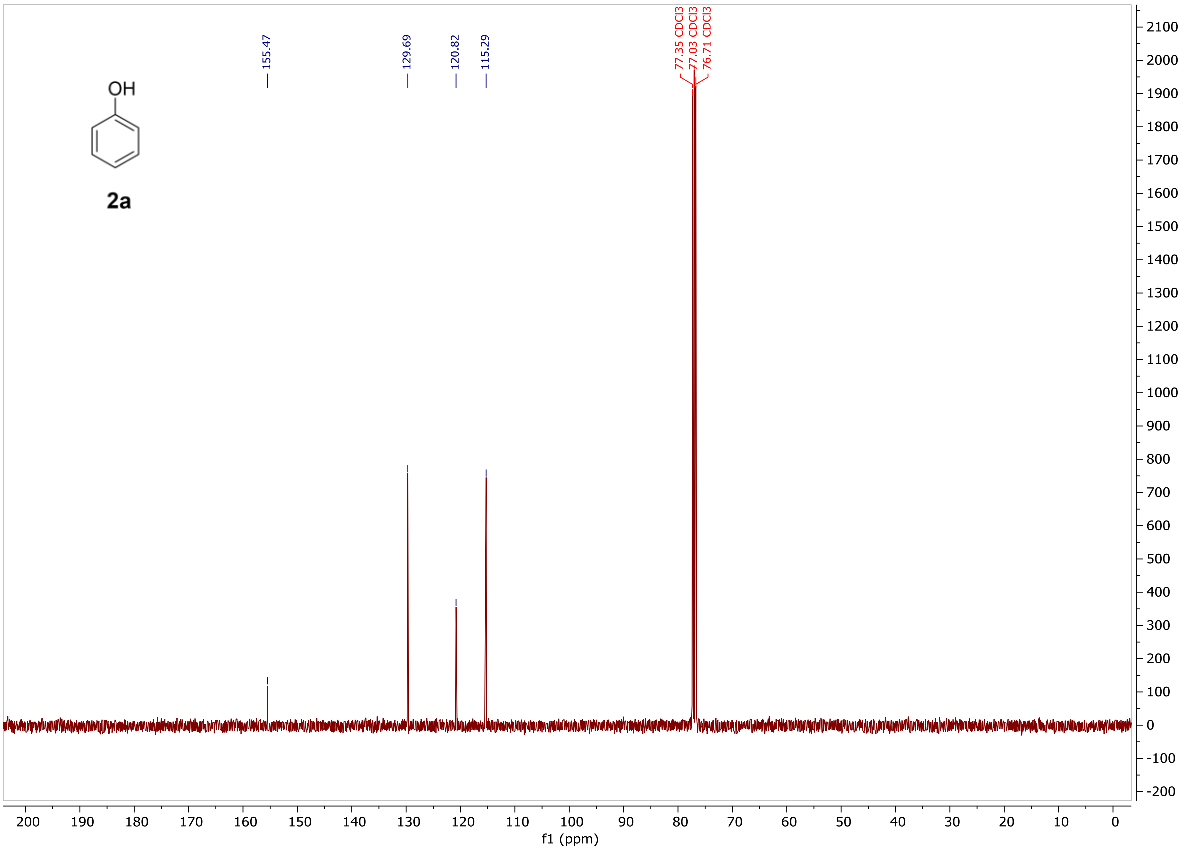

^{1}H-NMR

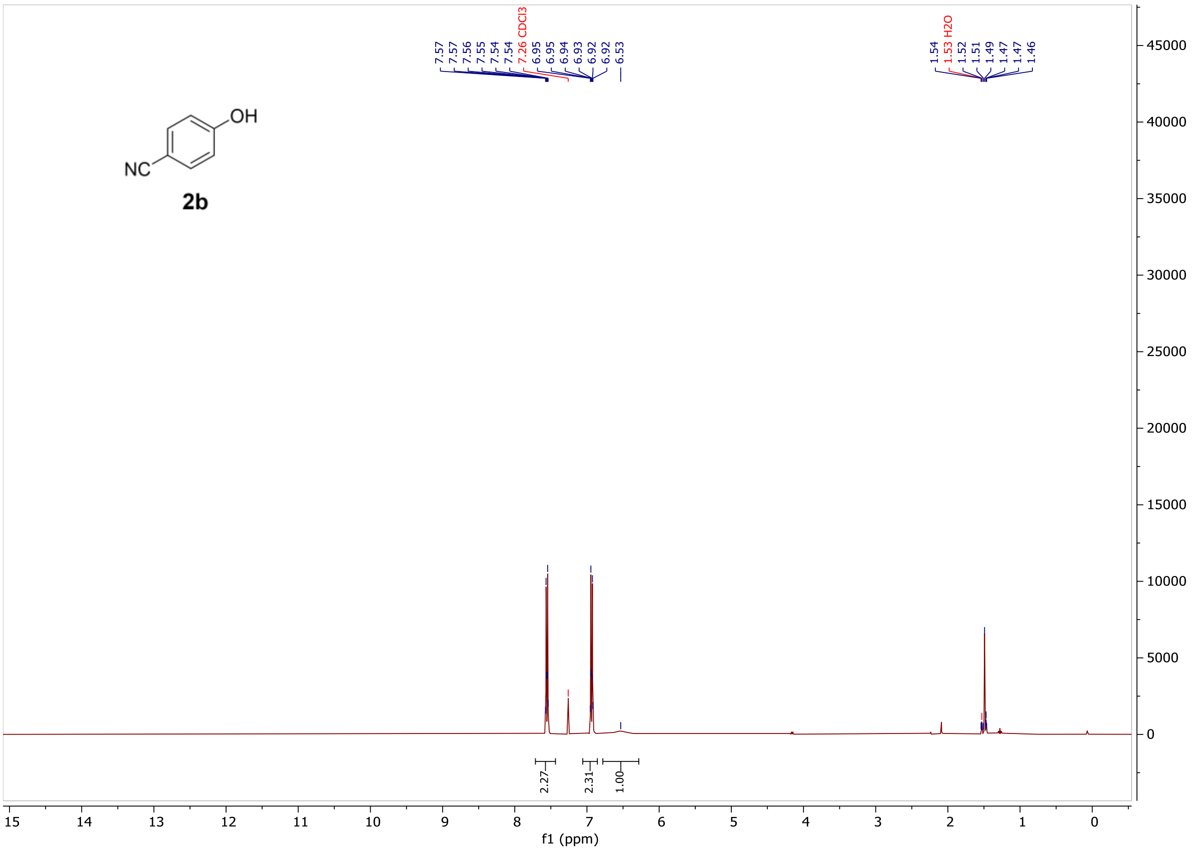

^{13}C-NMR

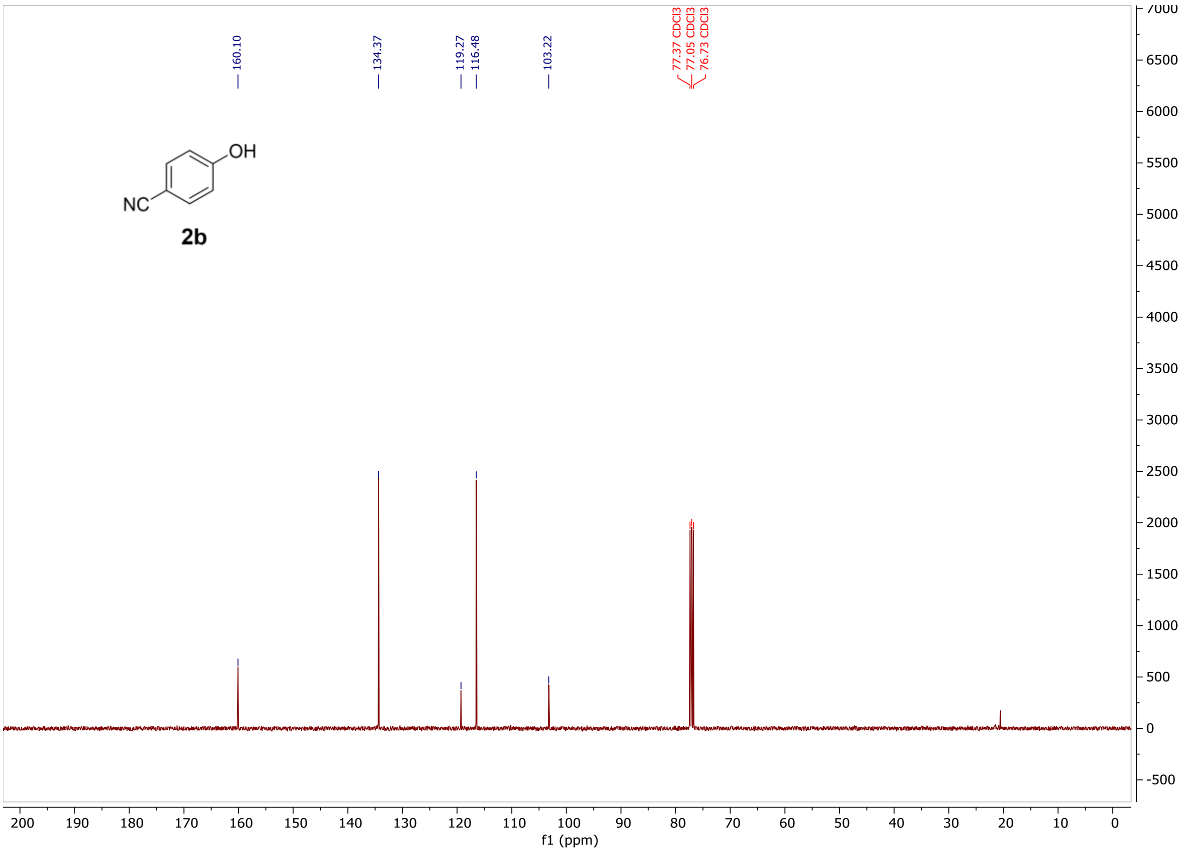

^{1}H-NMR

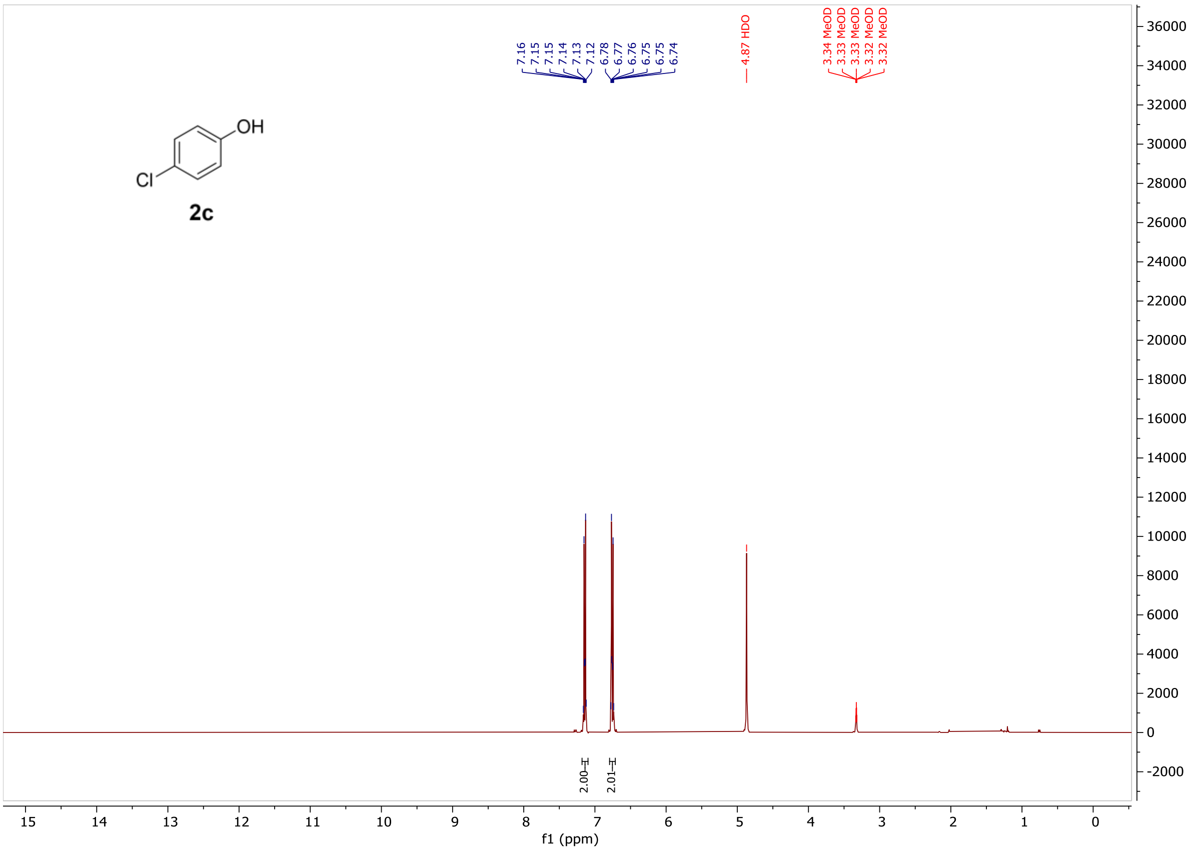

^{13}C-NMR

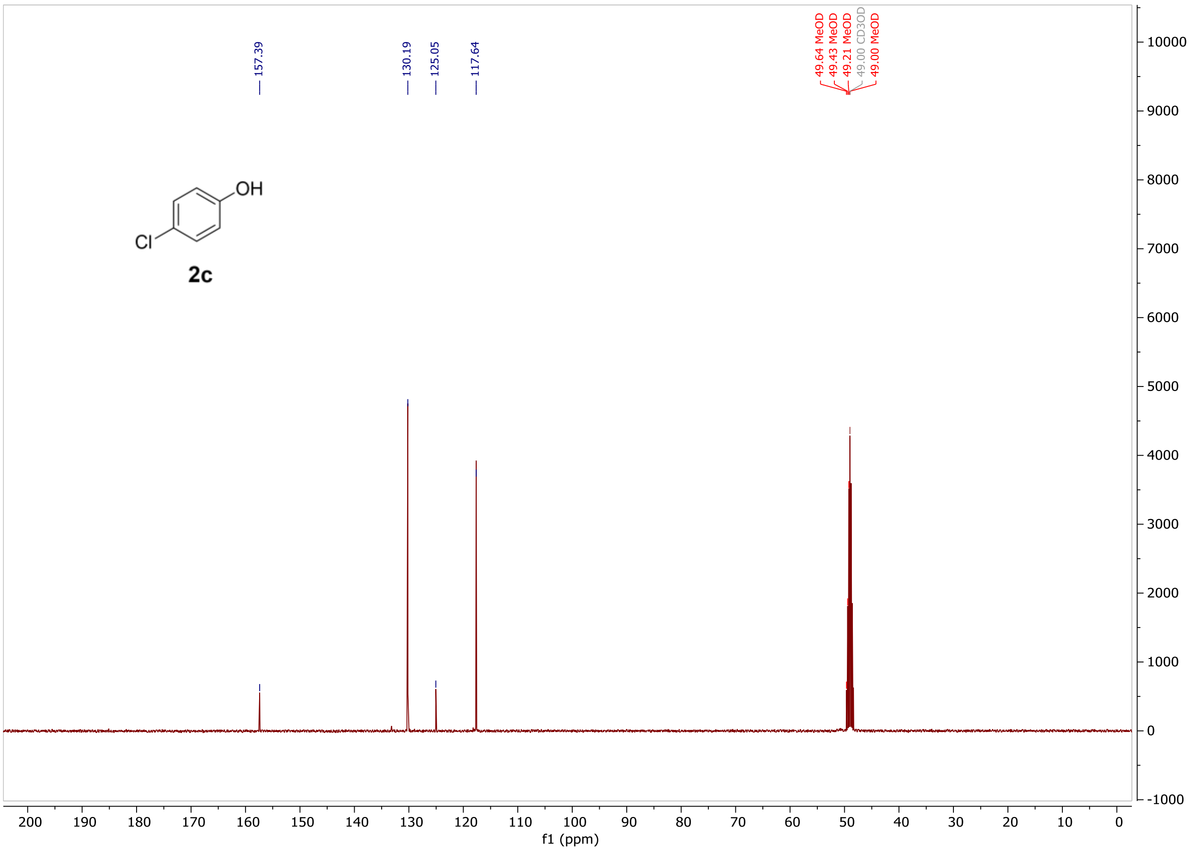

^{1}H-NMR

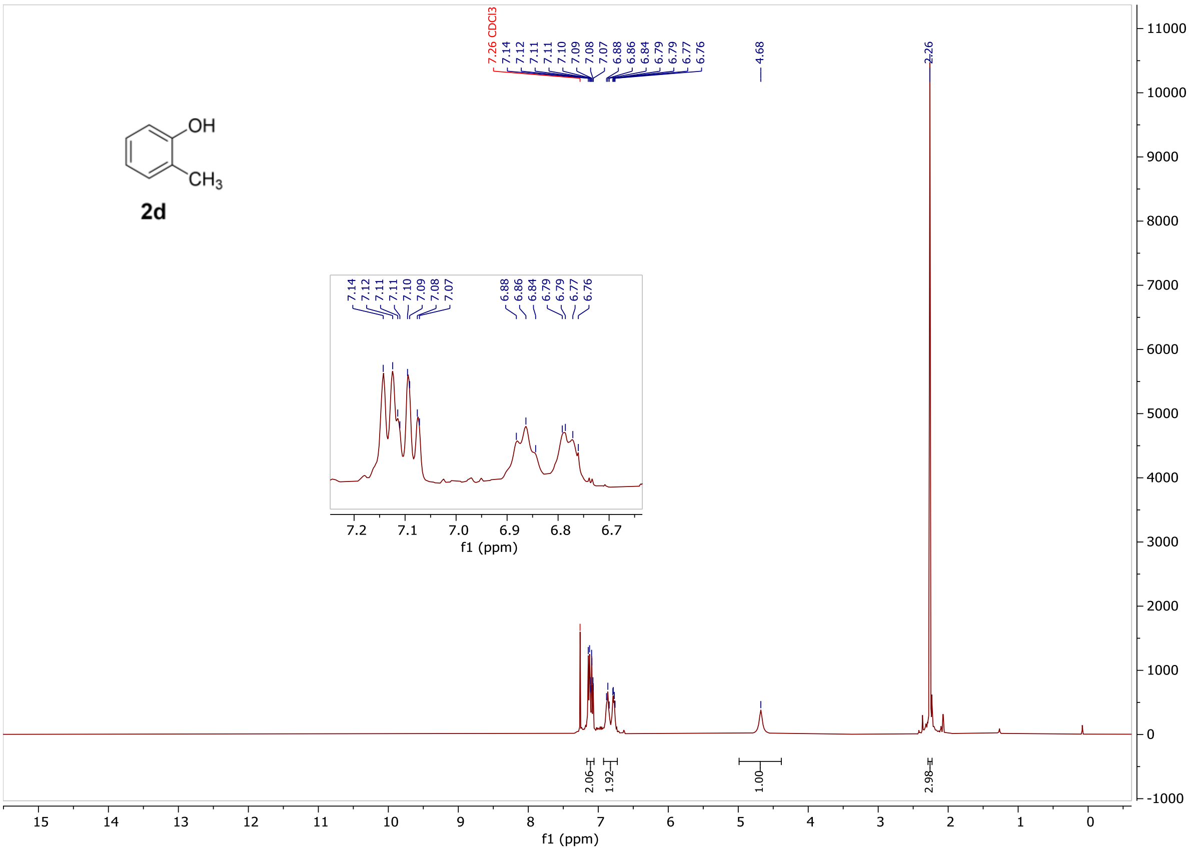

^{13}C-NMR

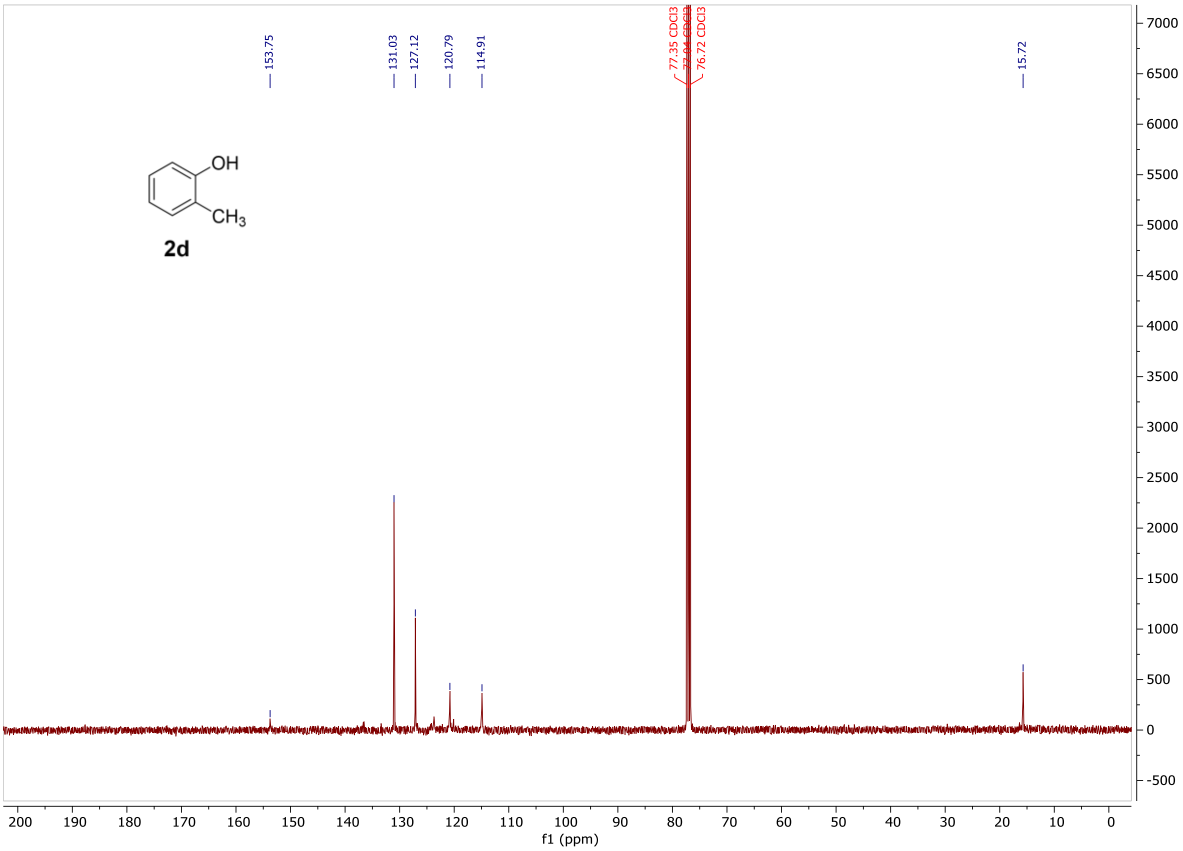

^{1}H-NMR

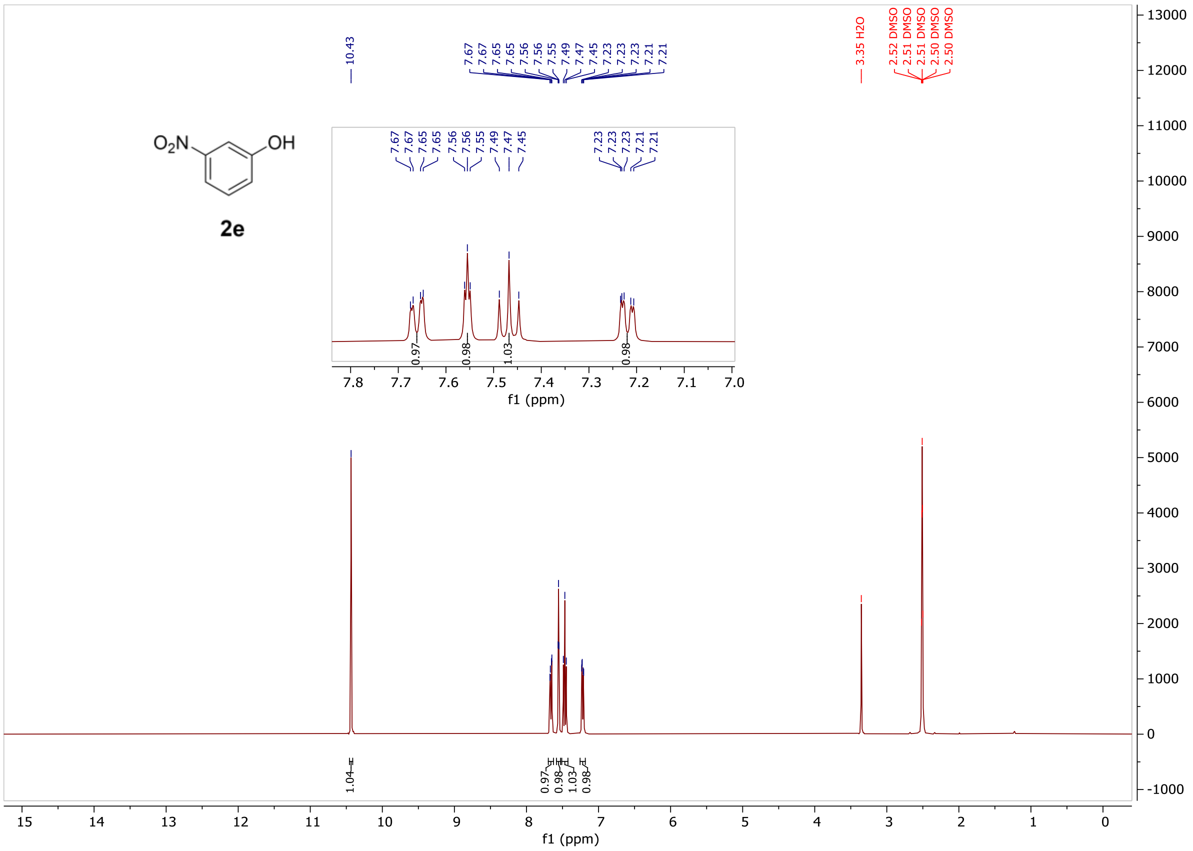

^{13}C-NMR

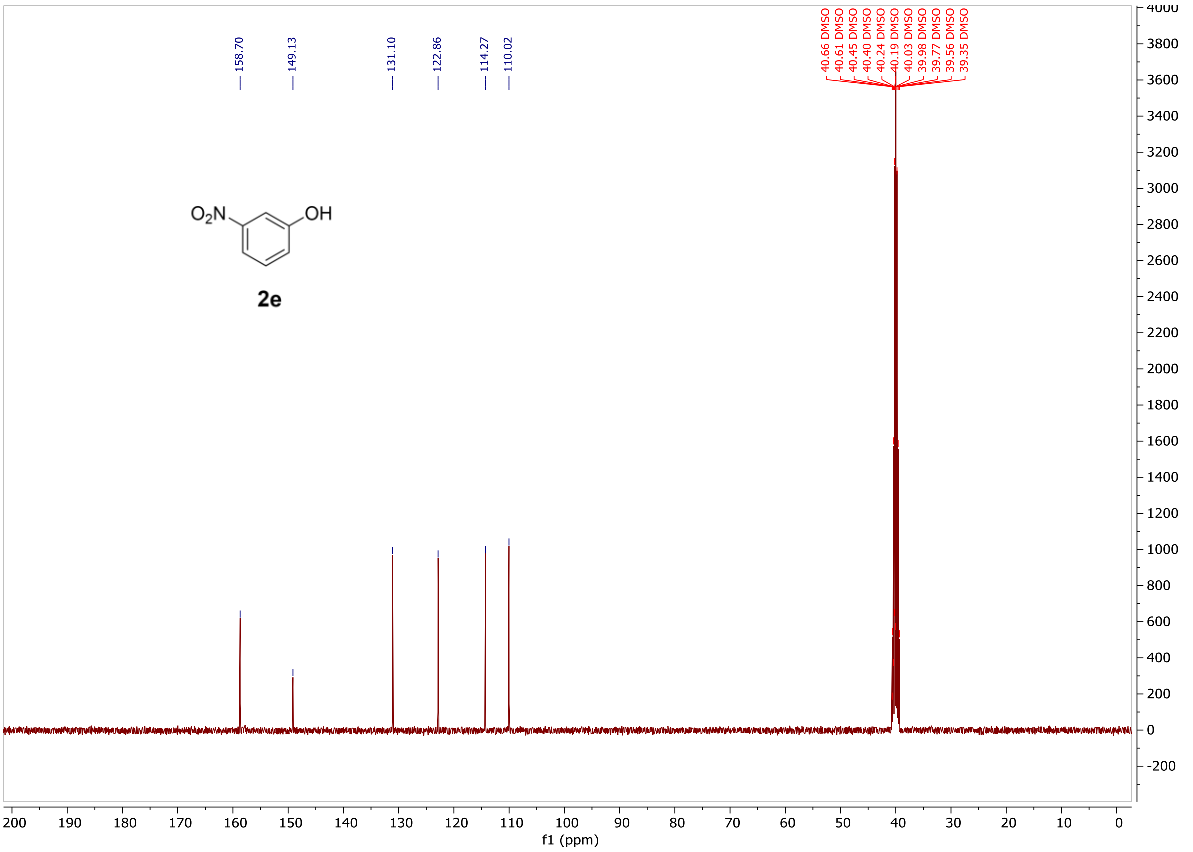

^{1}H-NMR

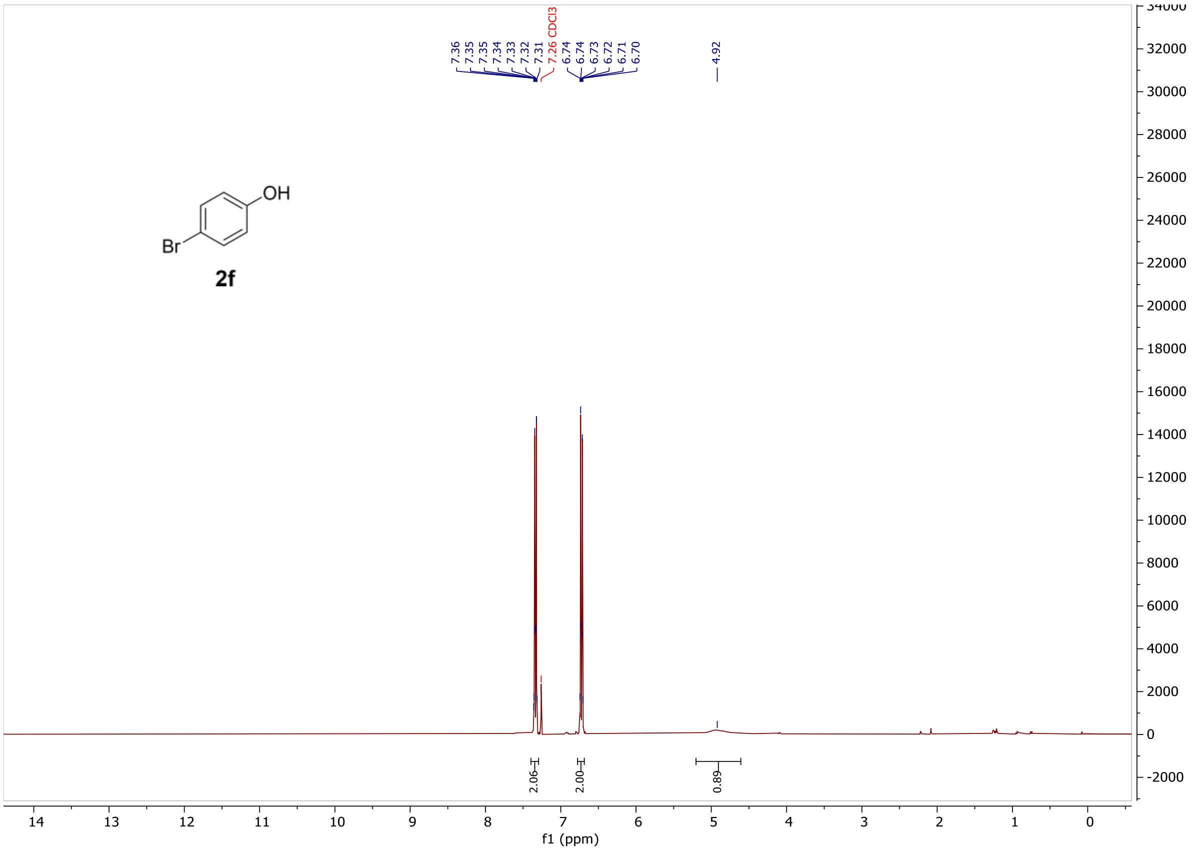

^{13}C-NMR

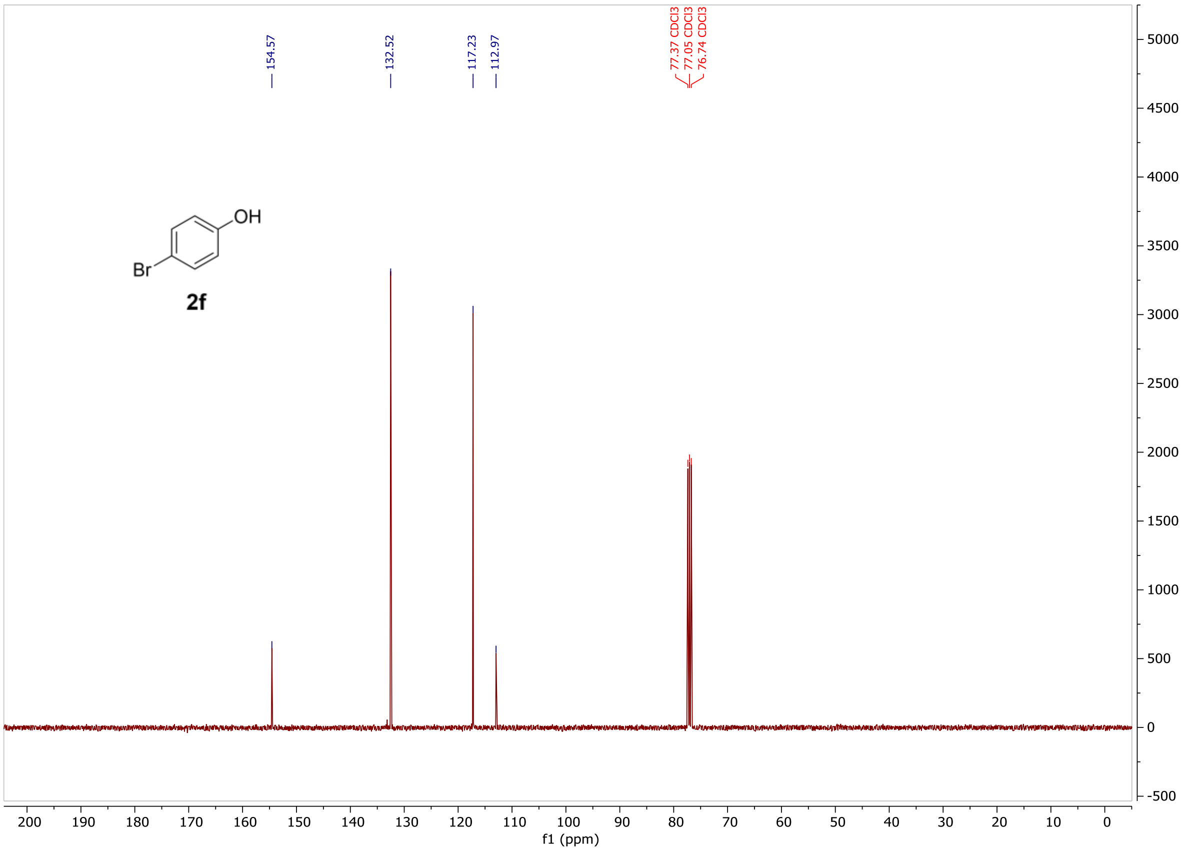

^{1}H-NMR

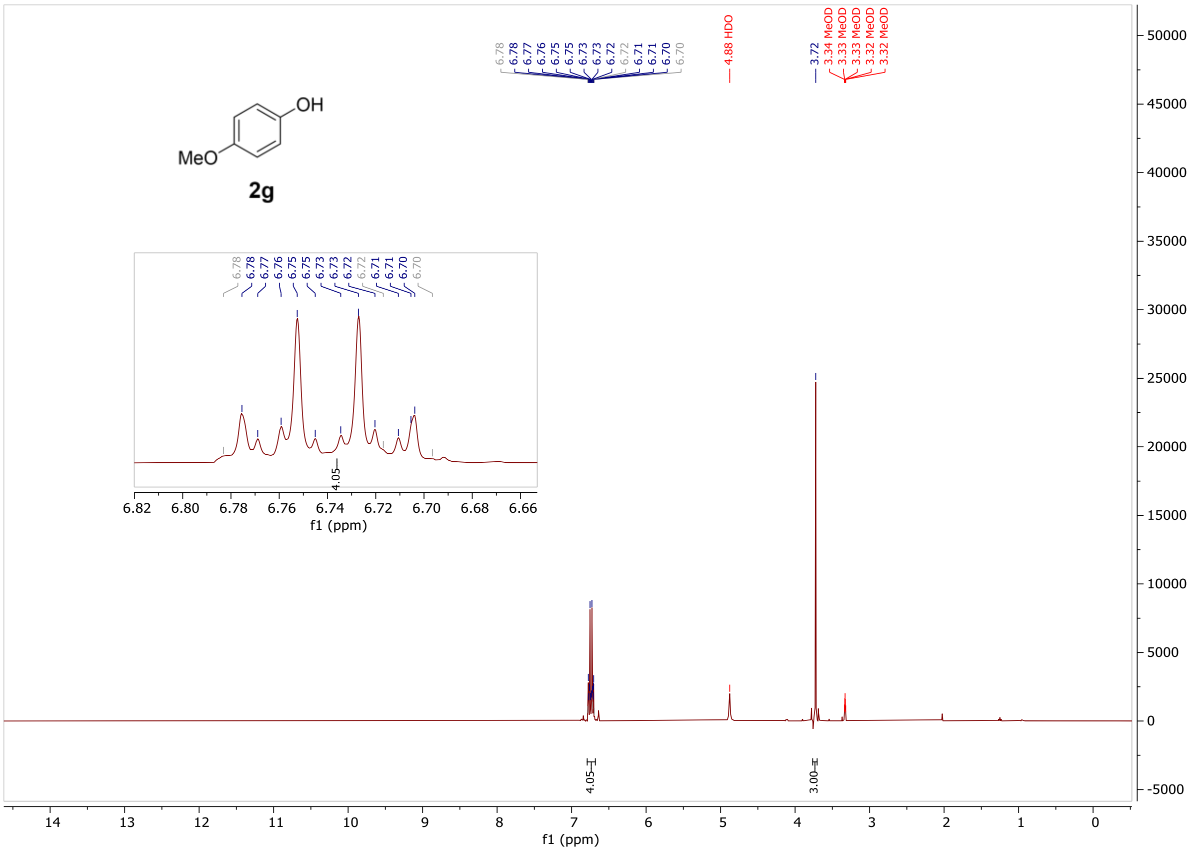

^{13}C-NMR

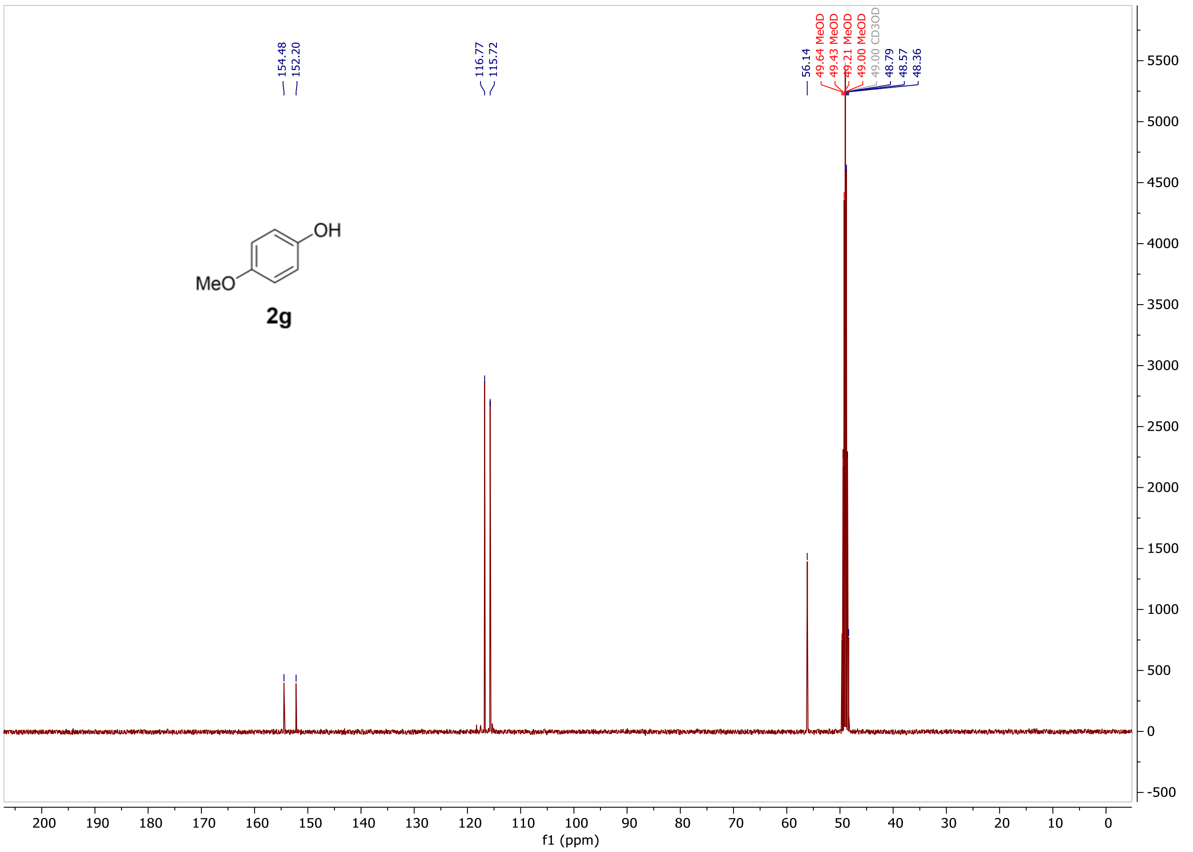

^{1}H-NMR

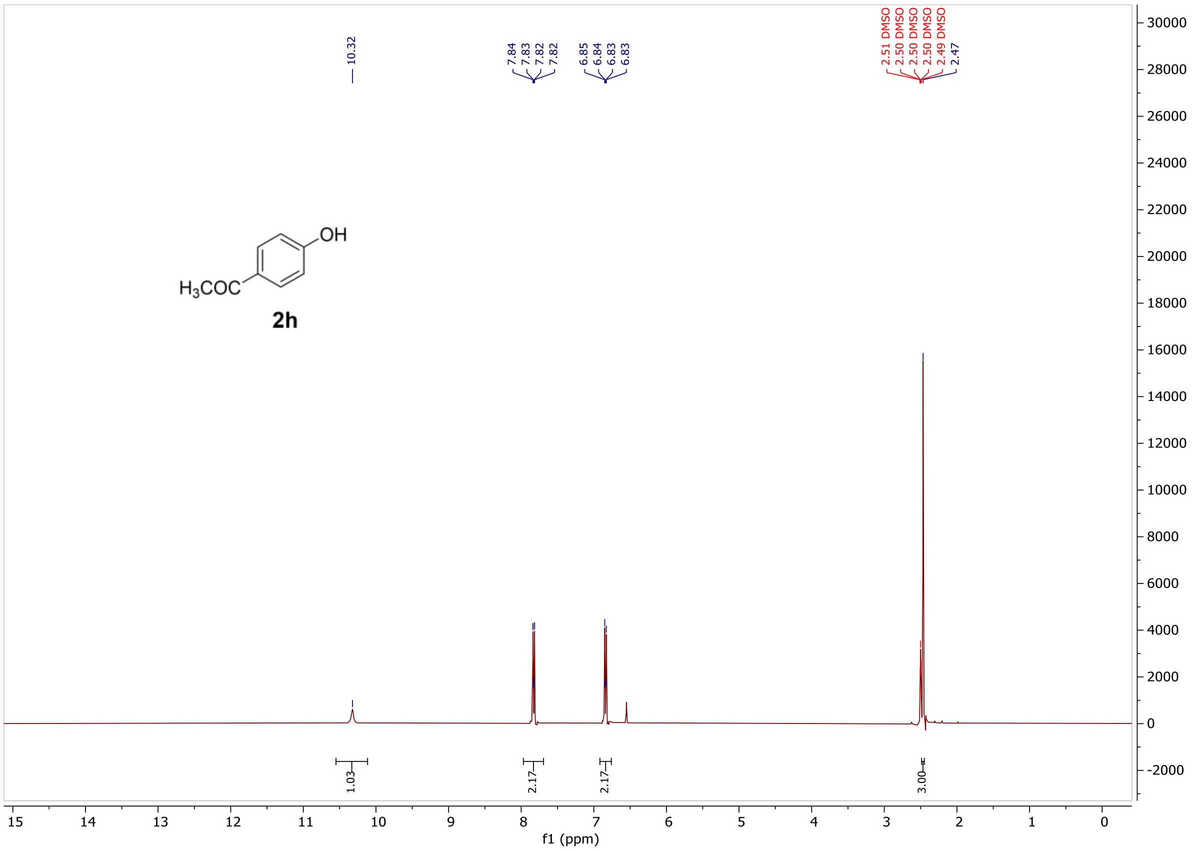

^{13}C-NMR

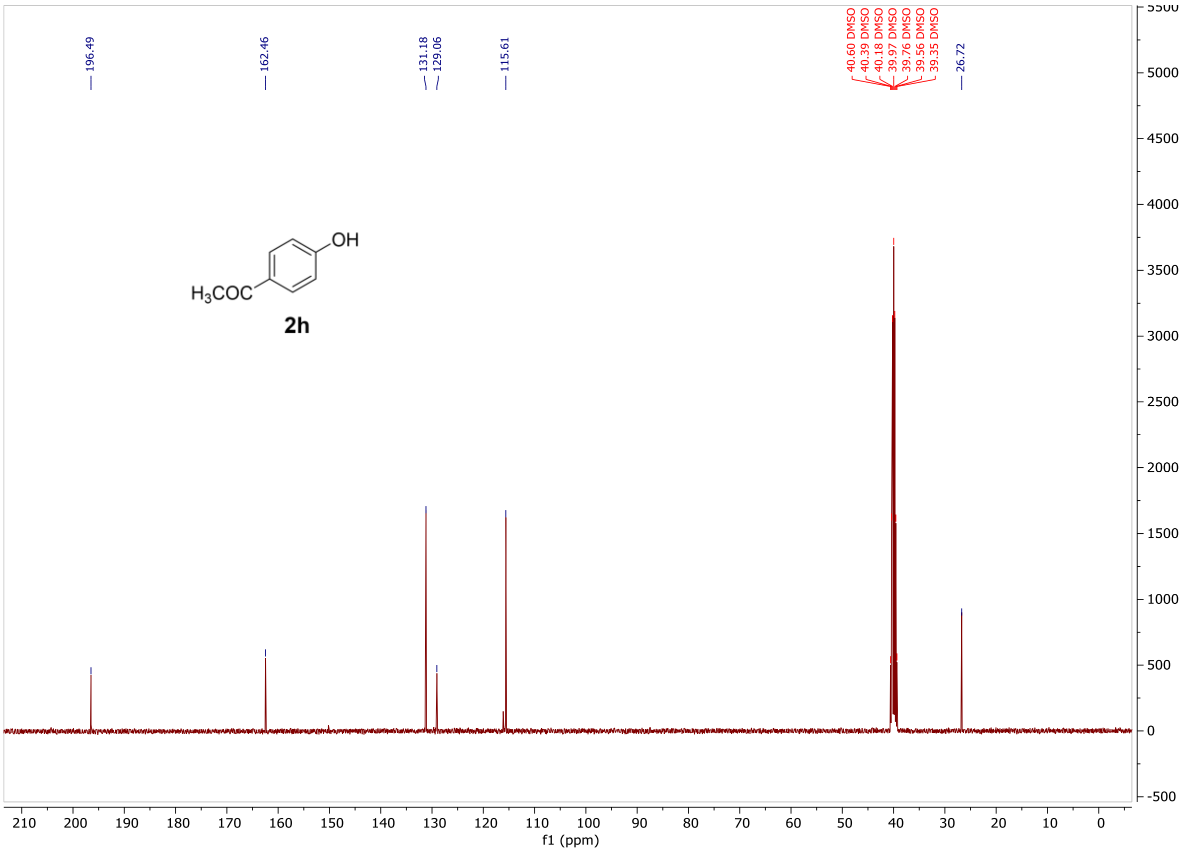

^{1}H-NMR

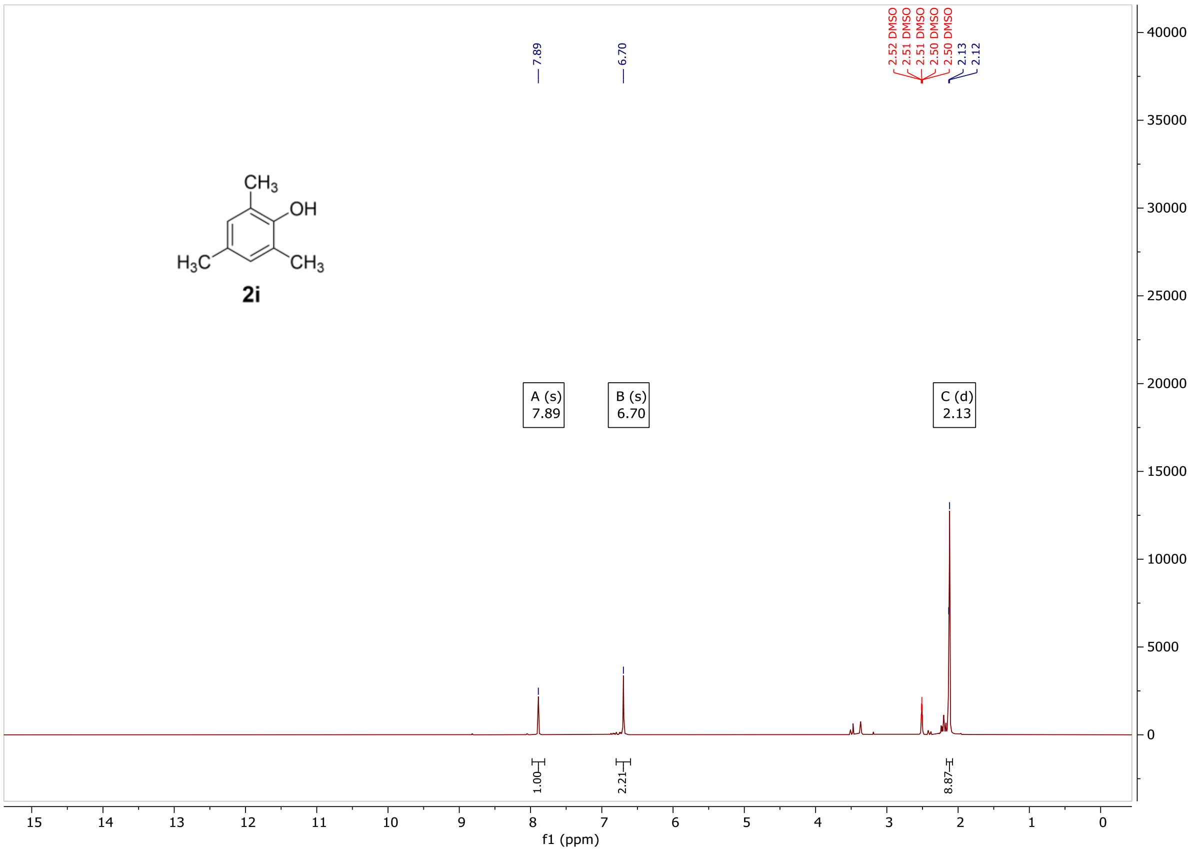

^{13}C-NMR

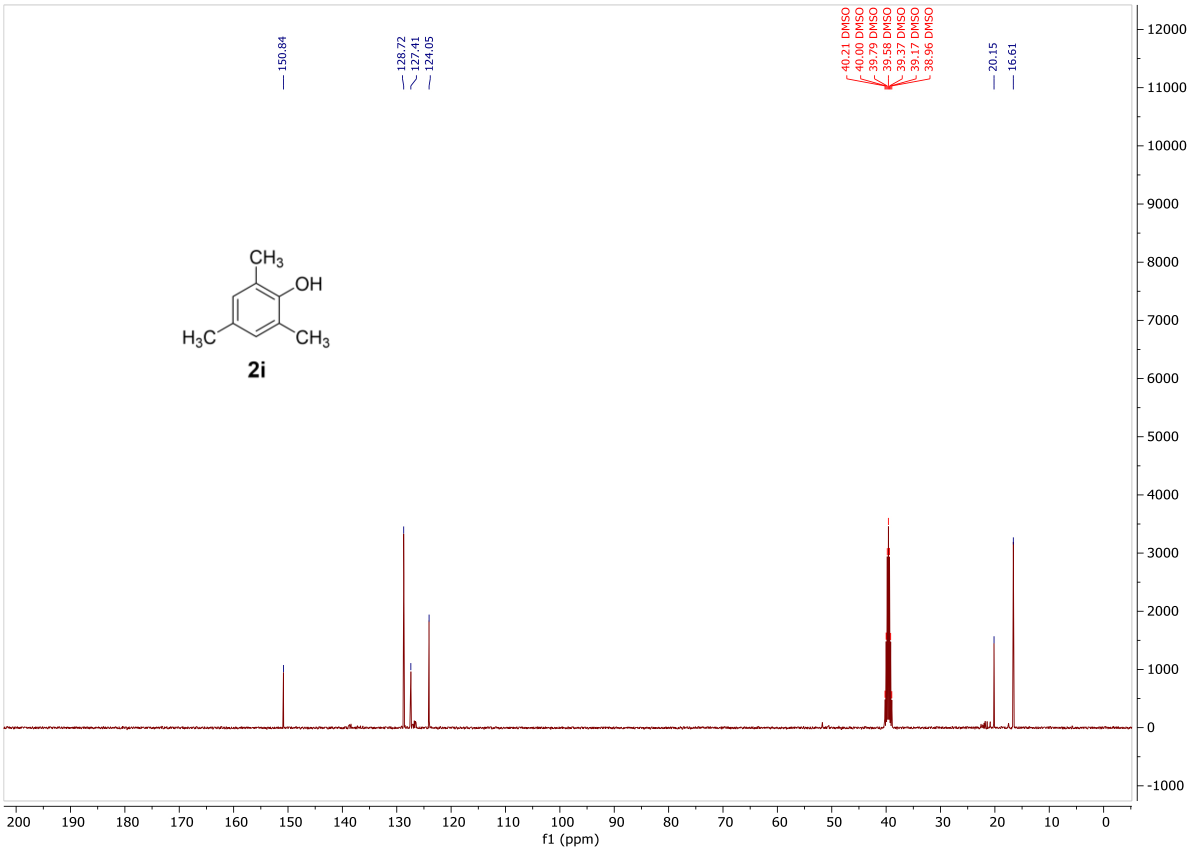

^{1}H-NMR

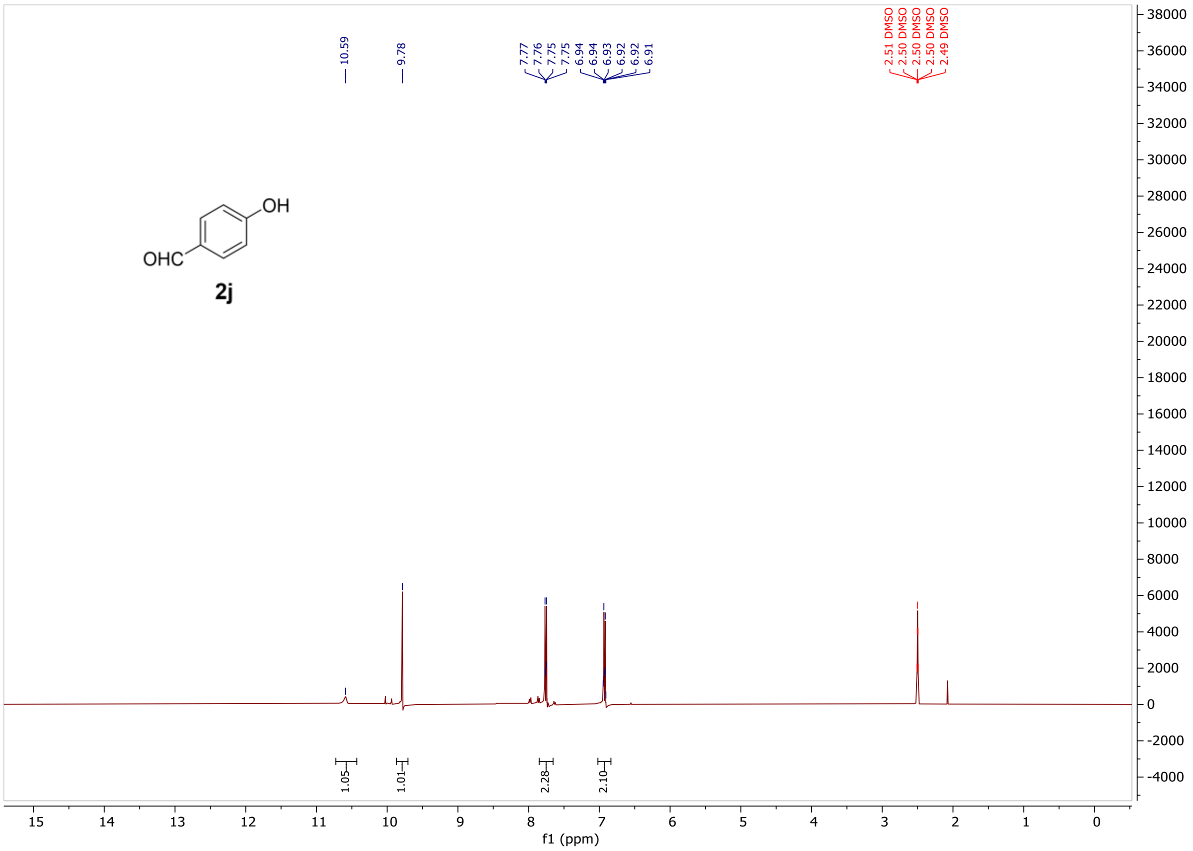

^{13}C-NMR

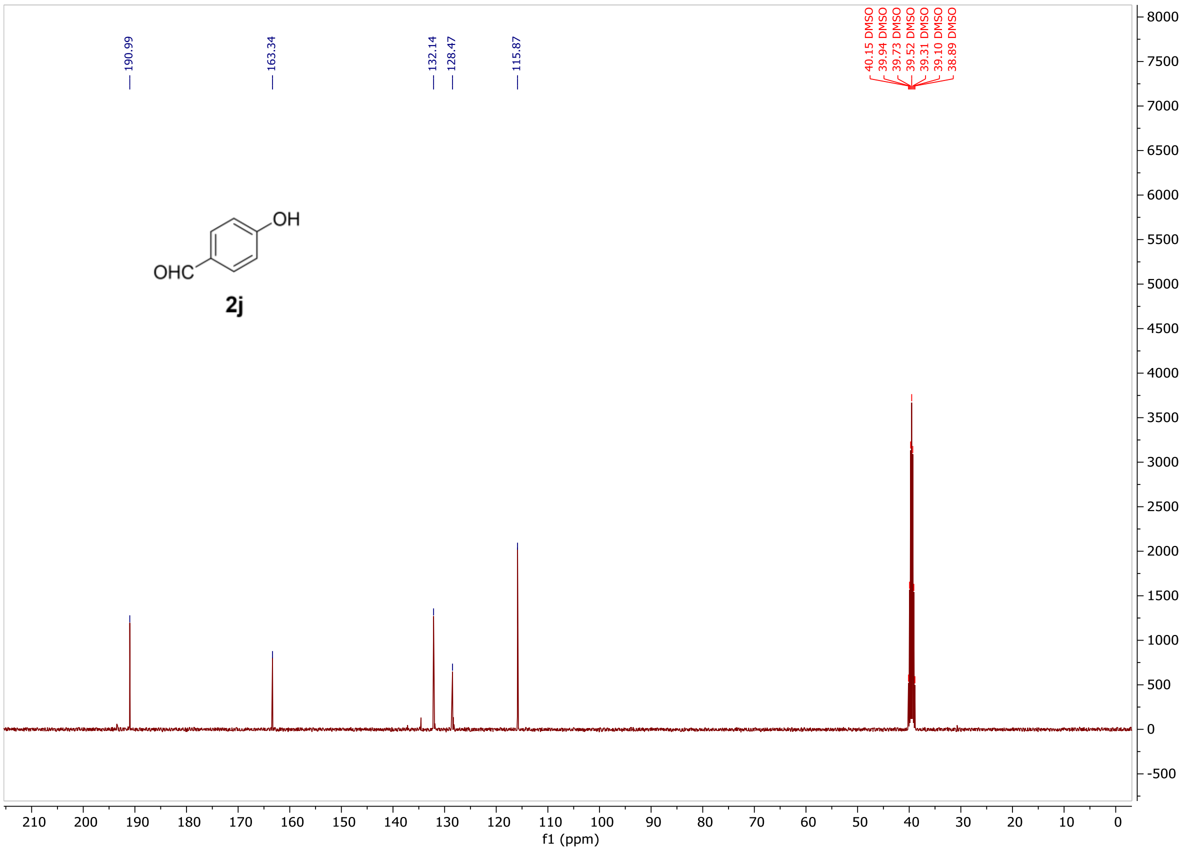

^{1}H-NMR

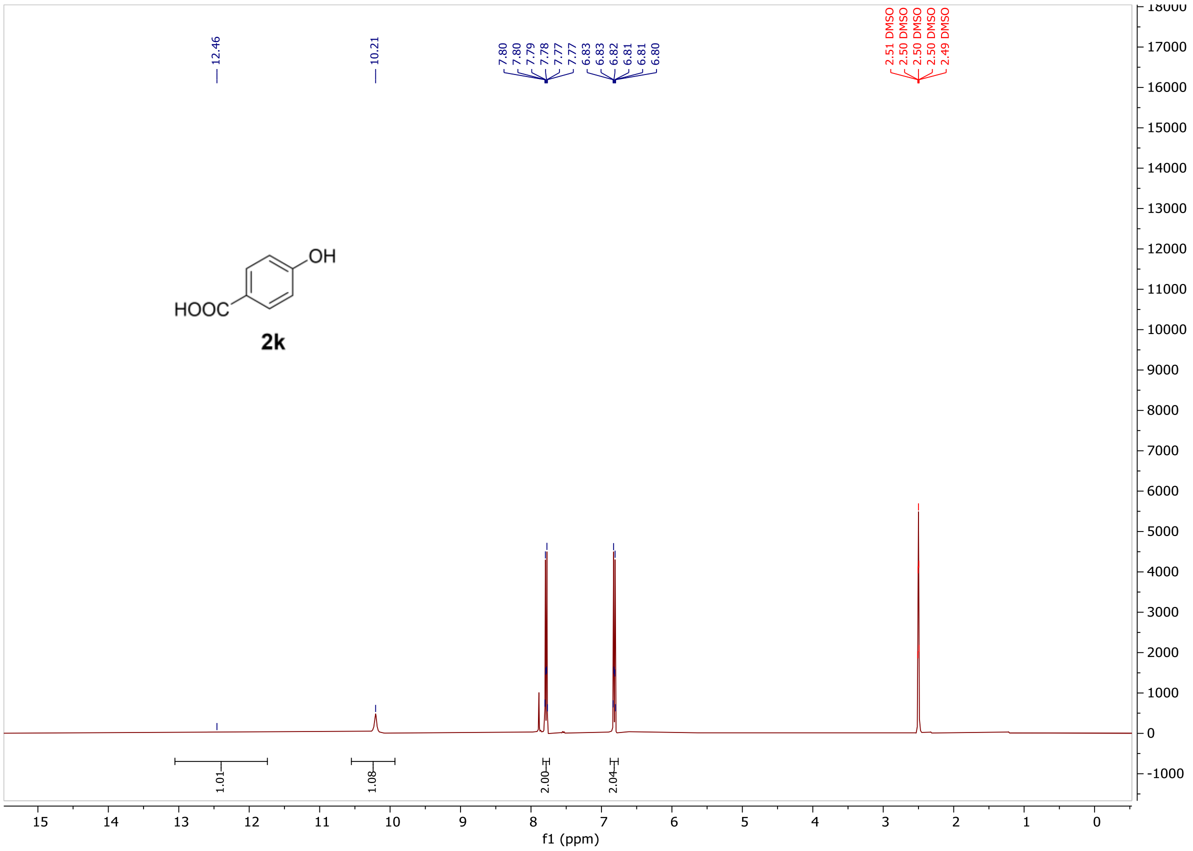

^{13}C-NMR

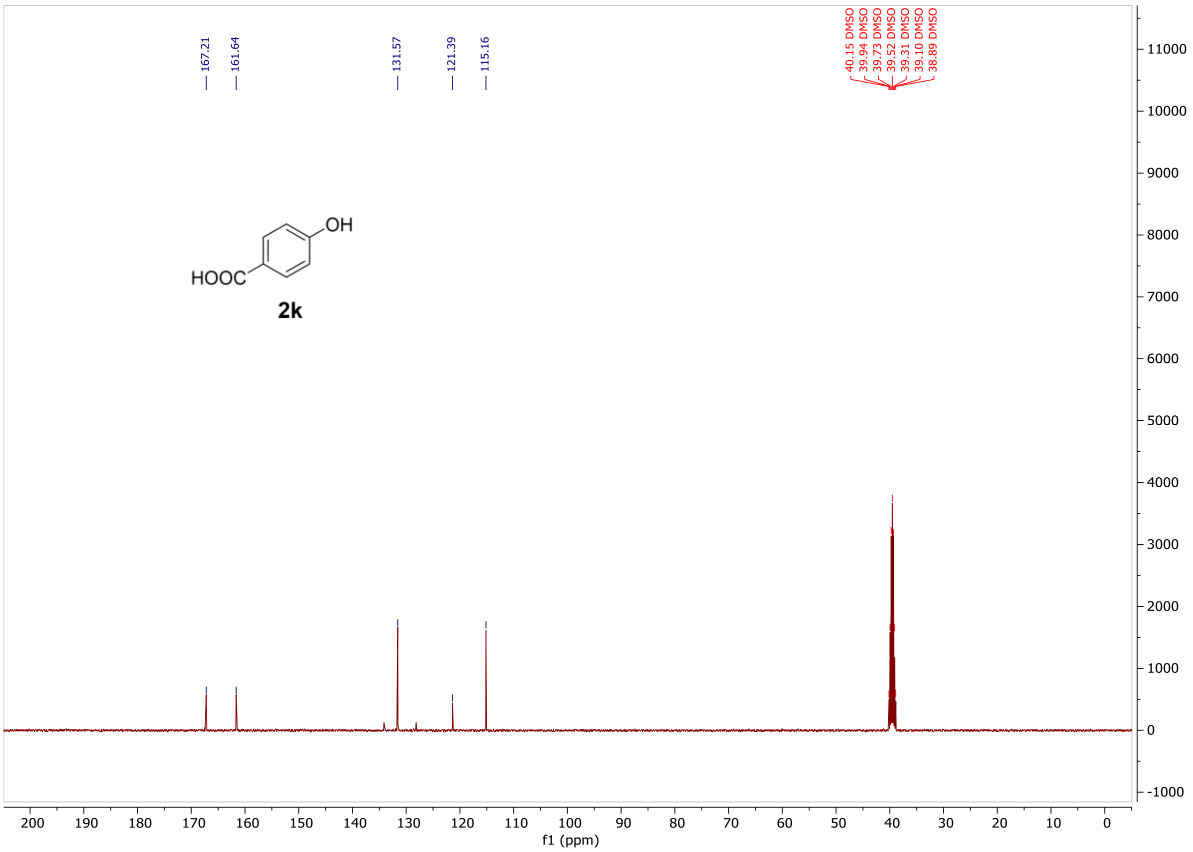

^{1}H-NMR

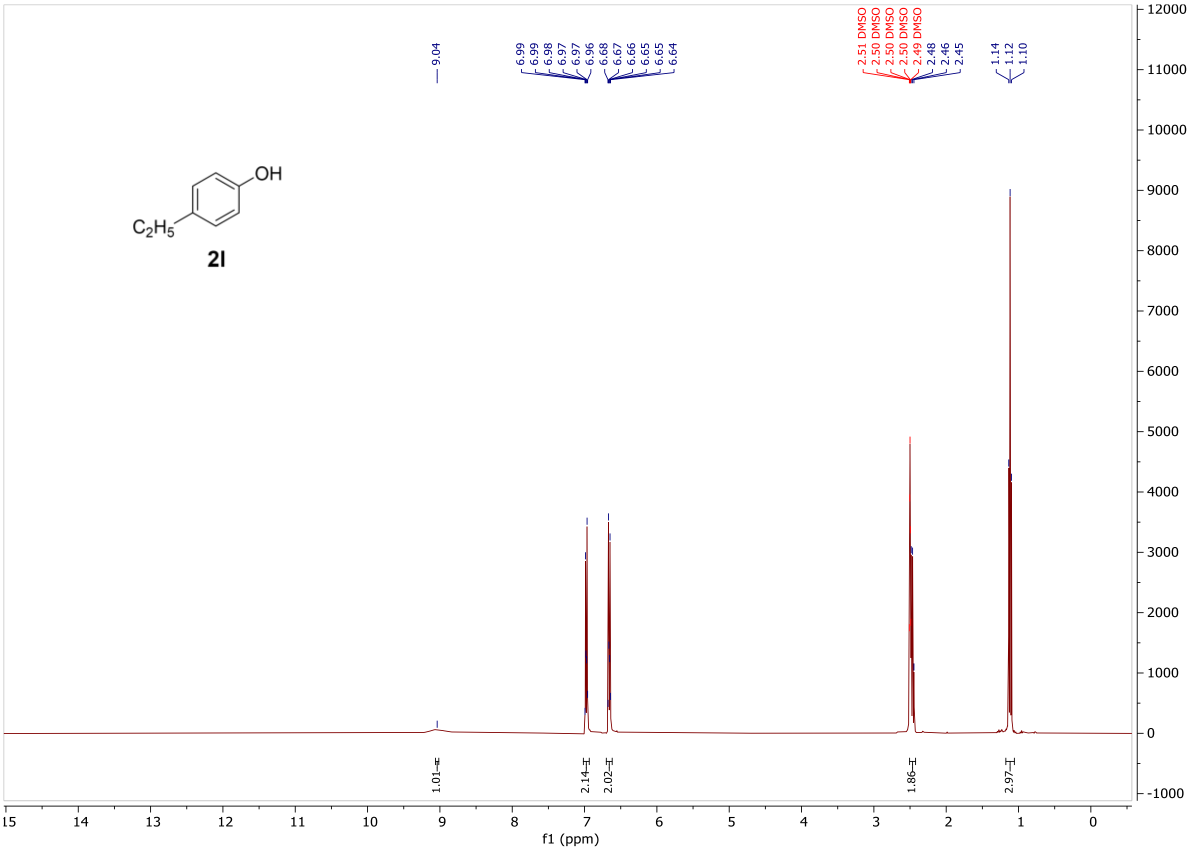

^{13}C-NMR

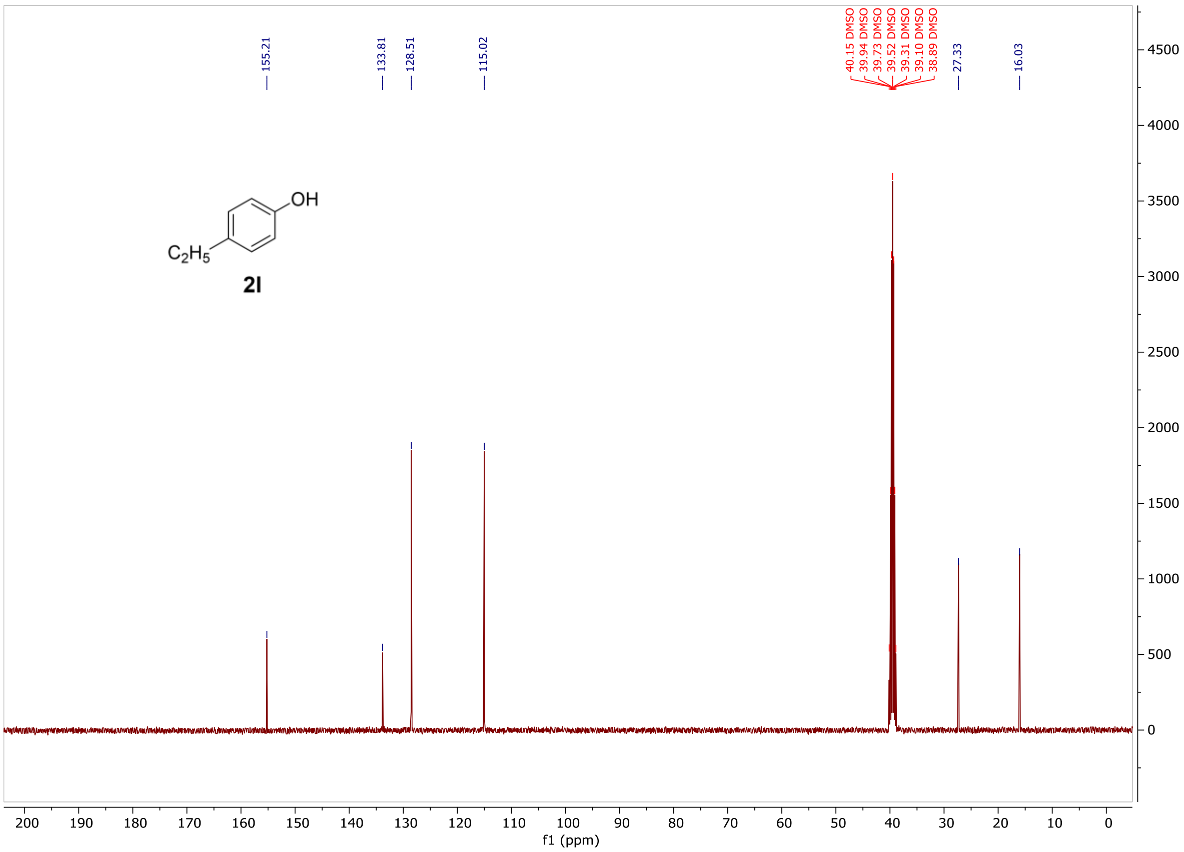

¹H-NMR

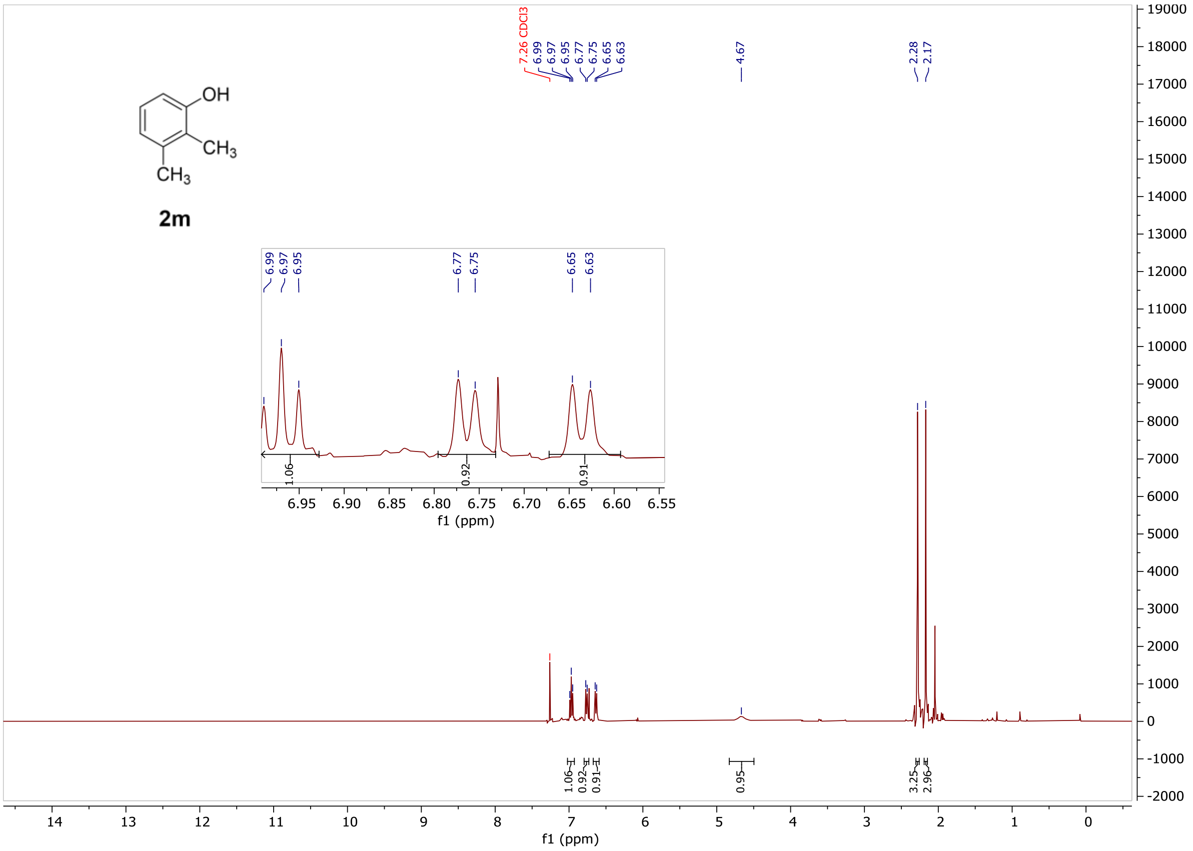

¹³C-NMR

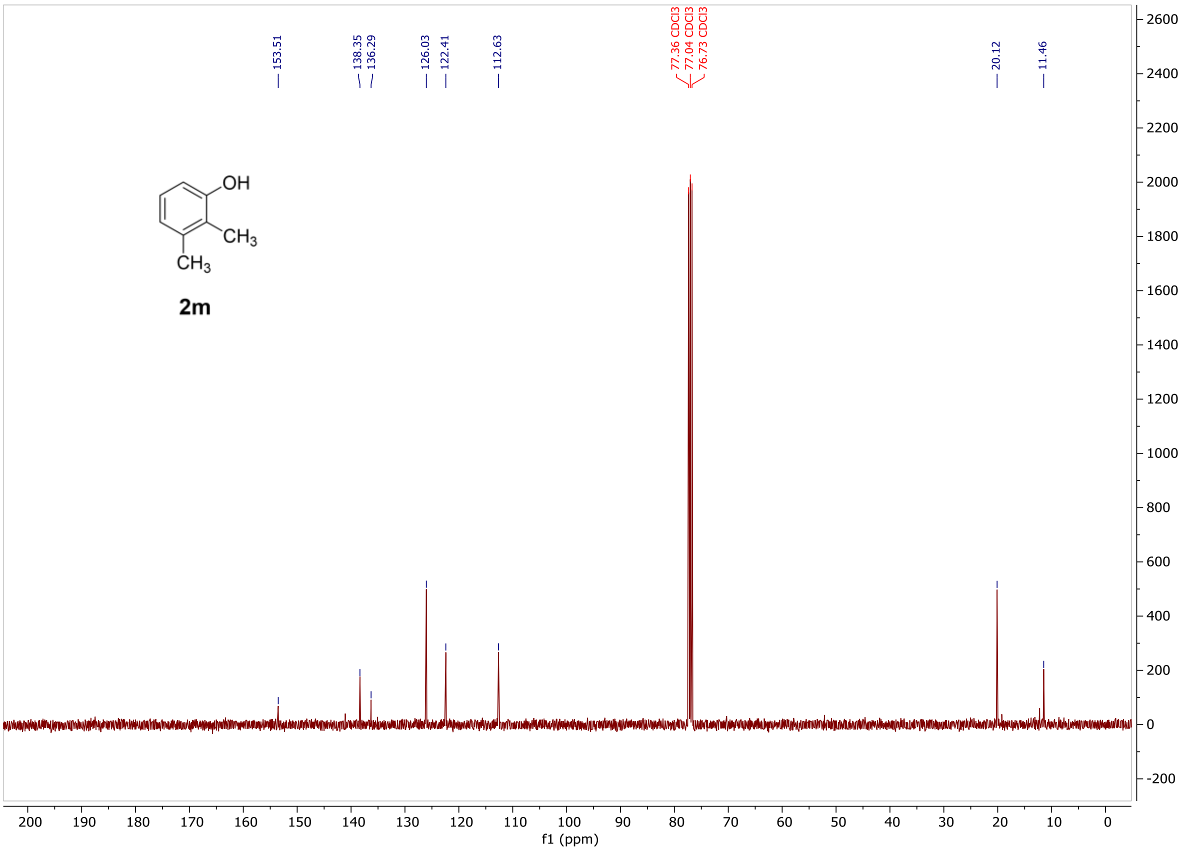

^{1}H-NMR

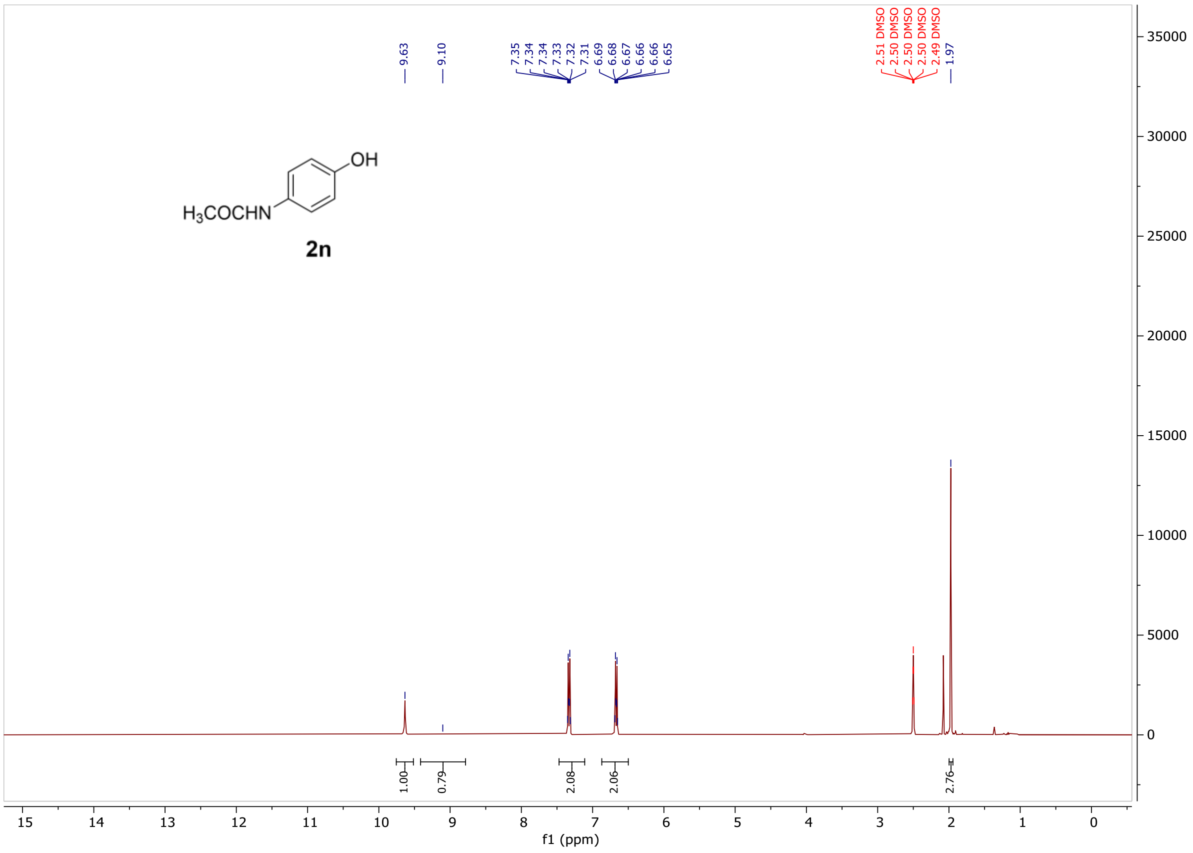

^{13}C-NMR

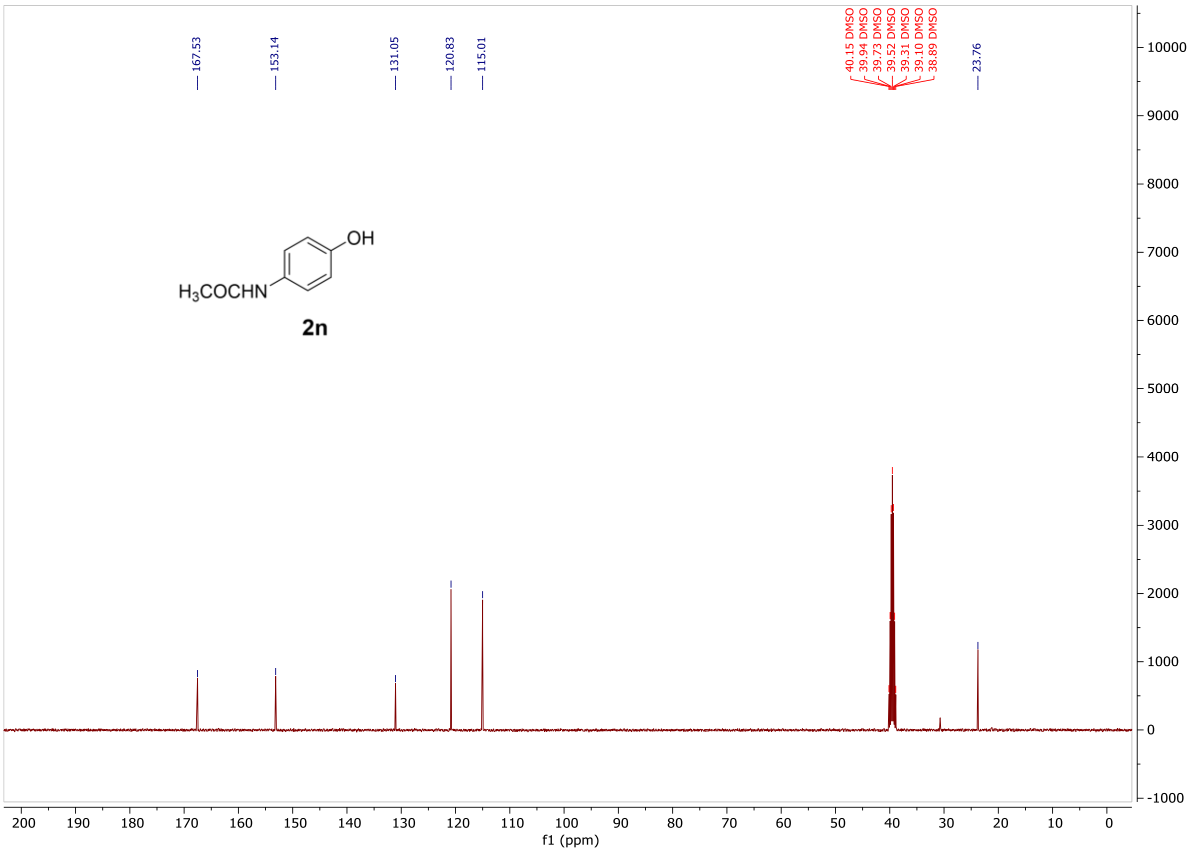

^{1}H-NMR

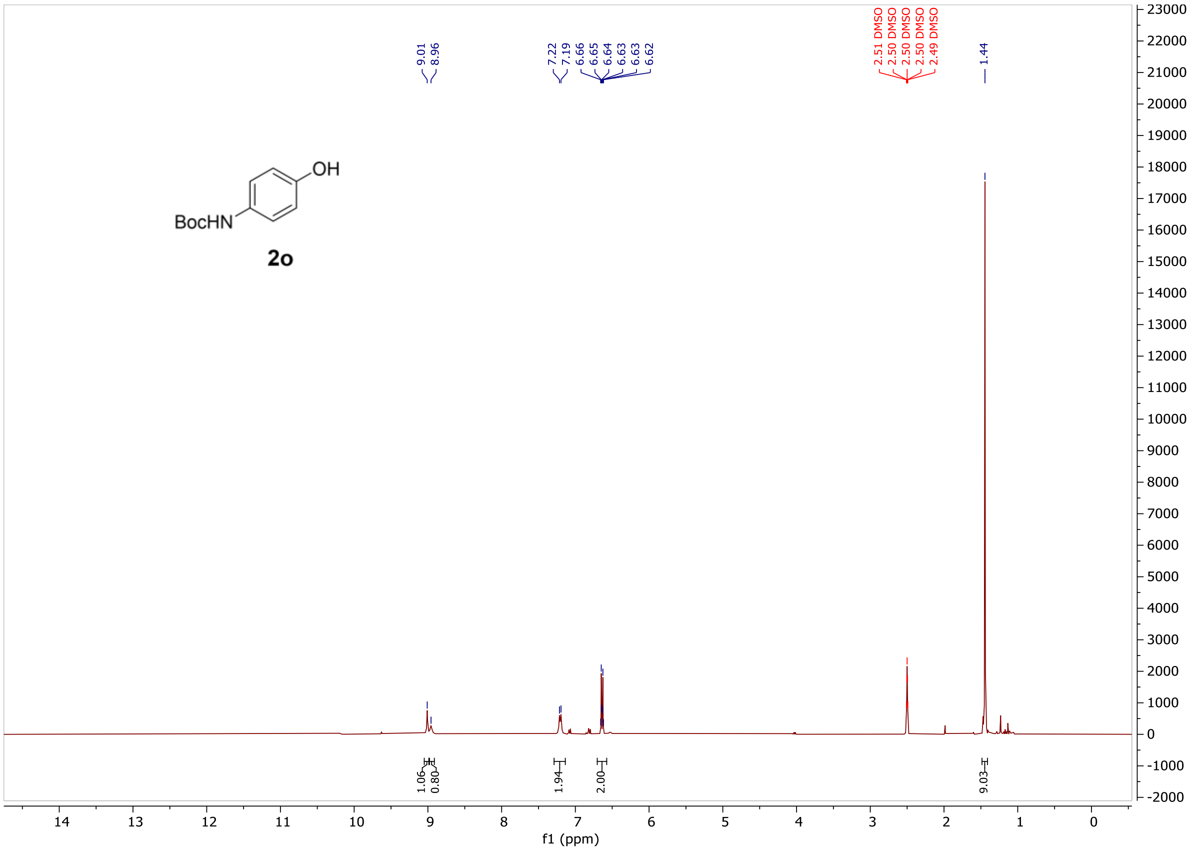

^{13}C-NMR

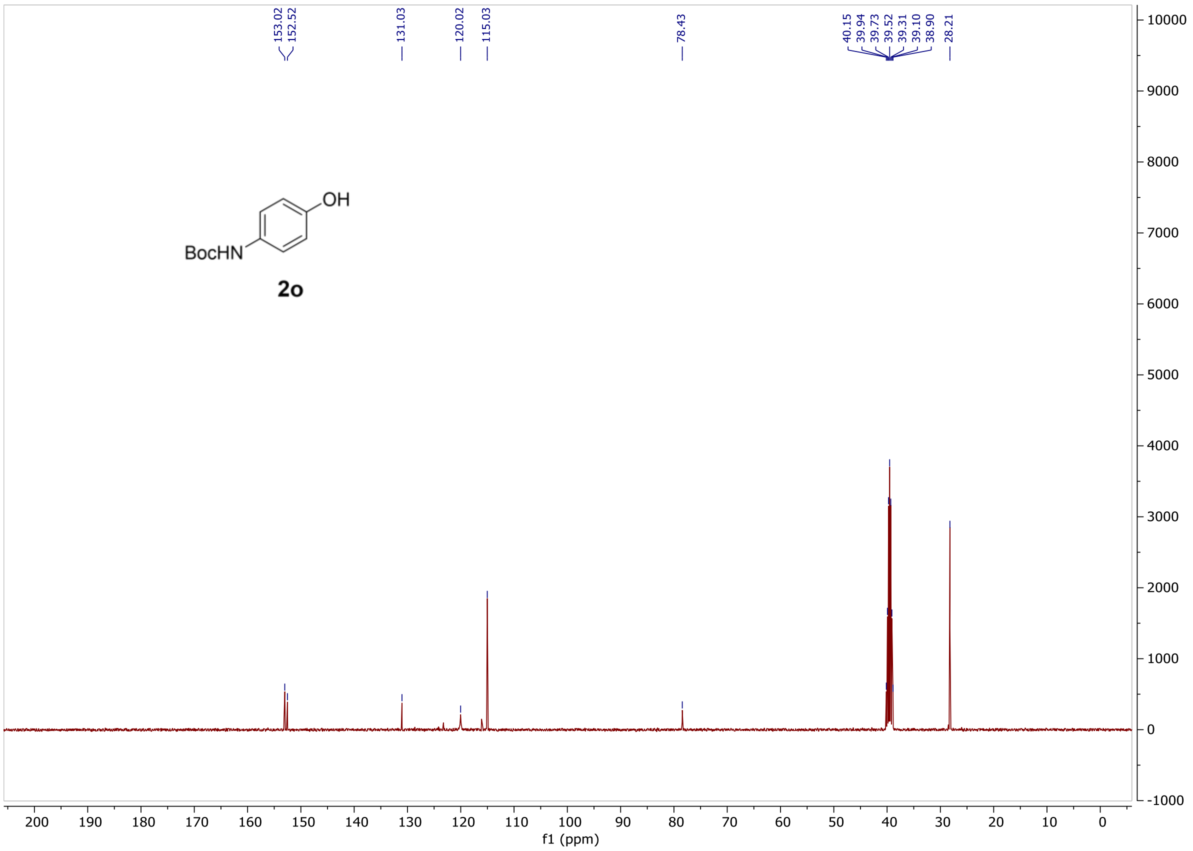

^{1}H-NMR

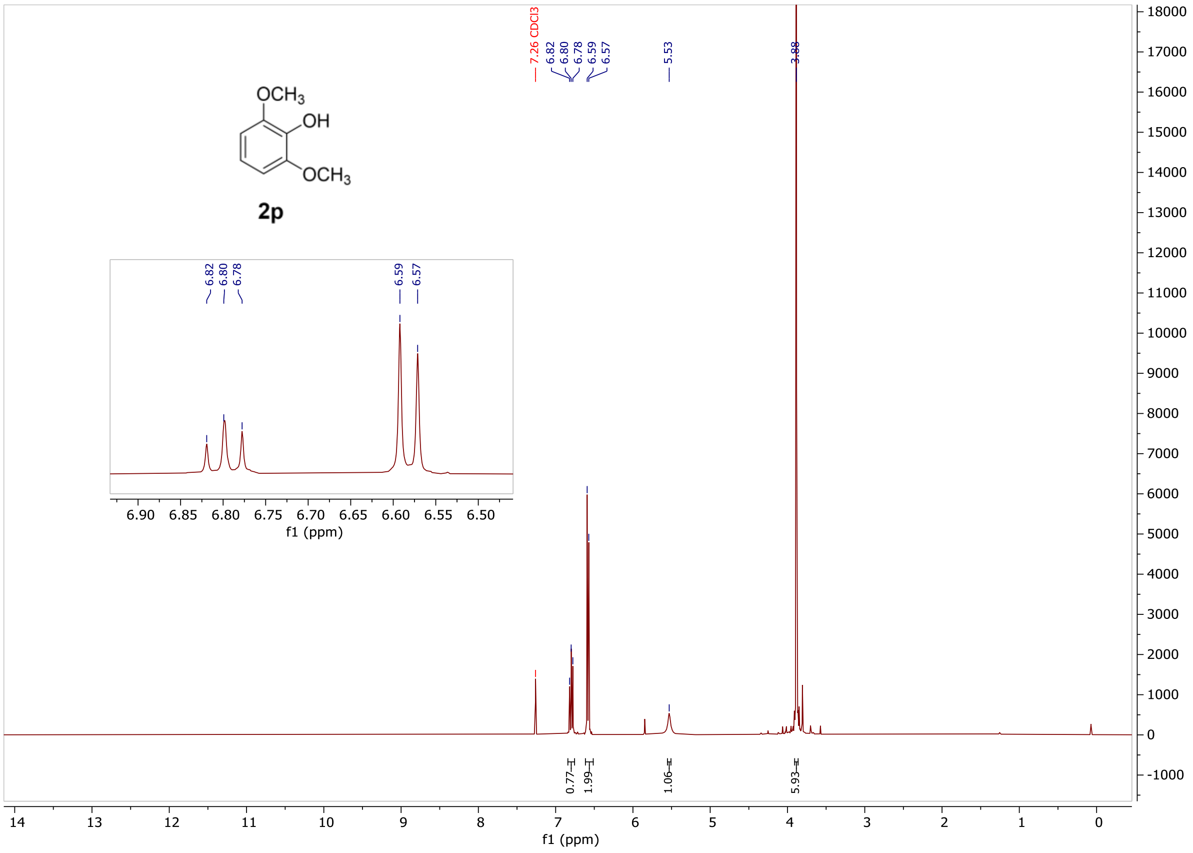

^{13}C-NMR

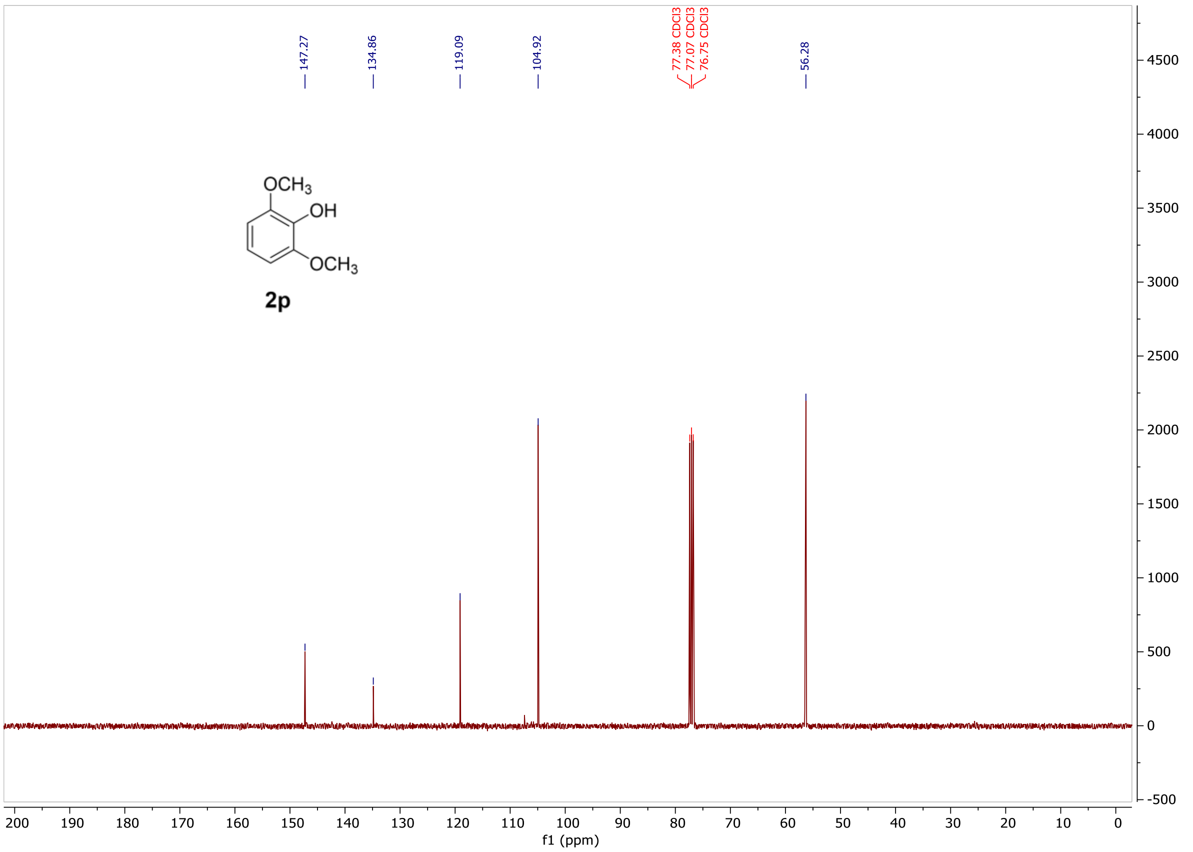

^{1}H-NMR

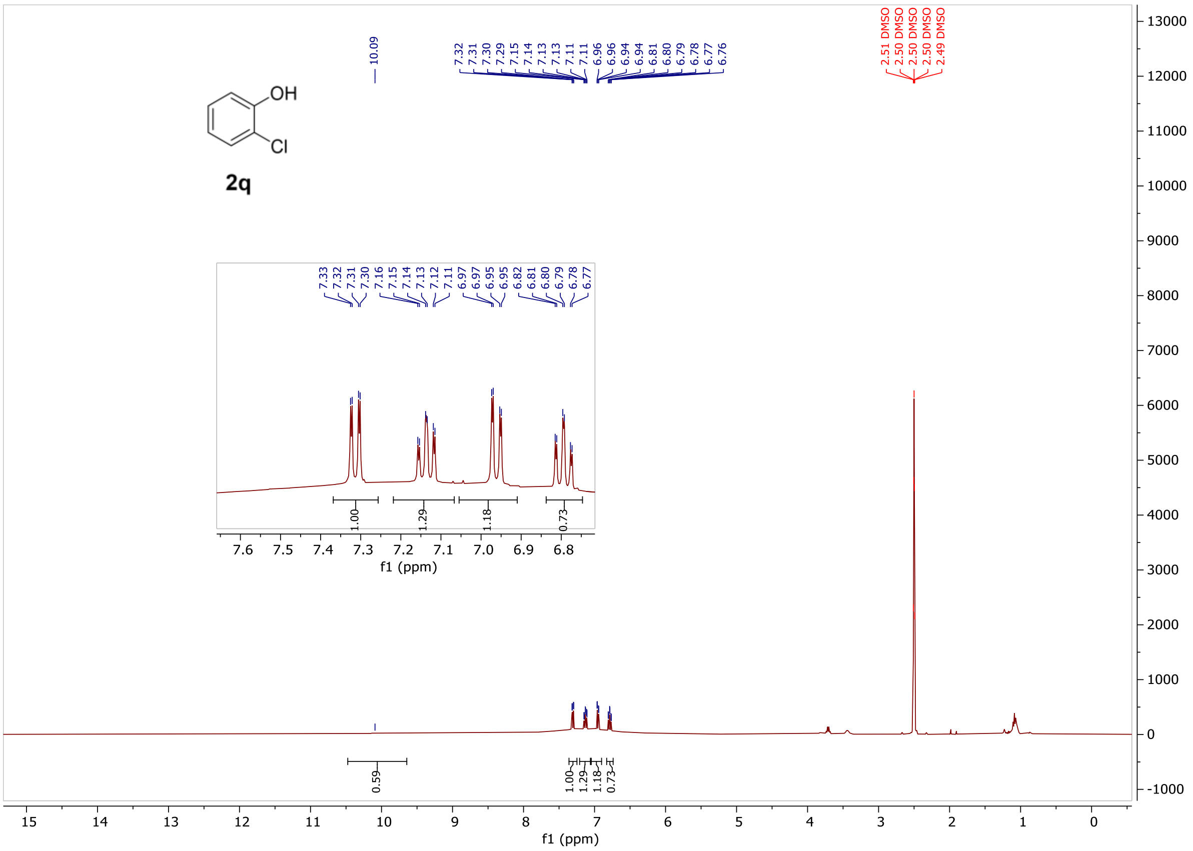

^{13}C-NMR

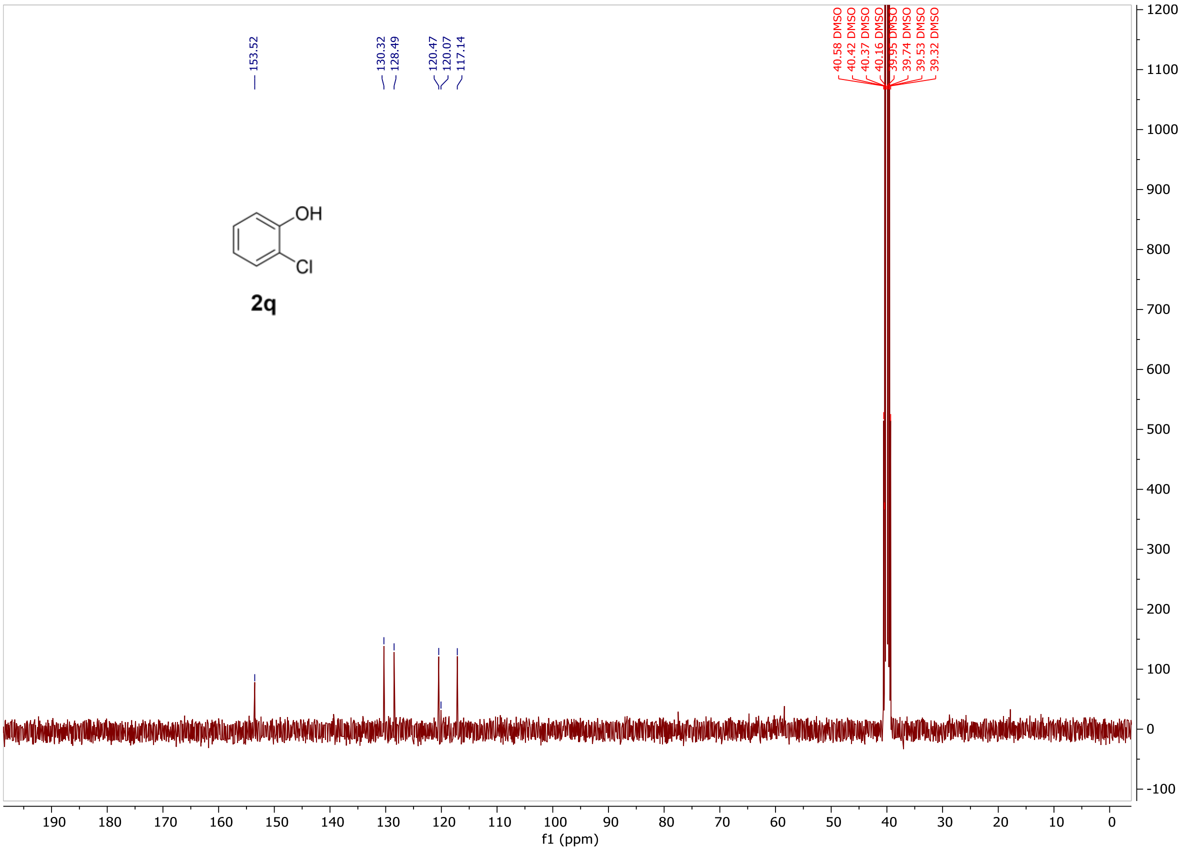

^{1}H-NMR

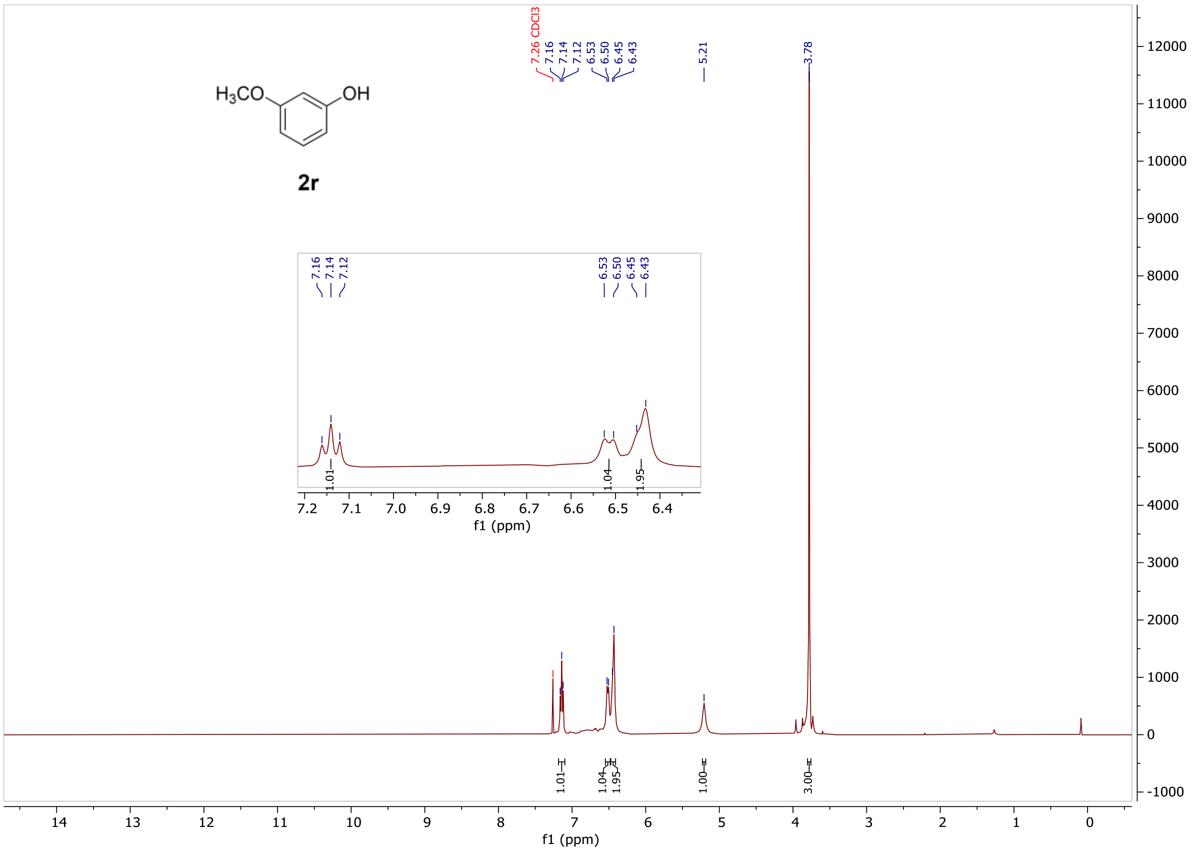

^{13}C-NMR

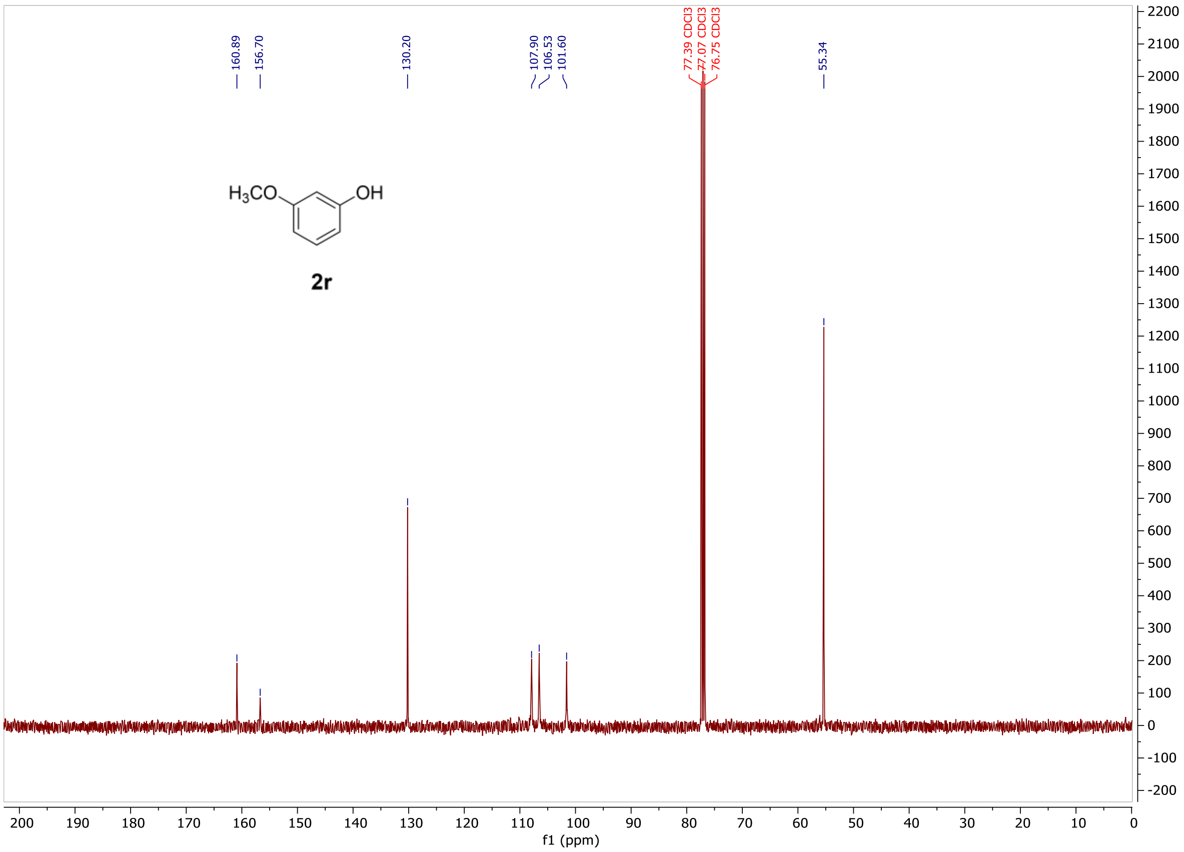

^{1}H-NMR

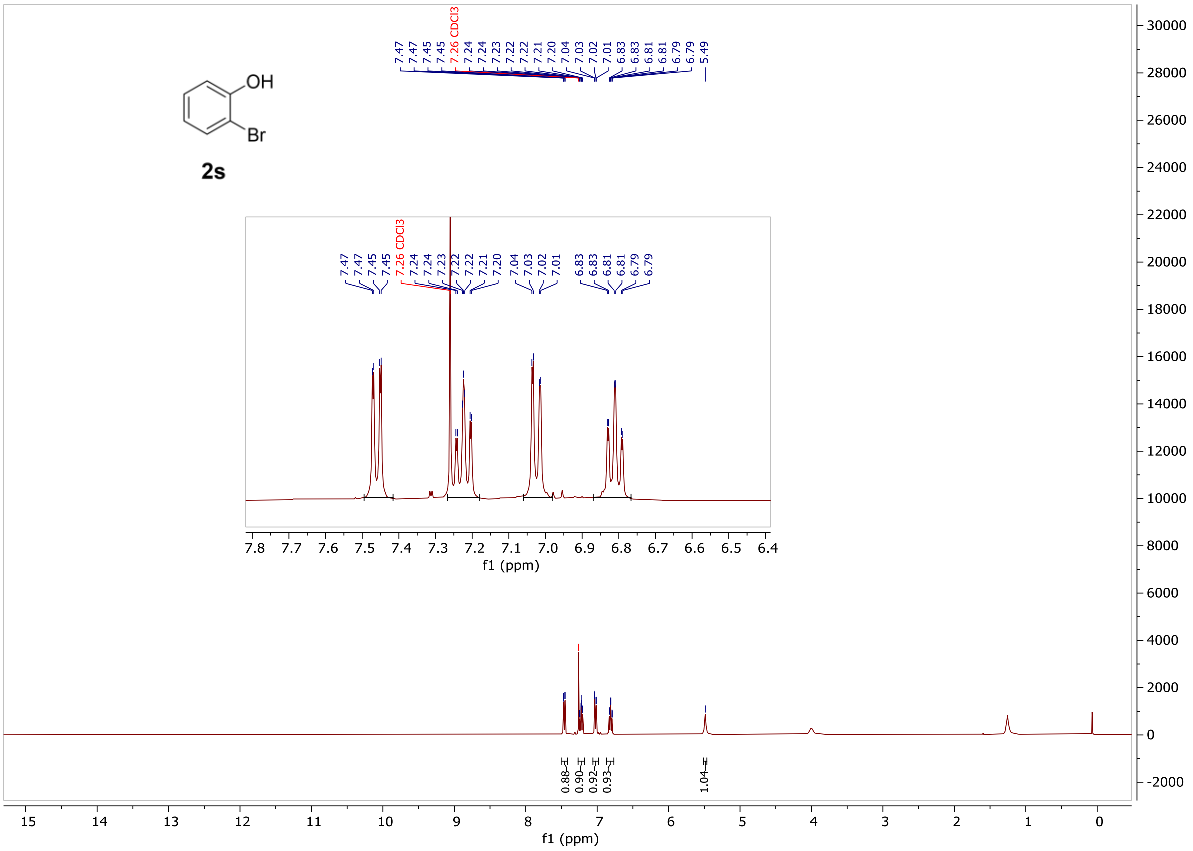

^{13}C-NMR

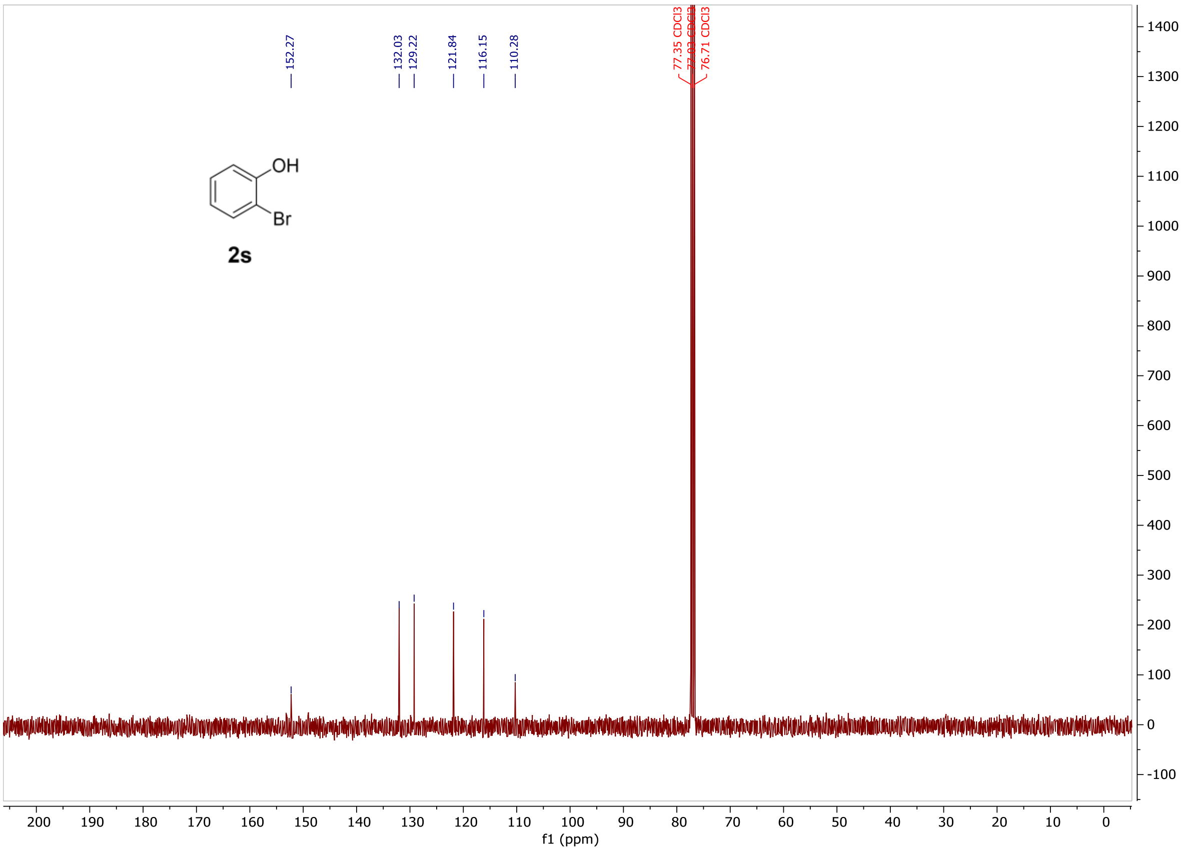

^{1}H-NMR

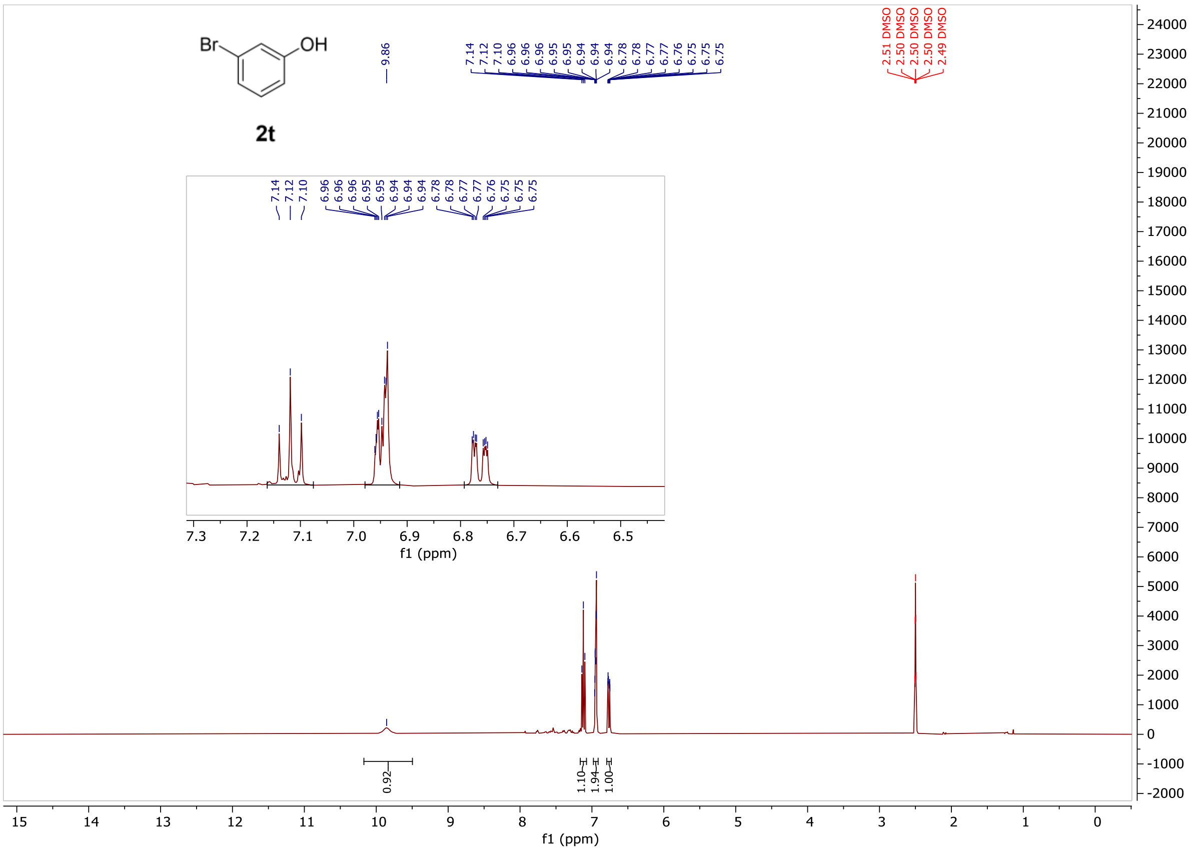

^{13}C-NMR

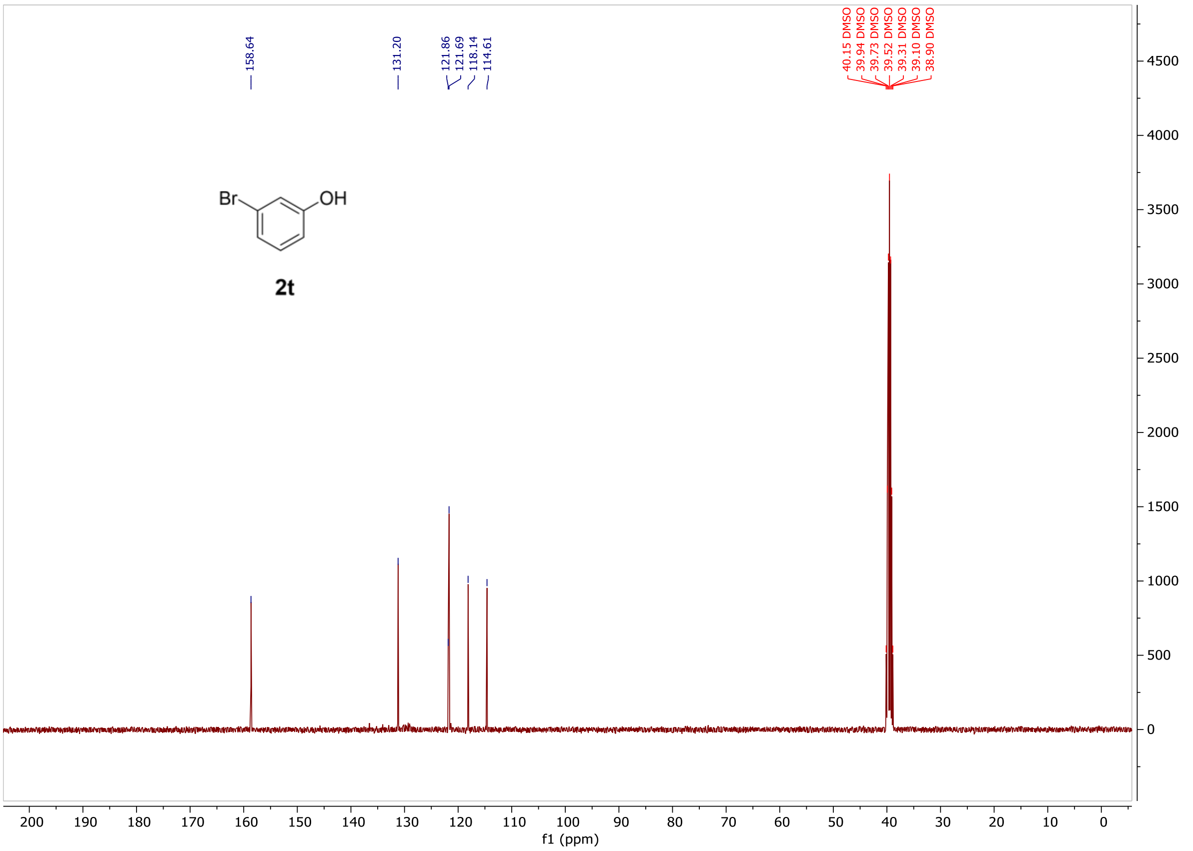

^{1}H-NMR

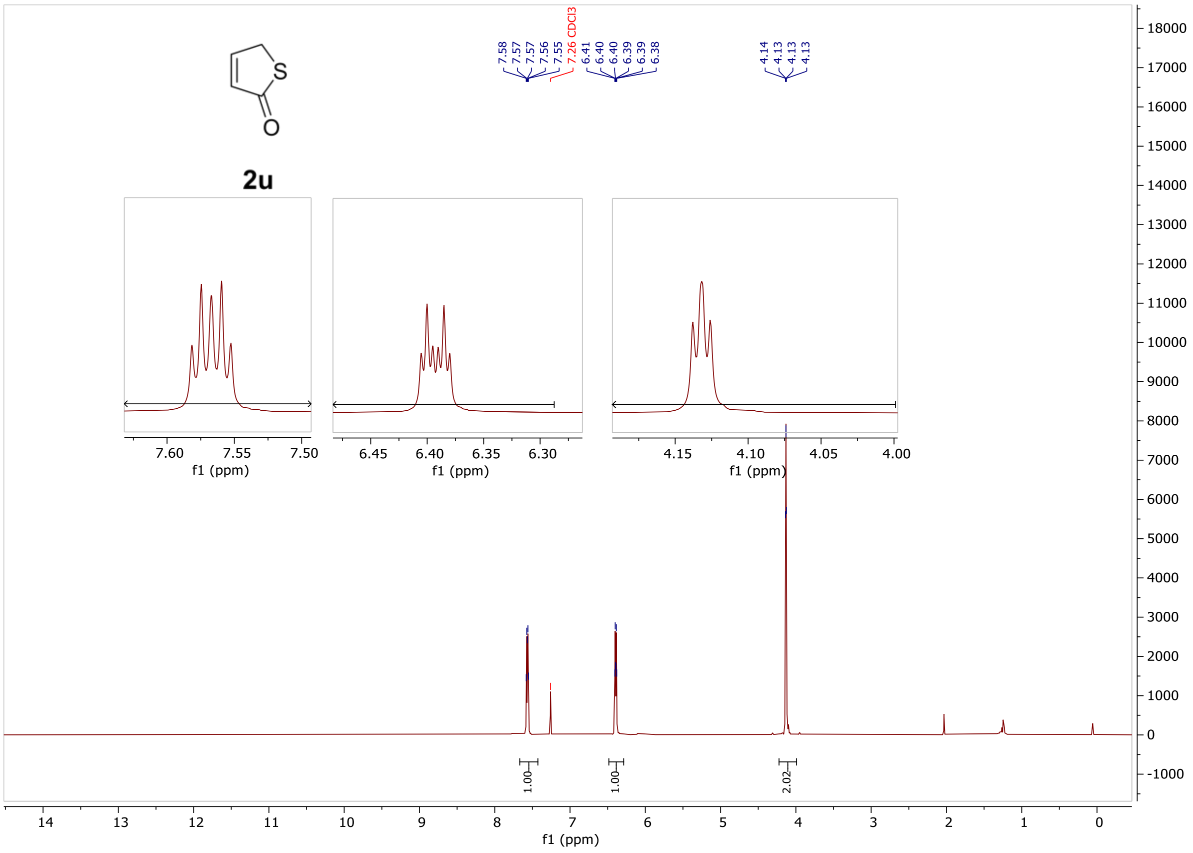

^{13}C-NMR

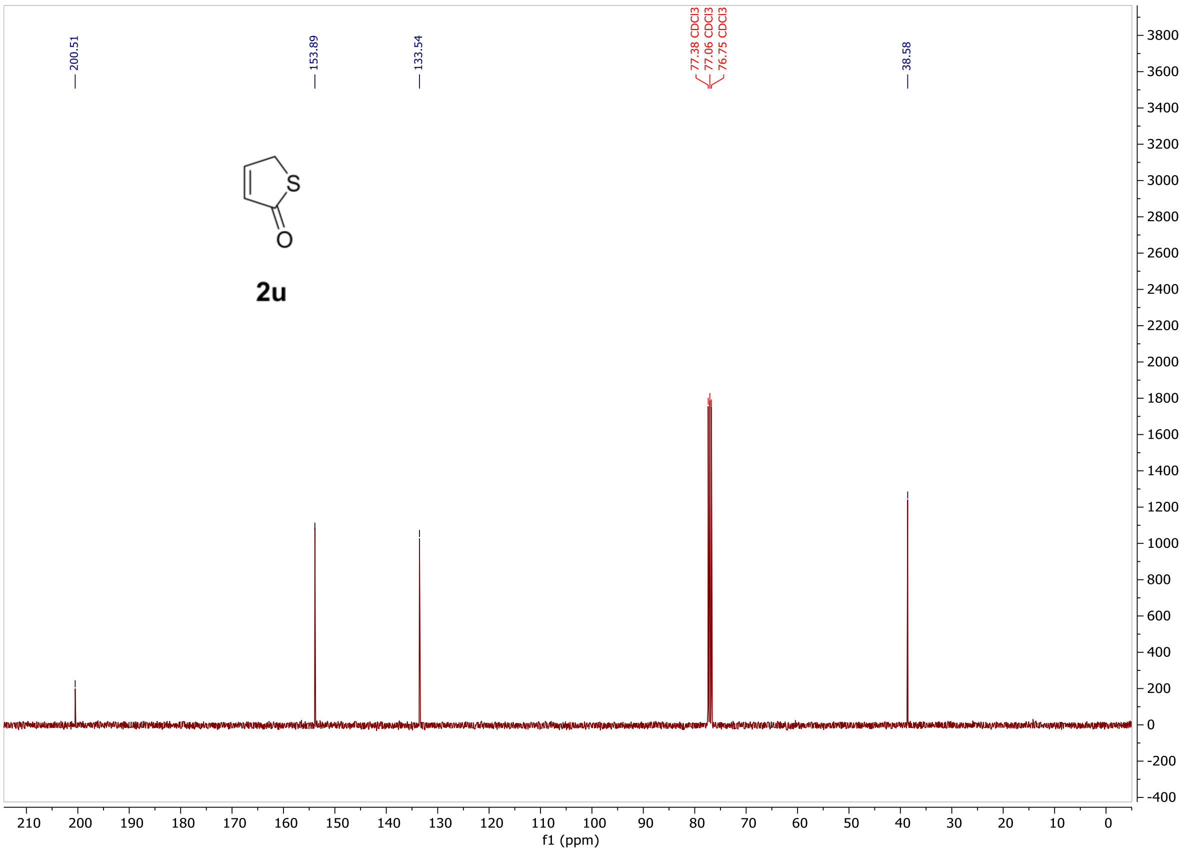

^{1}H-NMR

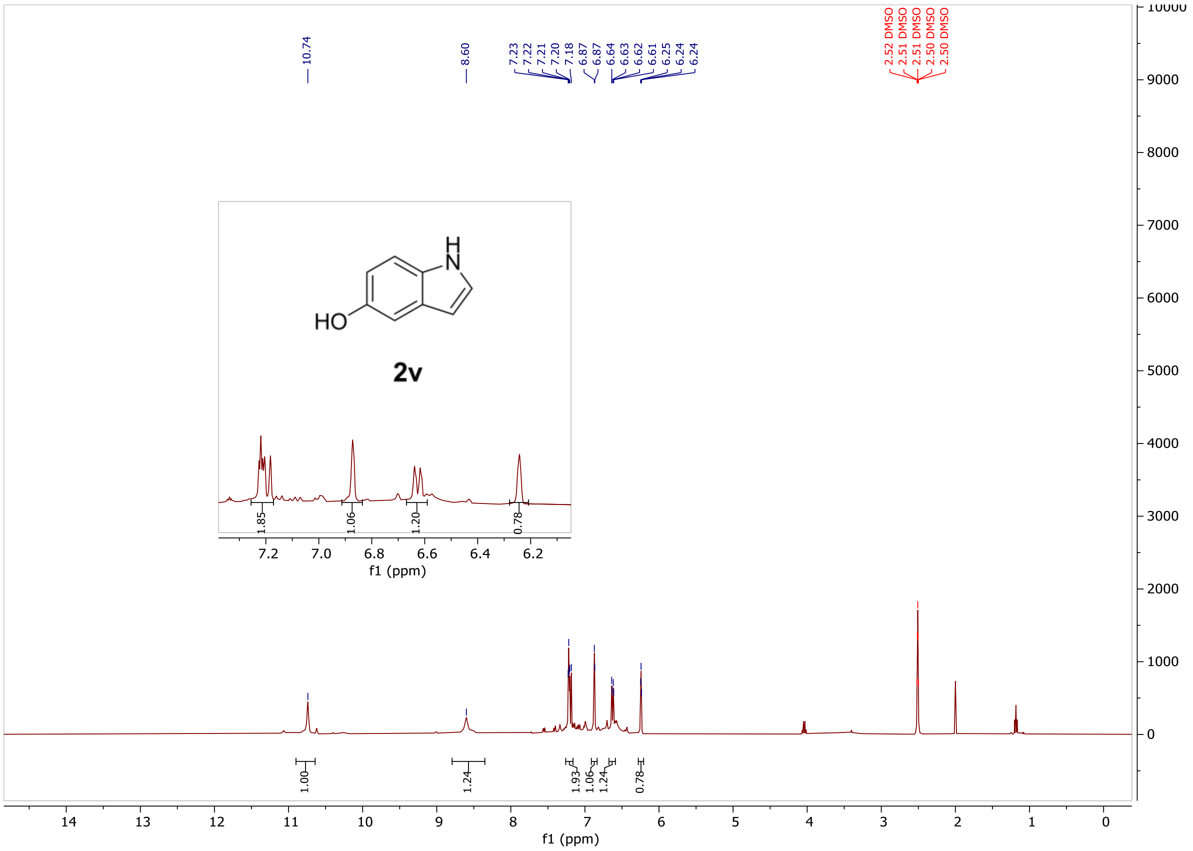

^{13}C-NMR

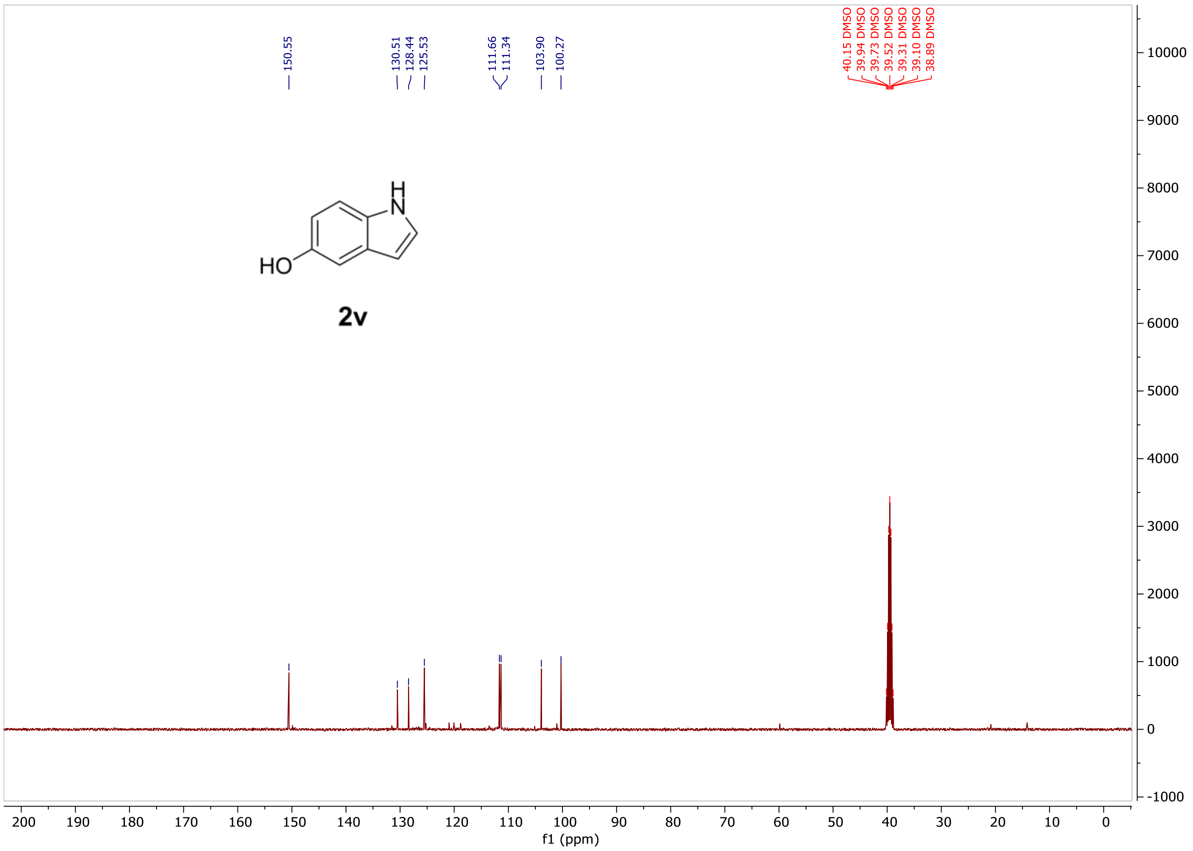

^{1}H-NMR

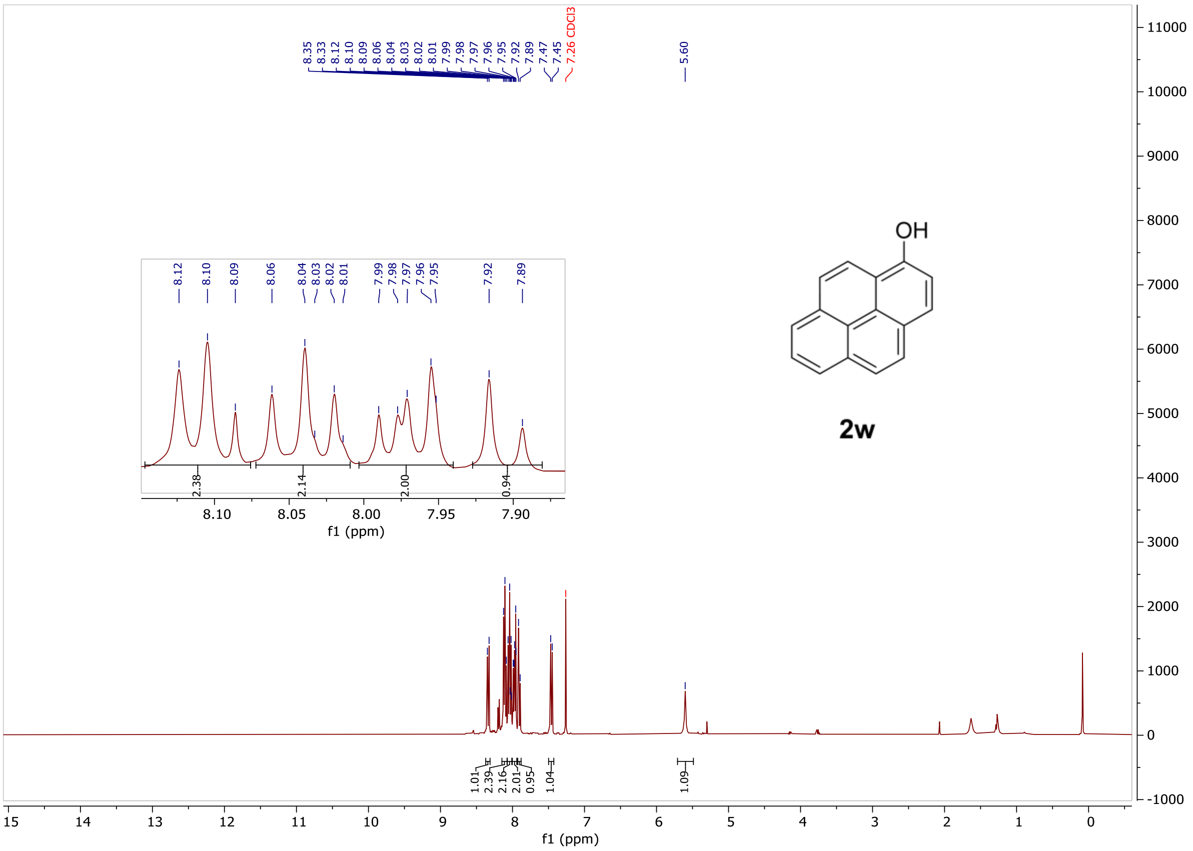

^{13}C-NMR

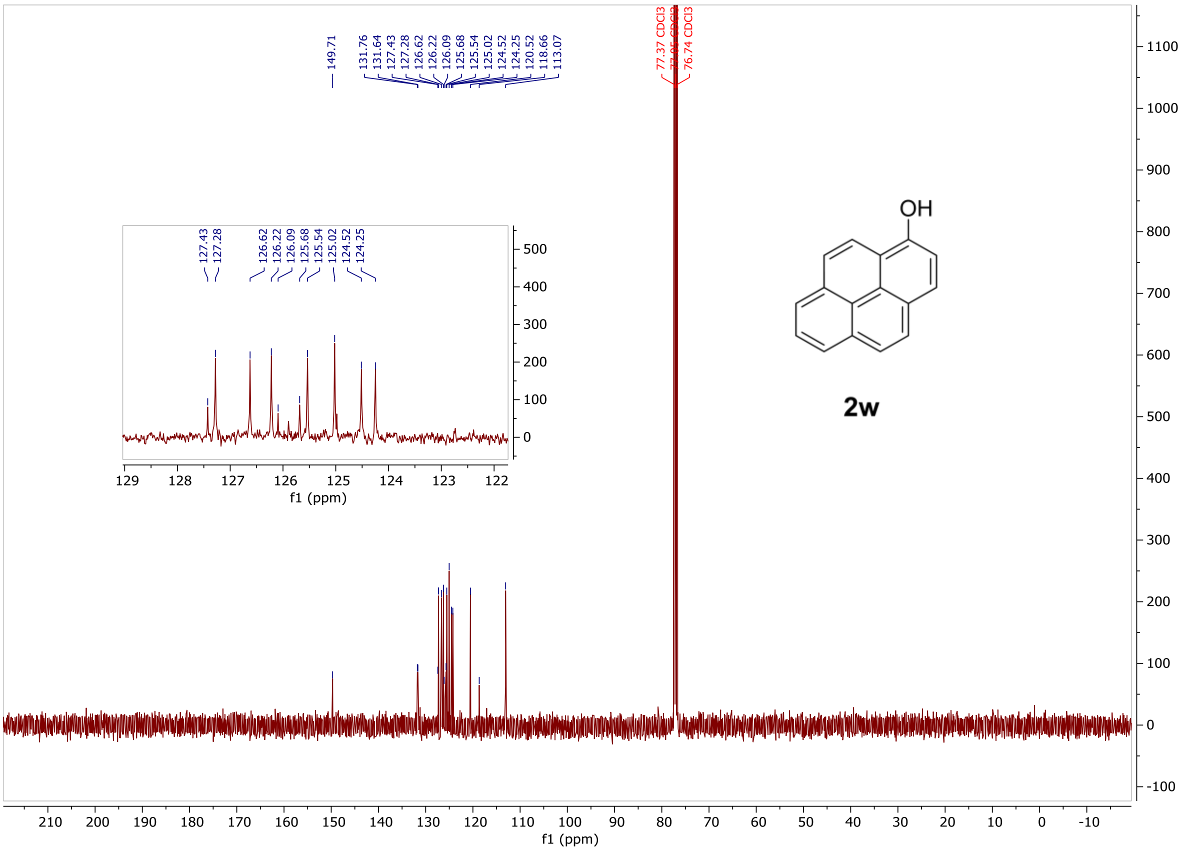

^{1}H-NMR

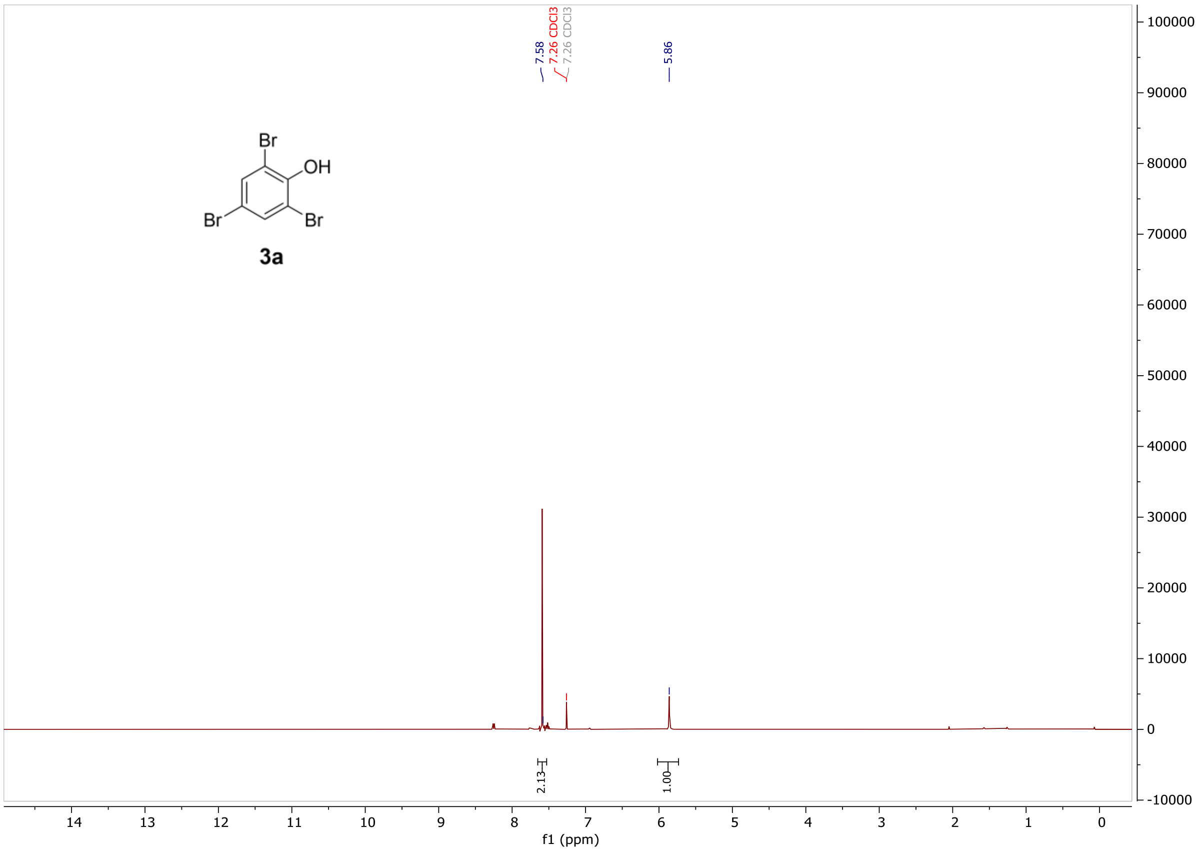

^{13}C-NMR

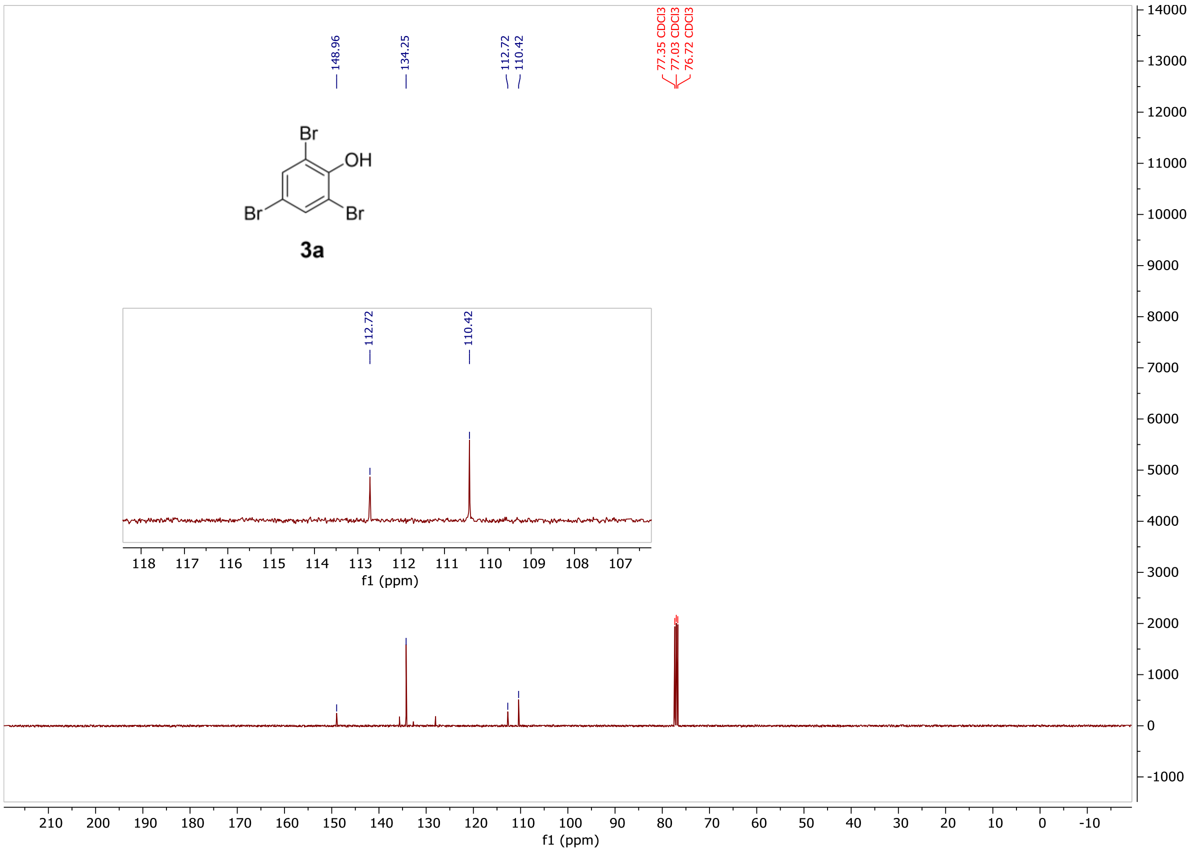

^{1}H-NMR

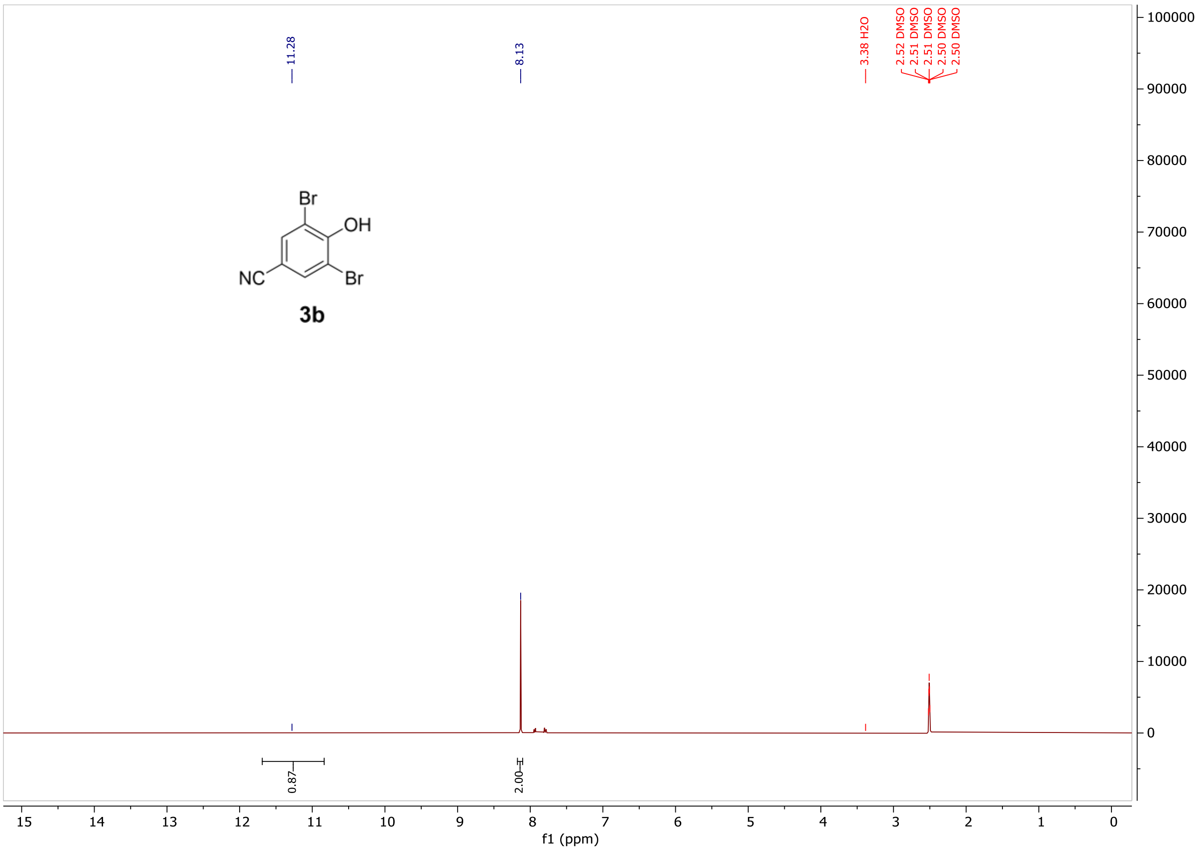

^{13}C-NMR

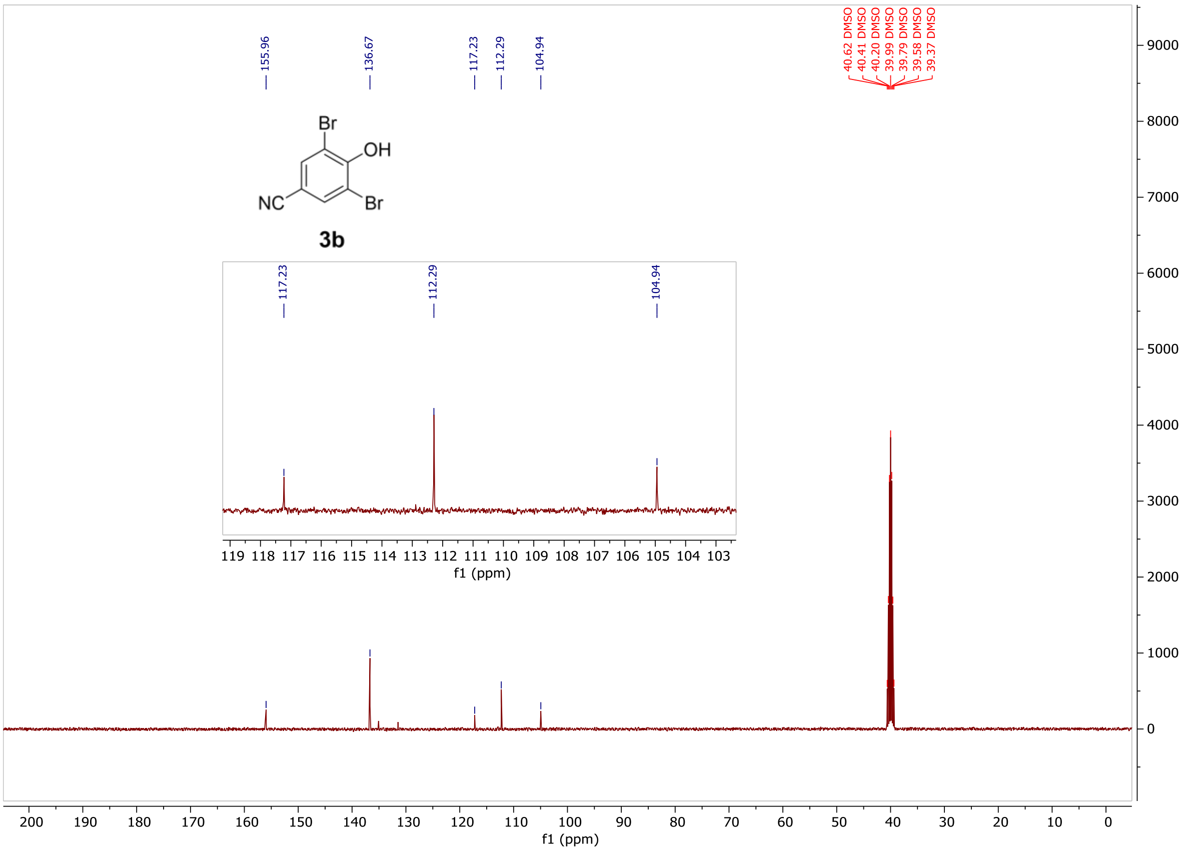

^{1}H-NMR

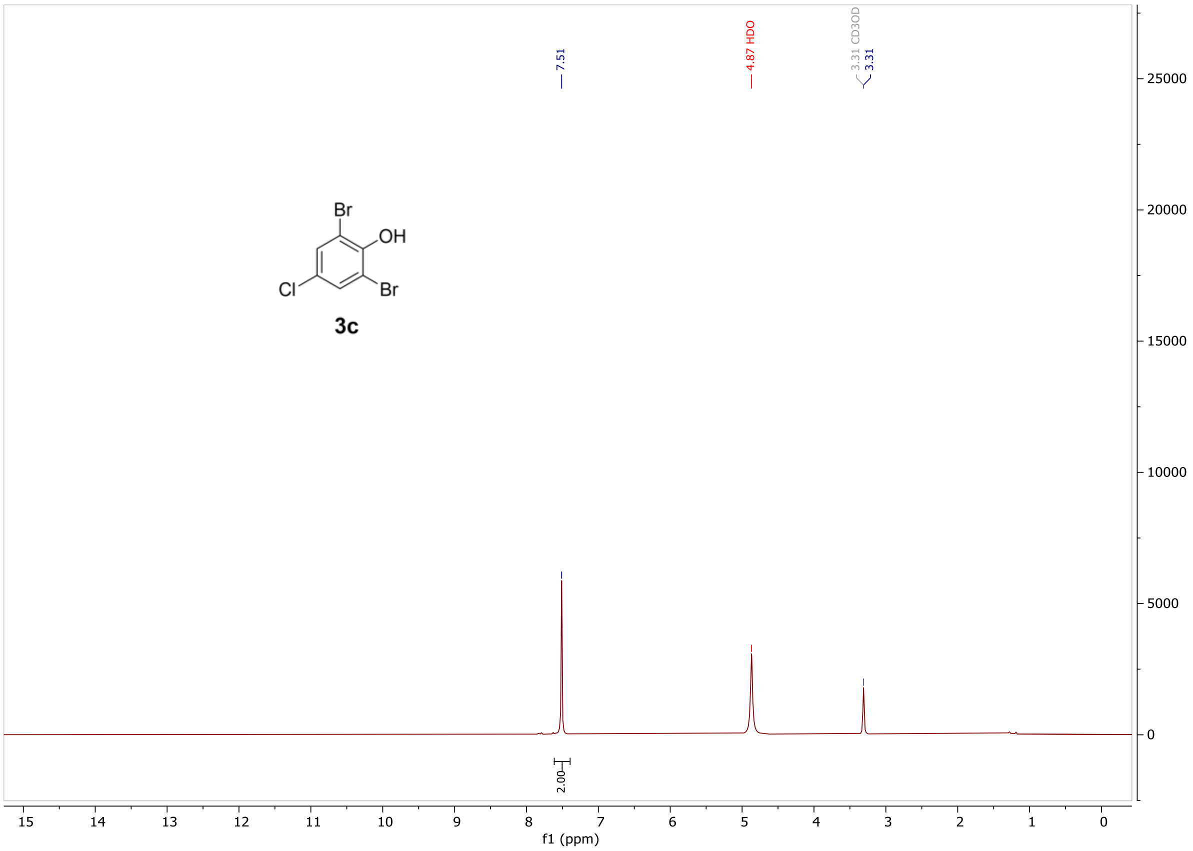

^{13}C-NMR

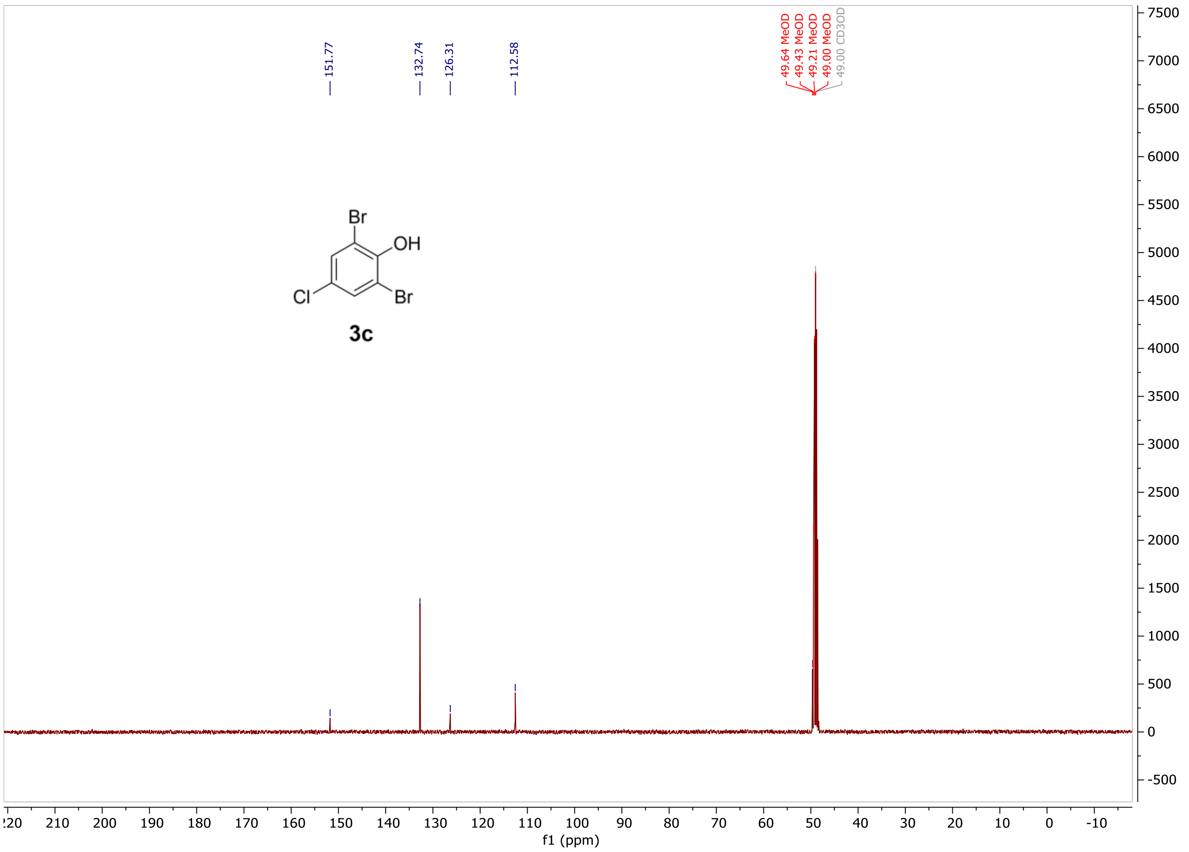

^{1}H-NMR

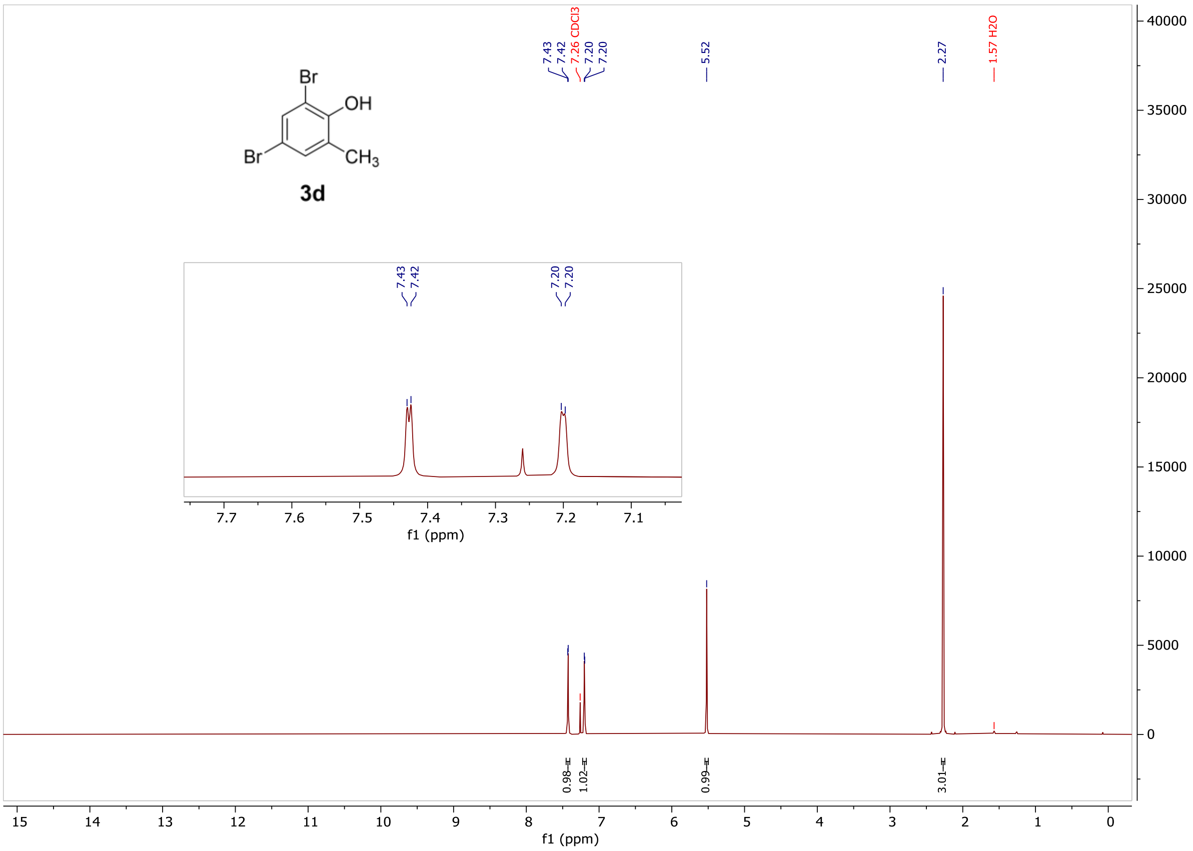

^{13}C-NMR

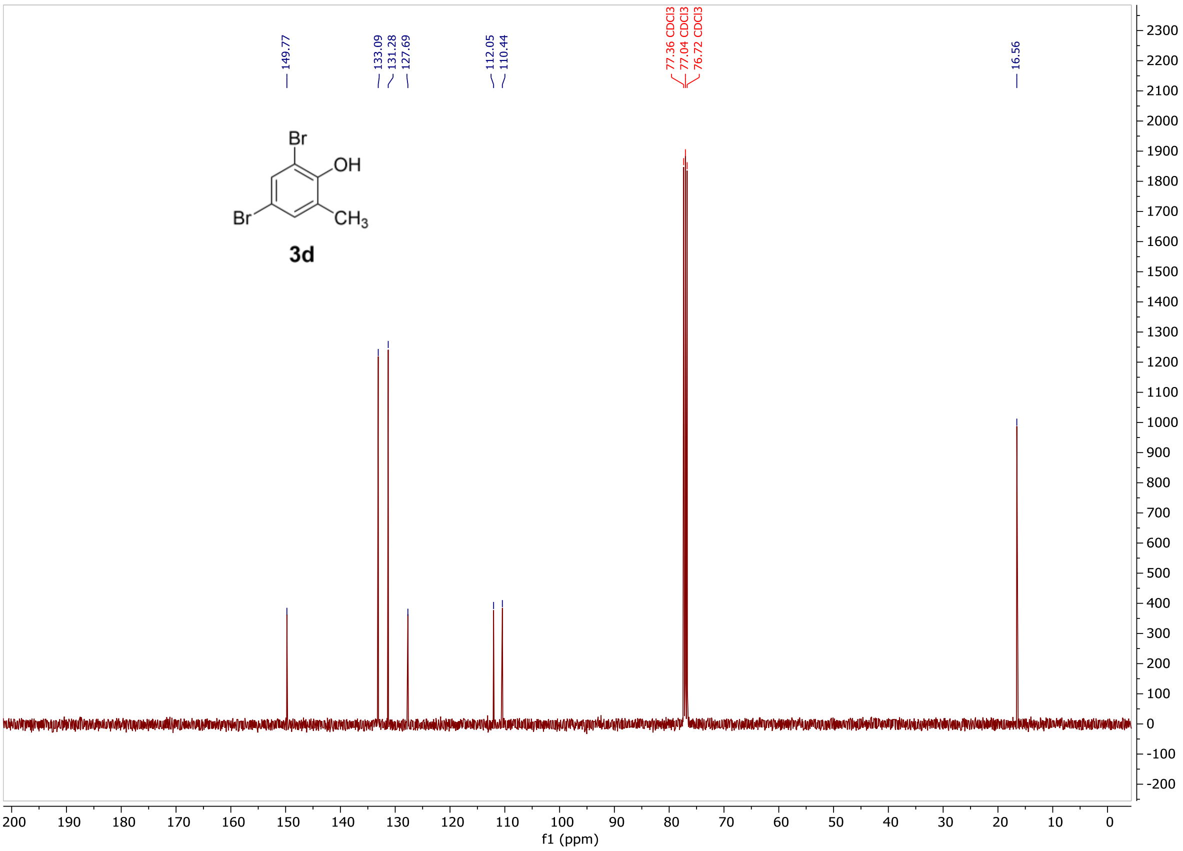

^{1}H-NMR

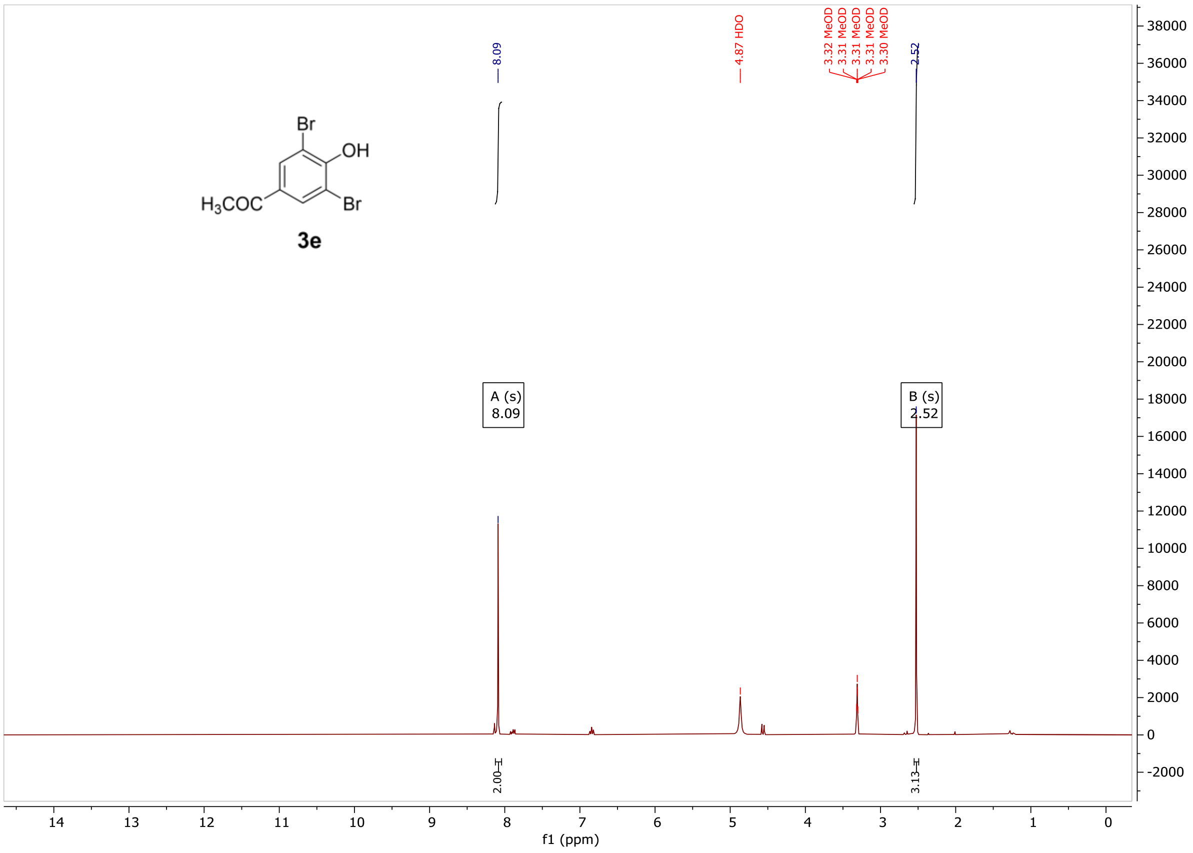

^{13}C-NMR

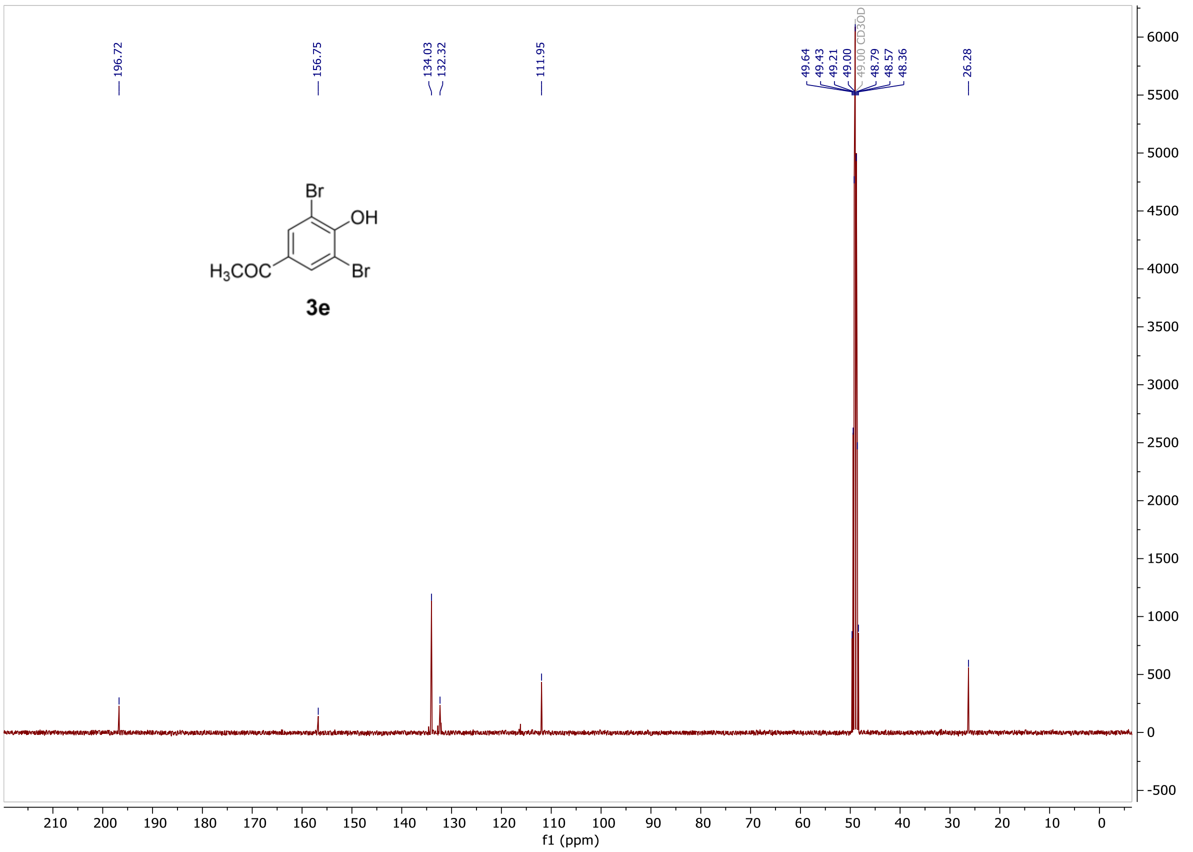

^{1}H-NMR

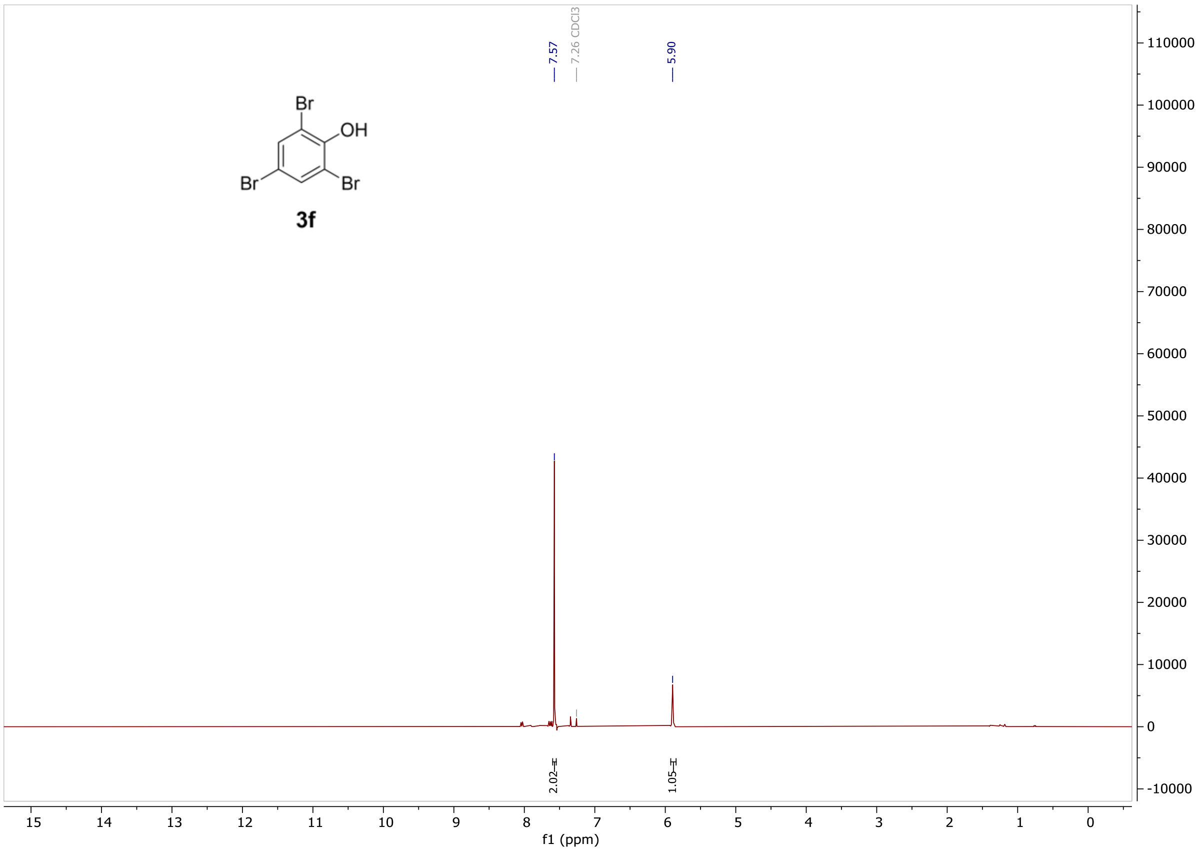

^{13}C-NMR

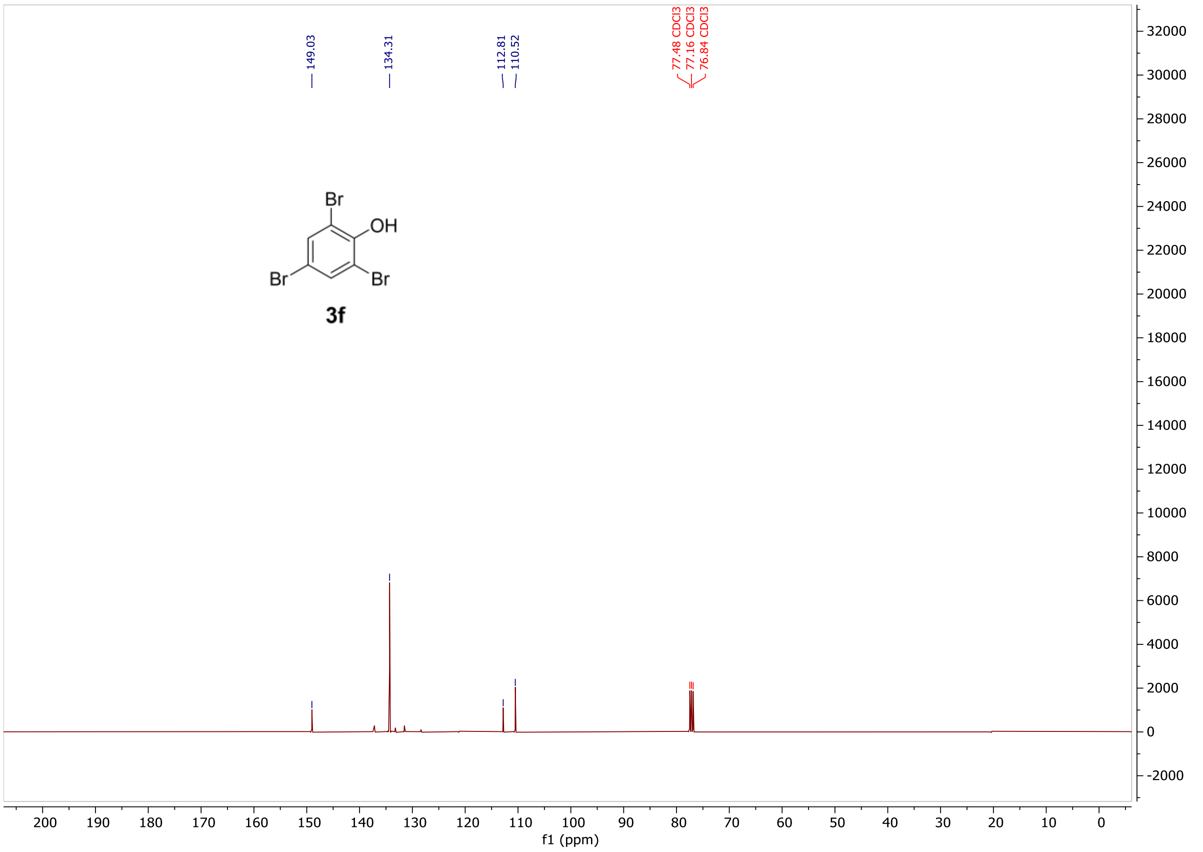

^{1}H-NMR

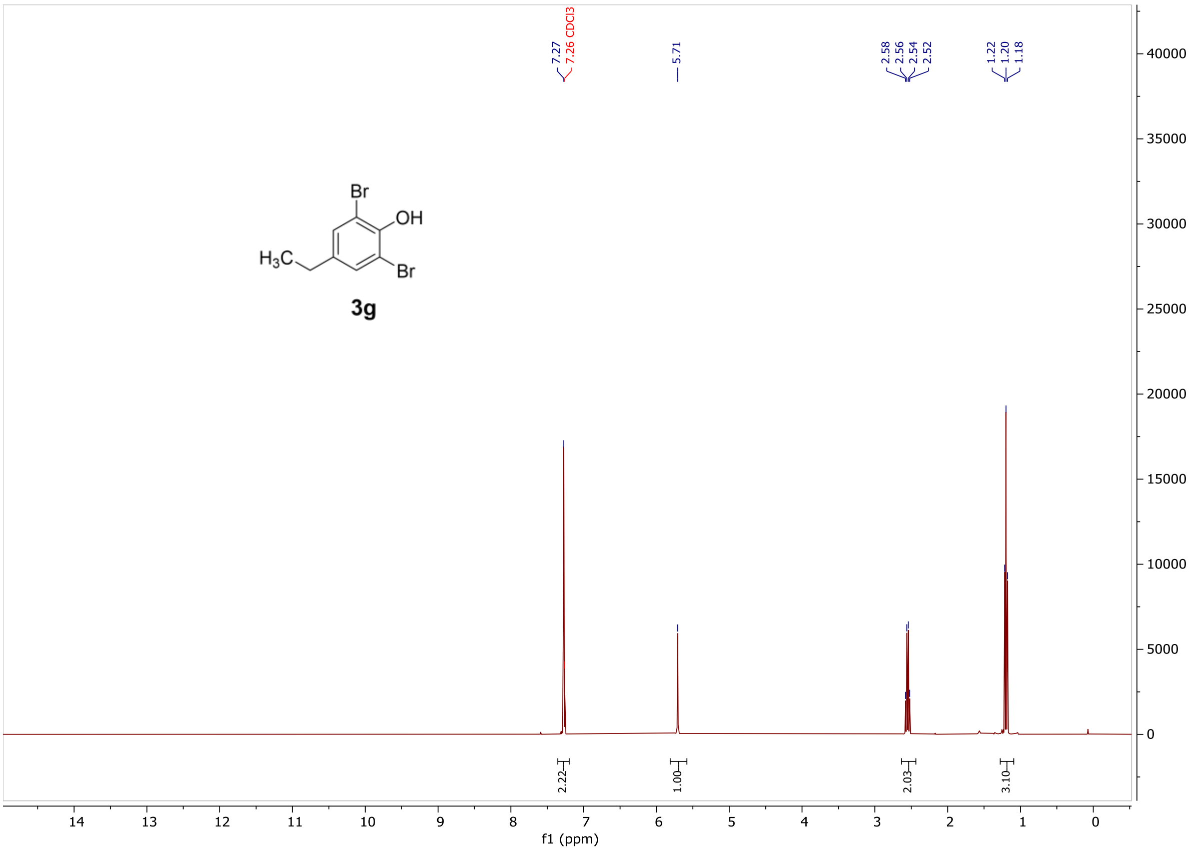

^{13}C-NMR

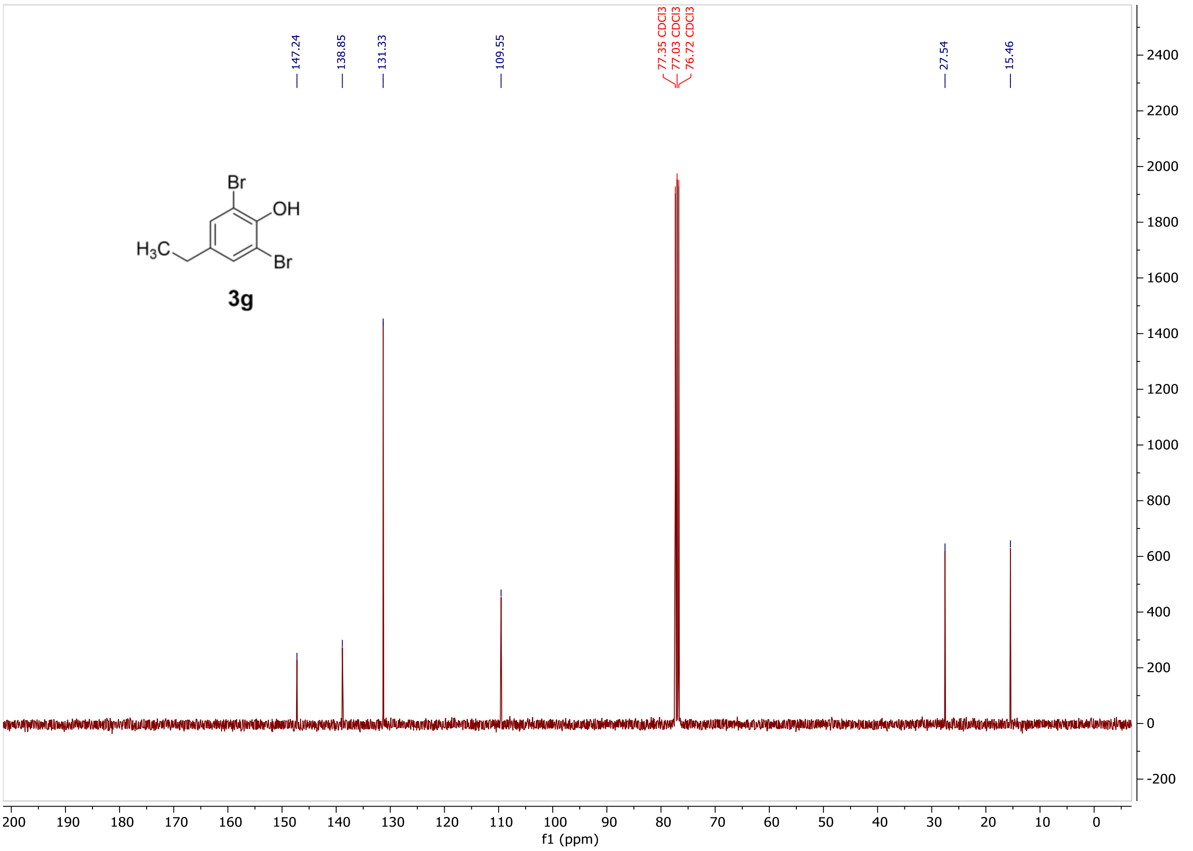

^{1}H-NMR

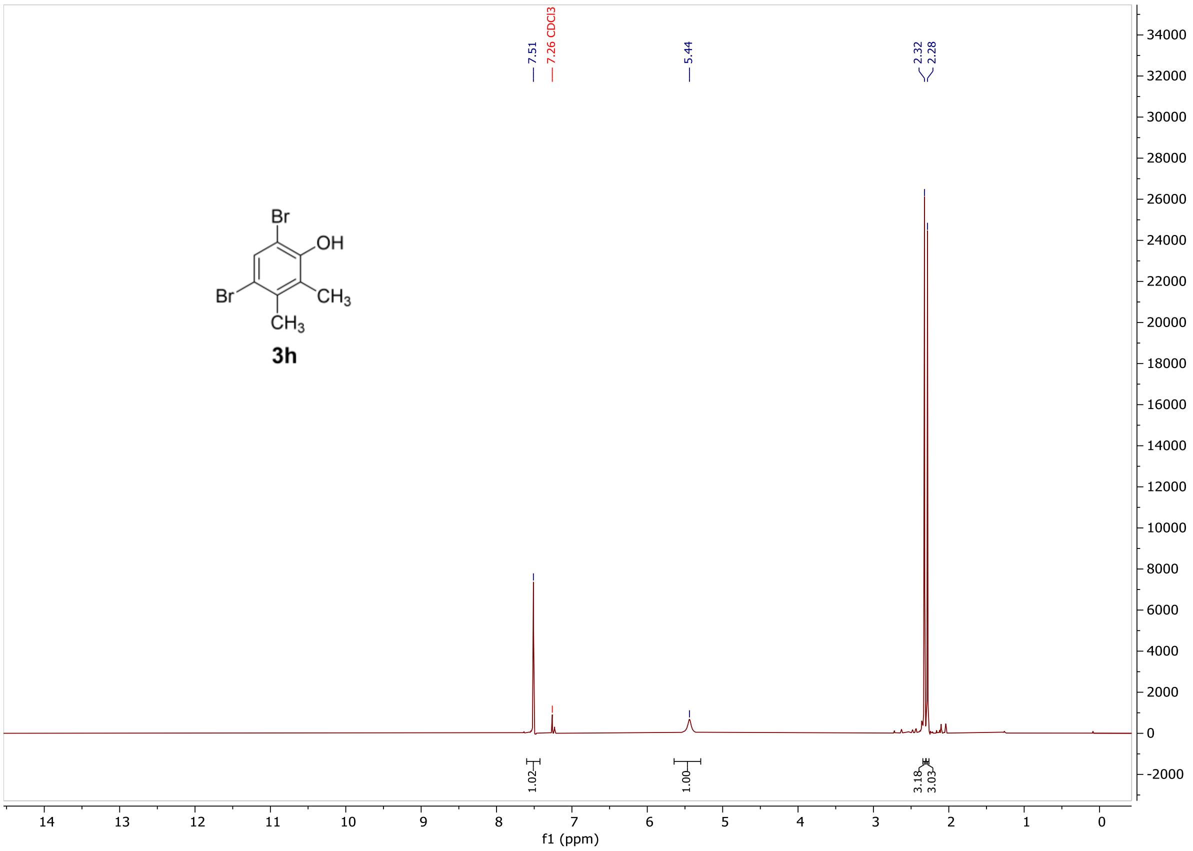

^{13}C-NMR

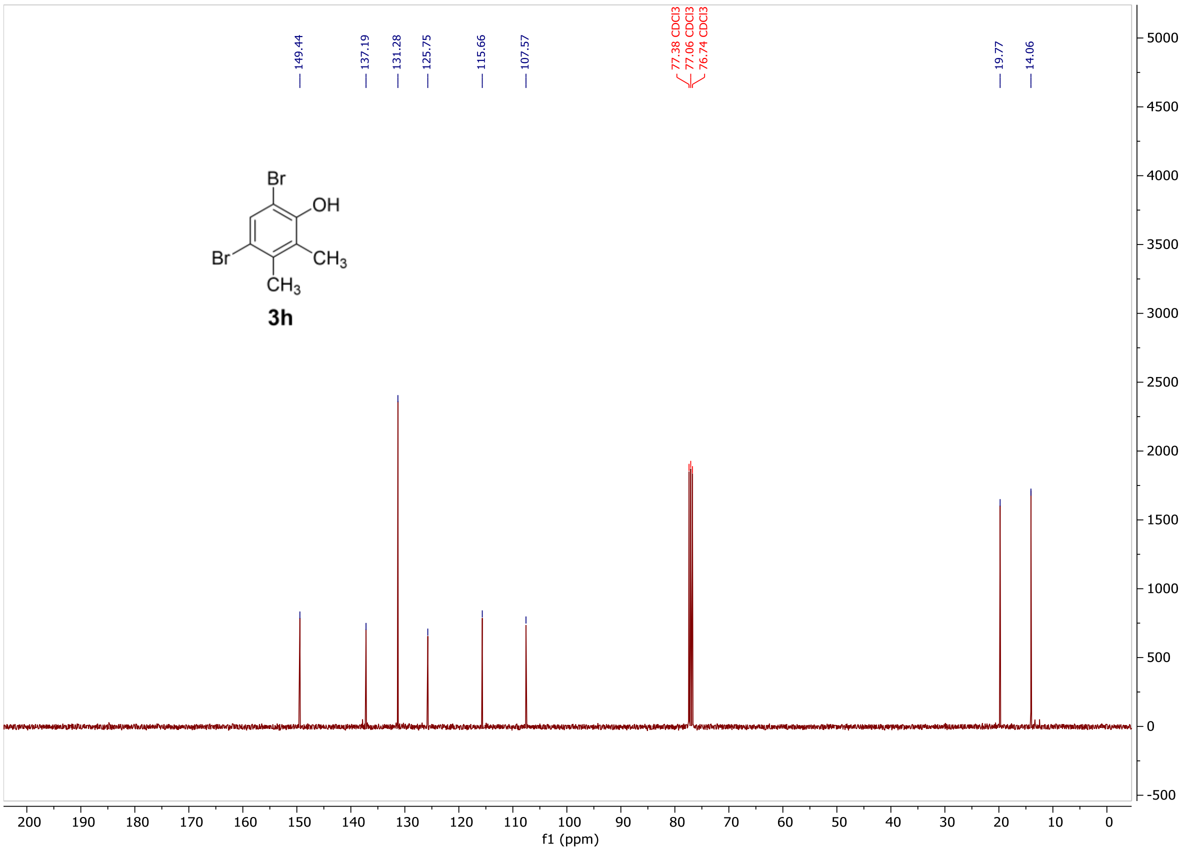

^{1}H-NMR

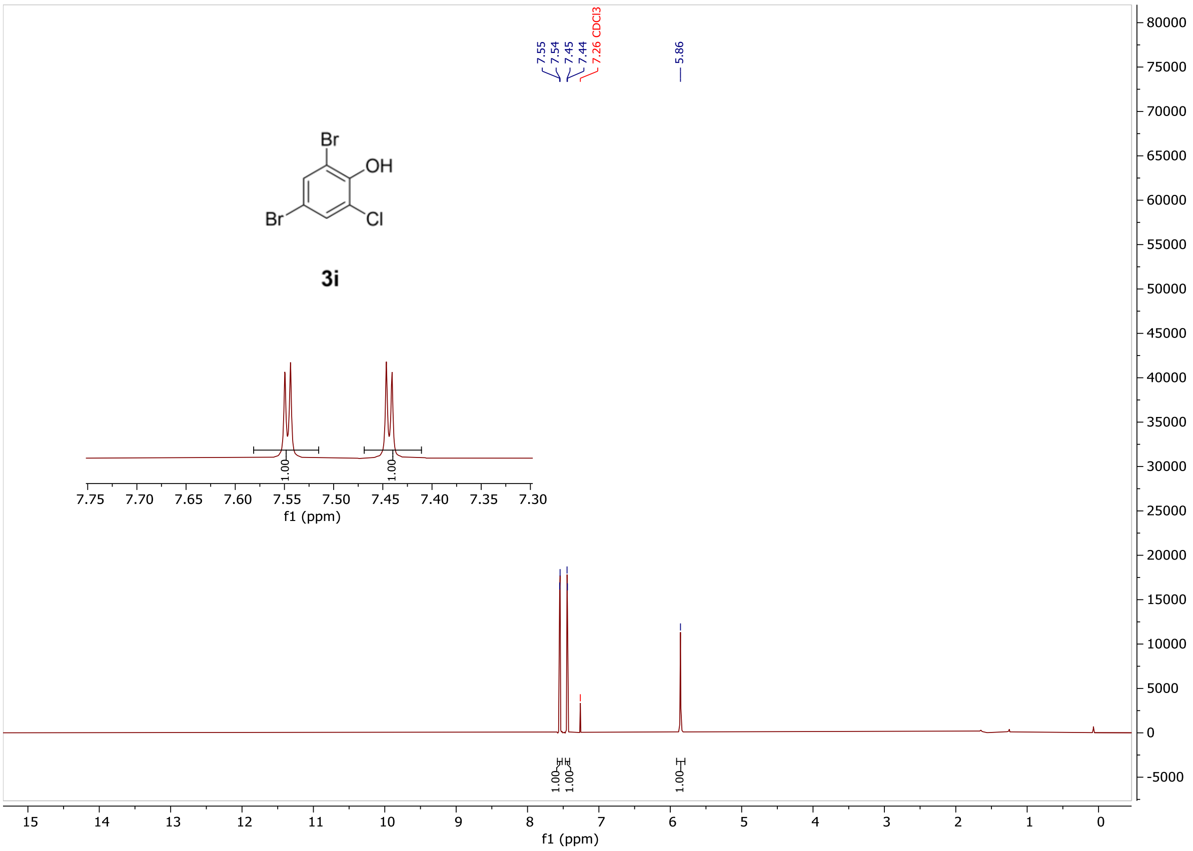

^{13}C-NMR

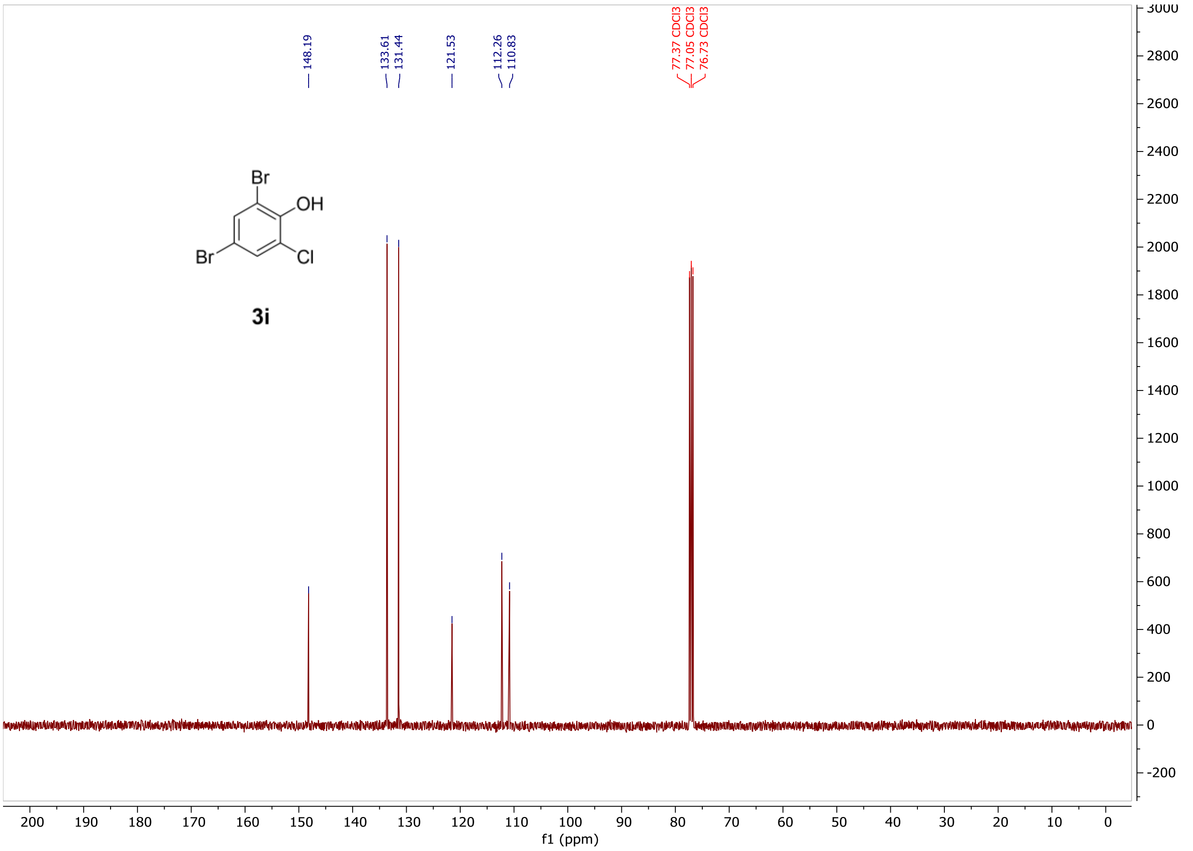

^{1}H-NMR

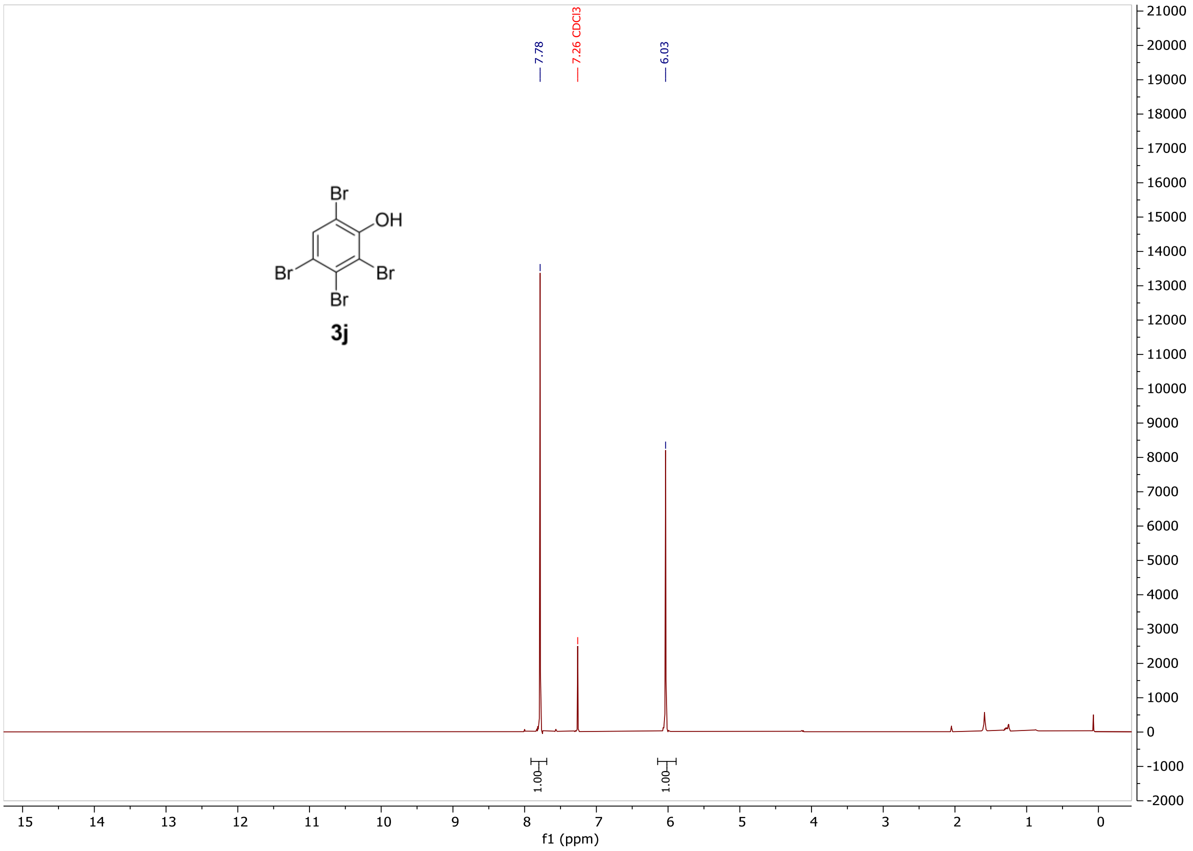

^{13}C-NMR

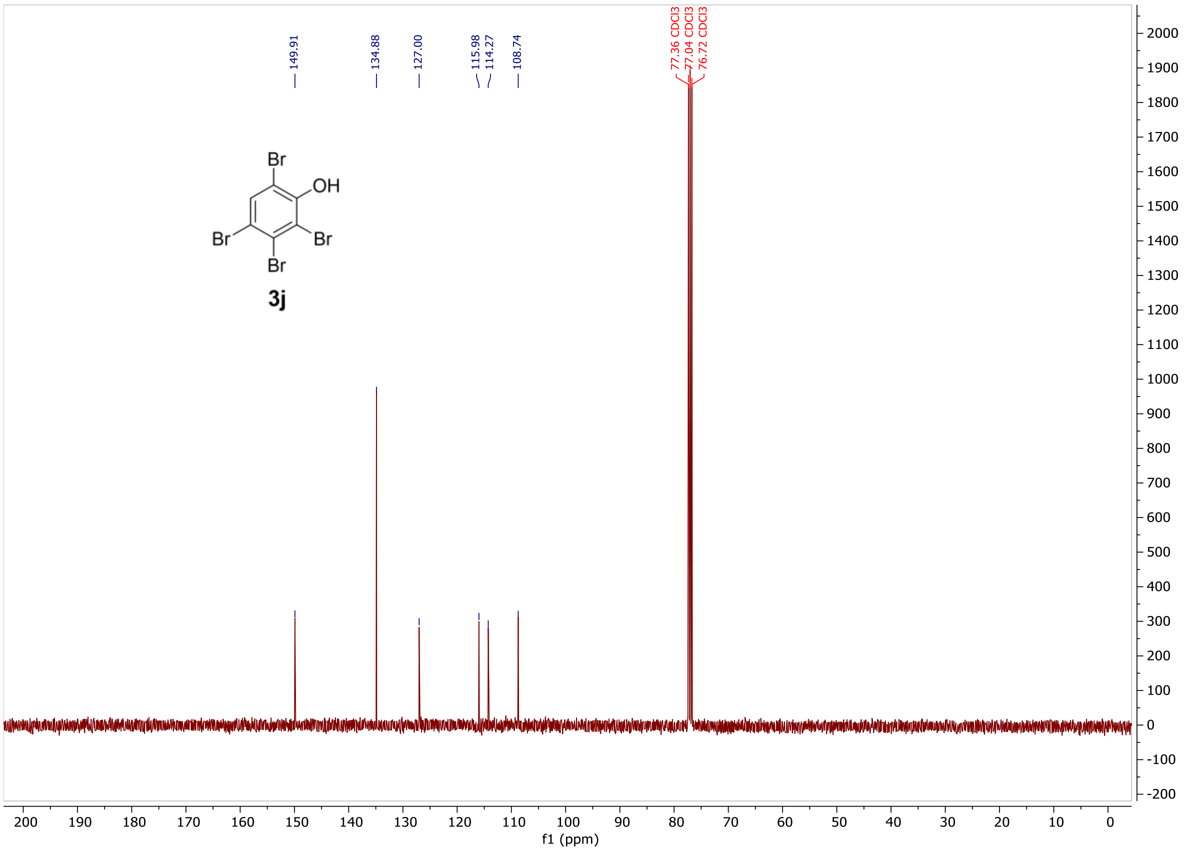

^{1}H-NMR

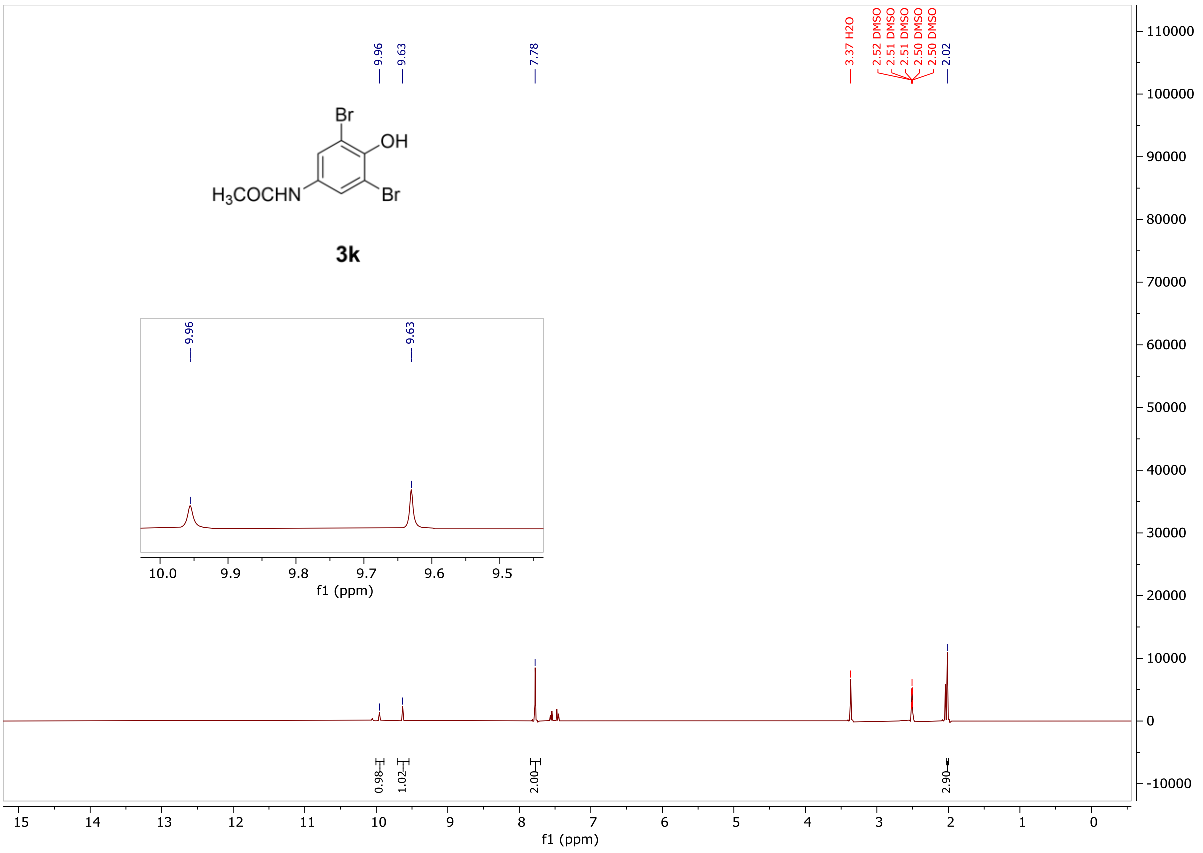

^{13}C-NMR

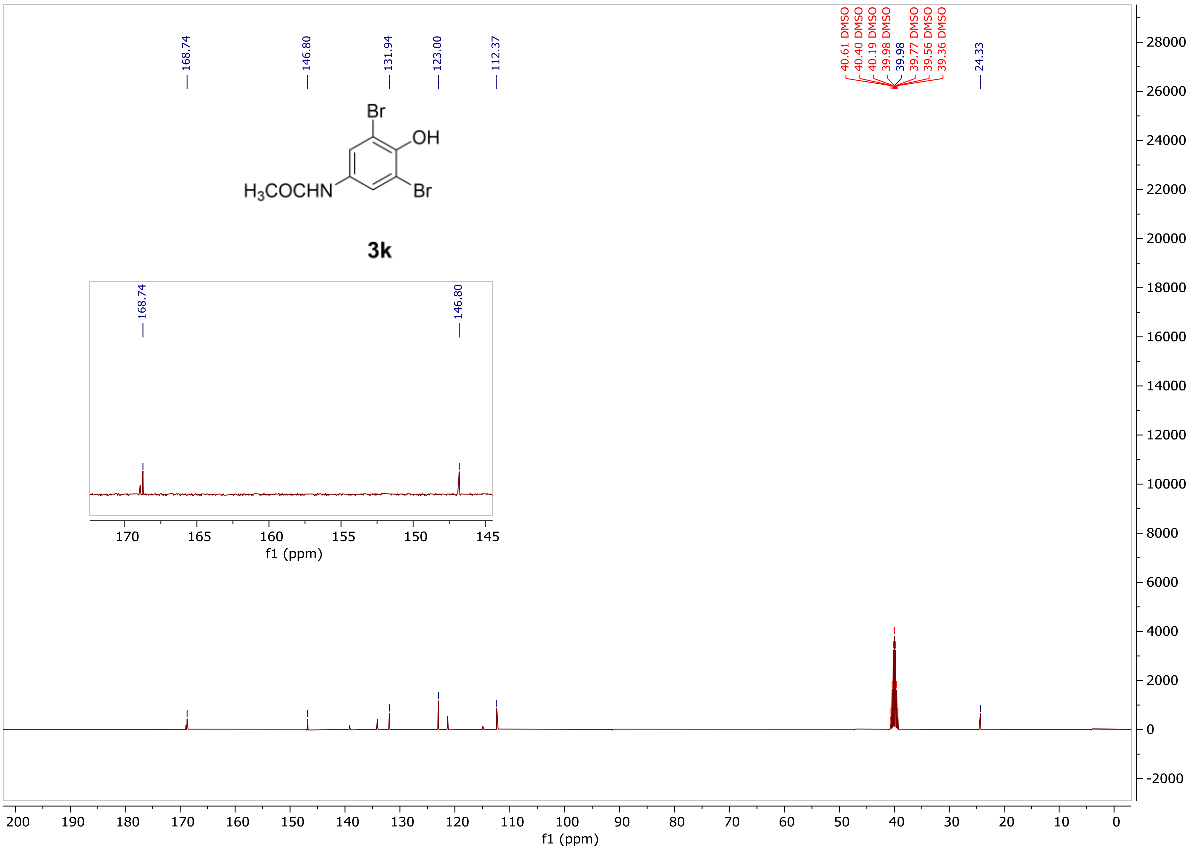

^{1}H-NMR

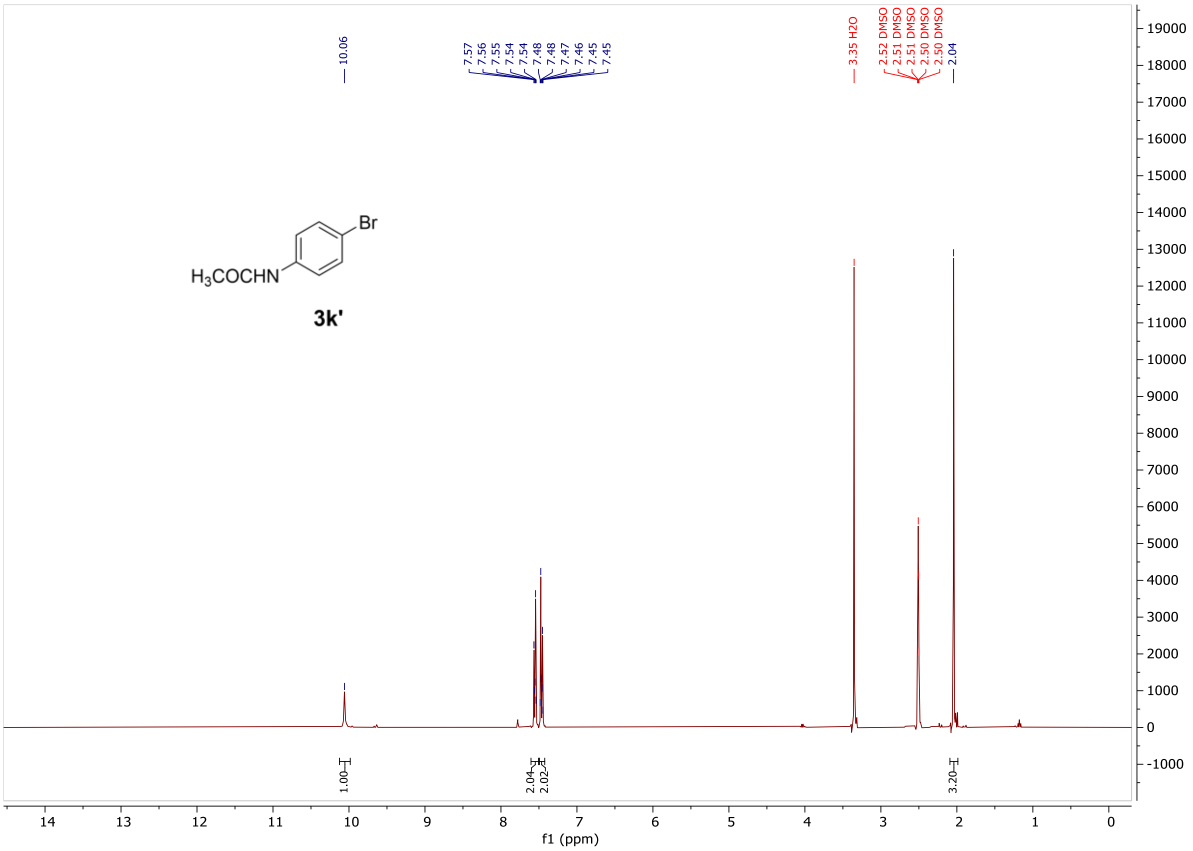

^{13}C-NMR

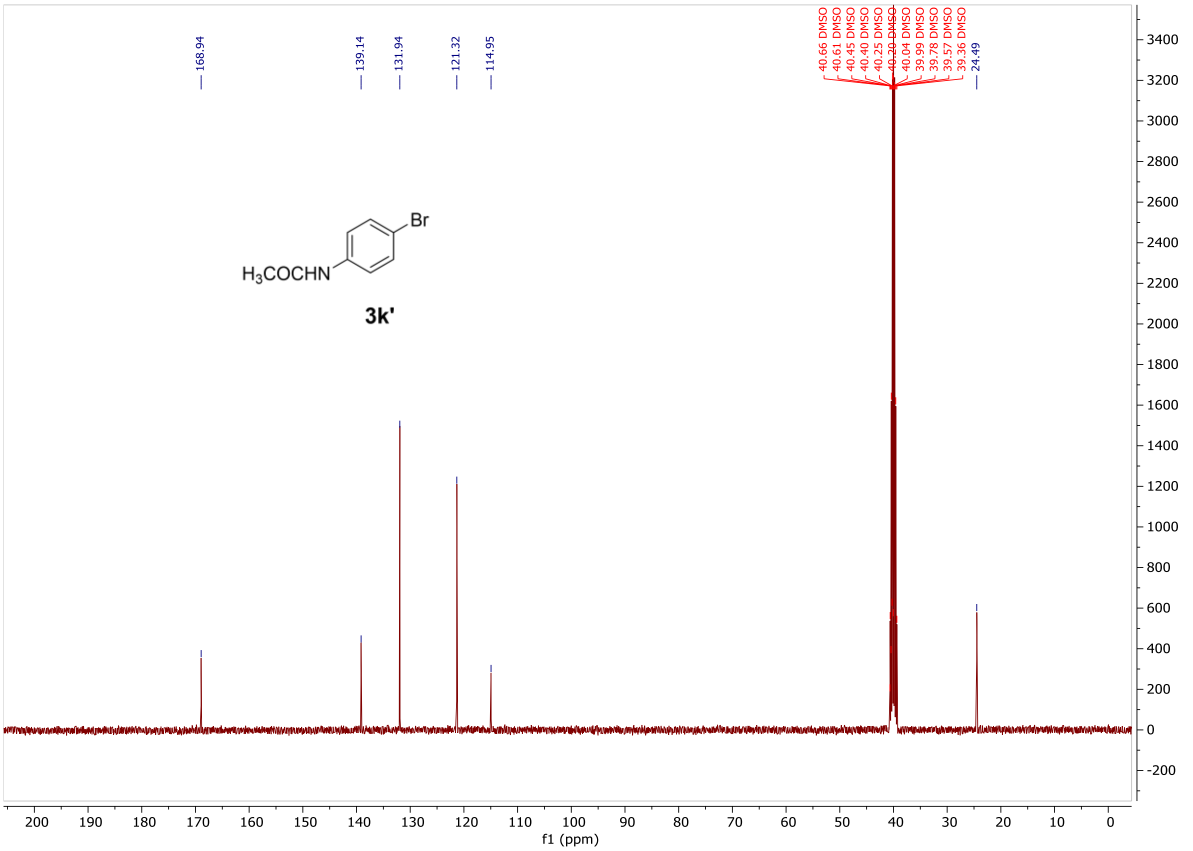

^{1}H-NMR

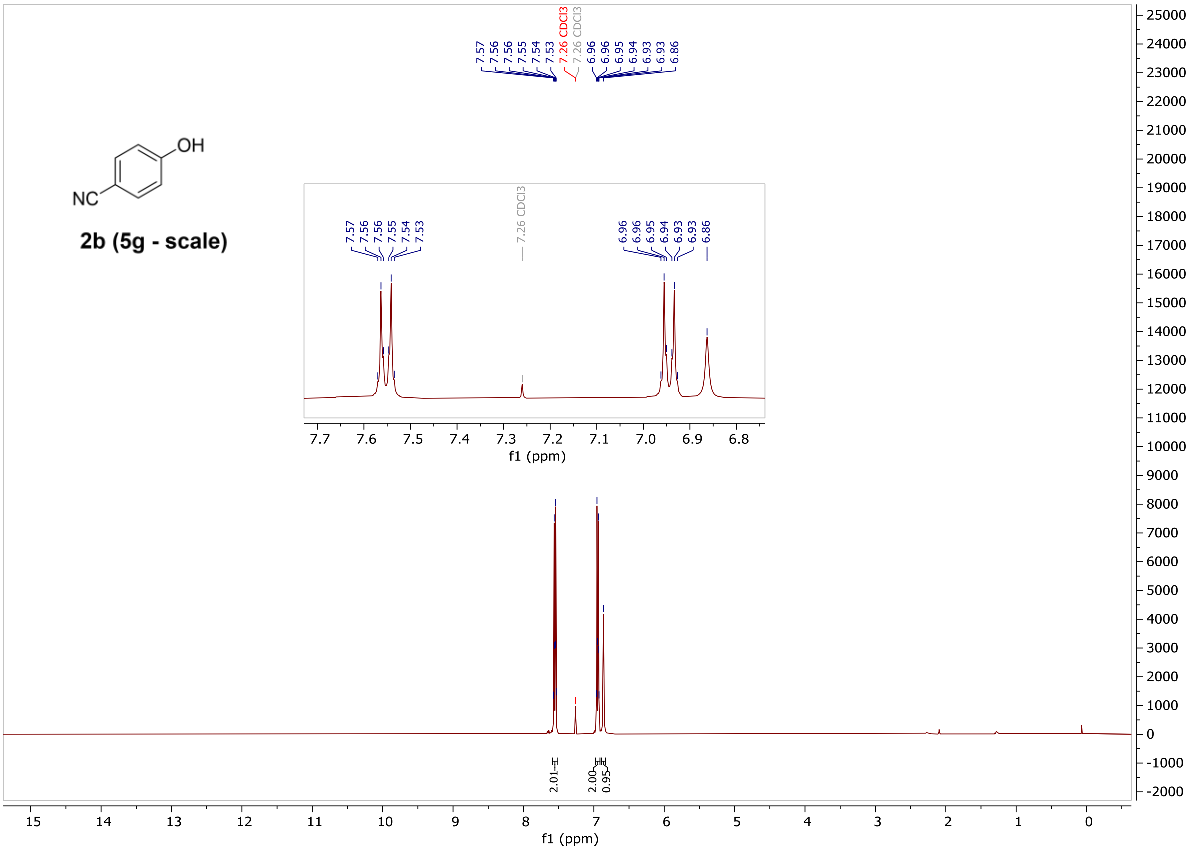

^{13}C-NMR

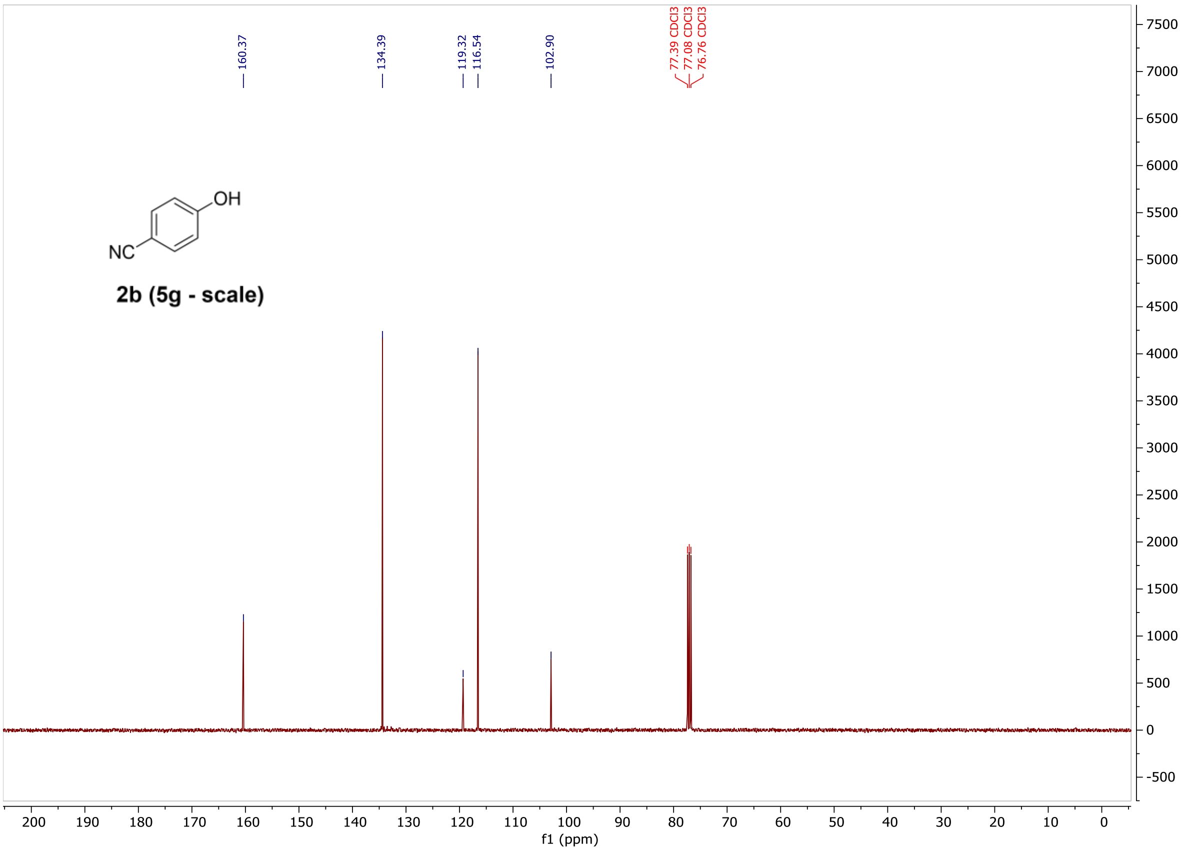

^{1}H-NMR

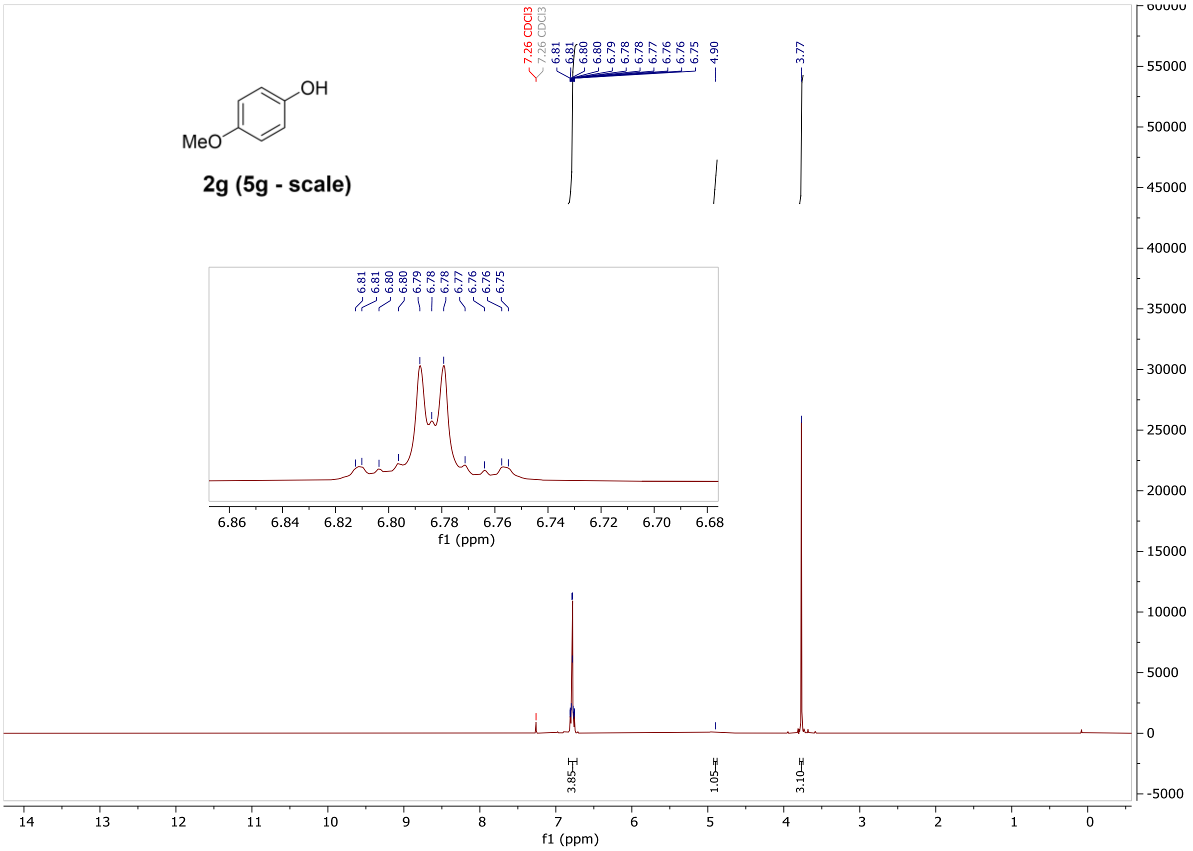

^{13}C-NMR

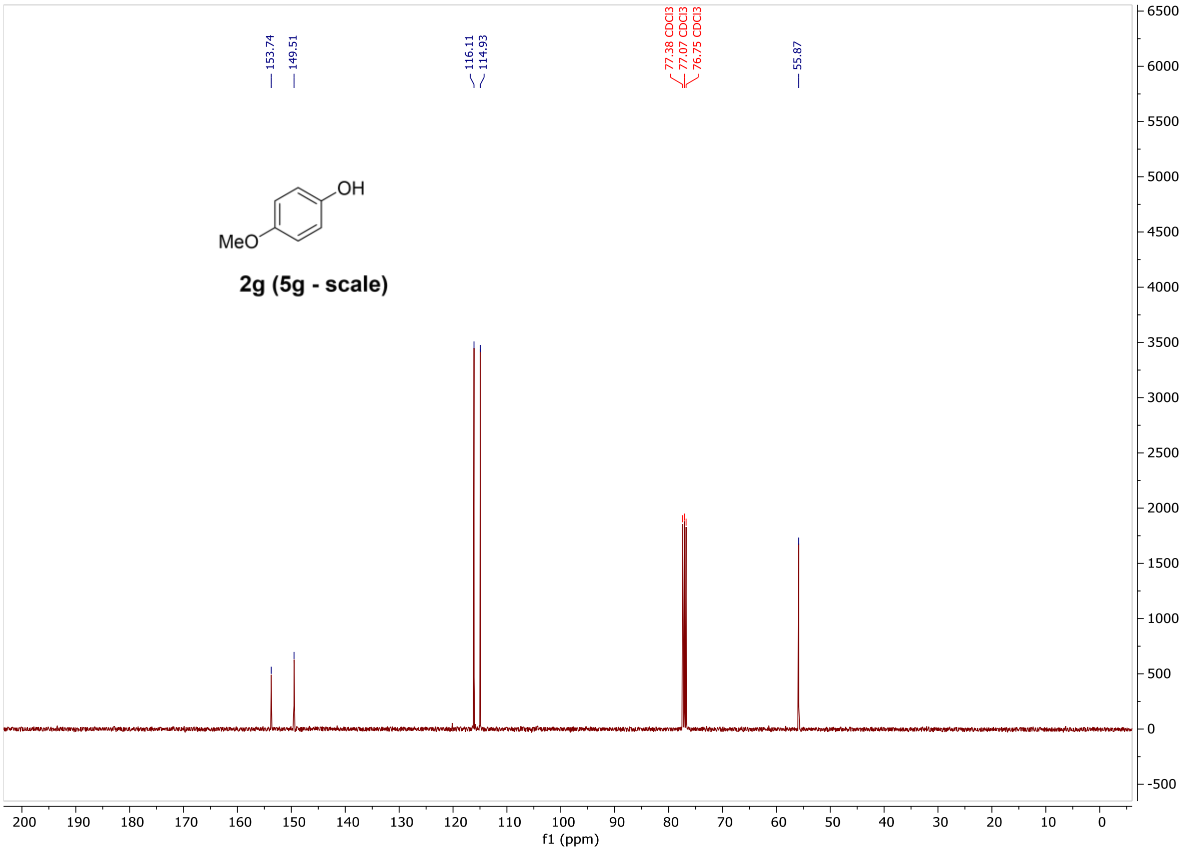

^{1}H-NMR

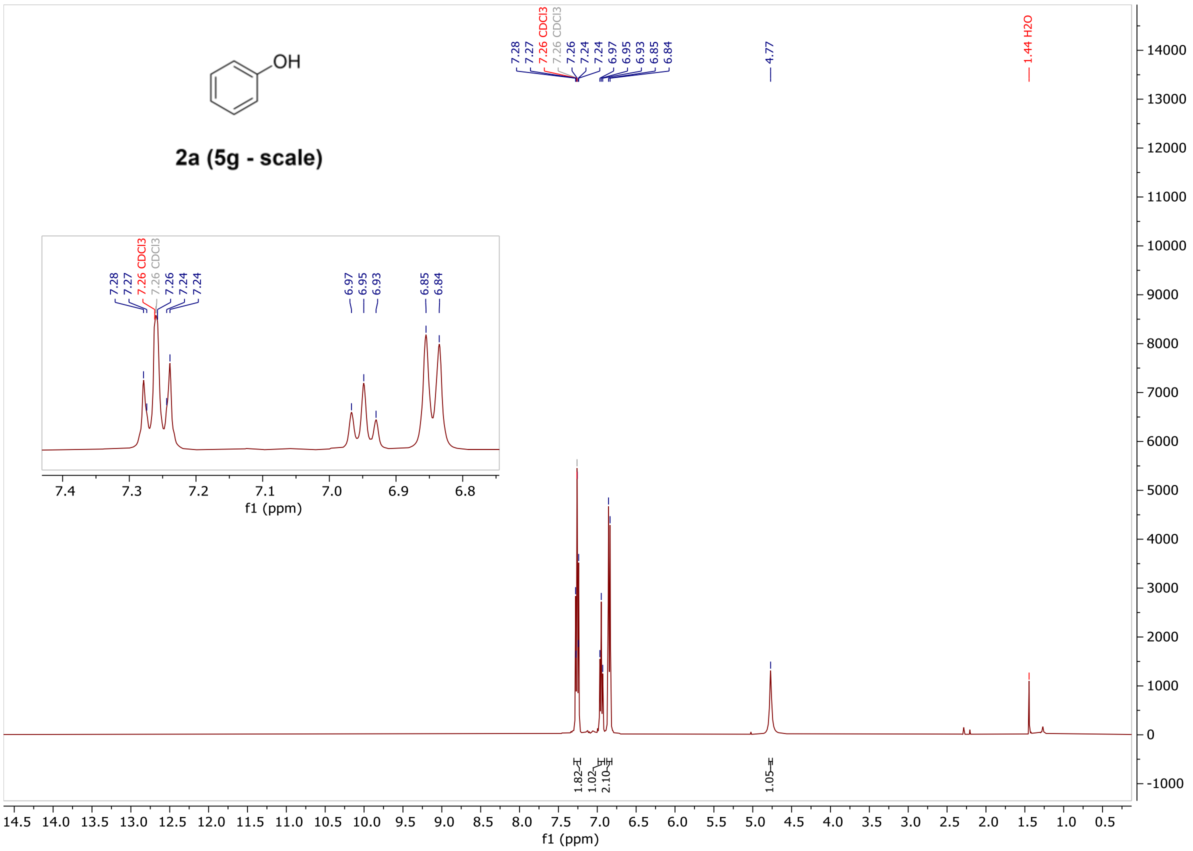

^{13}C-NMR

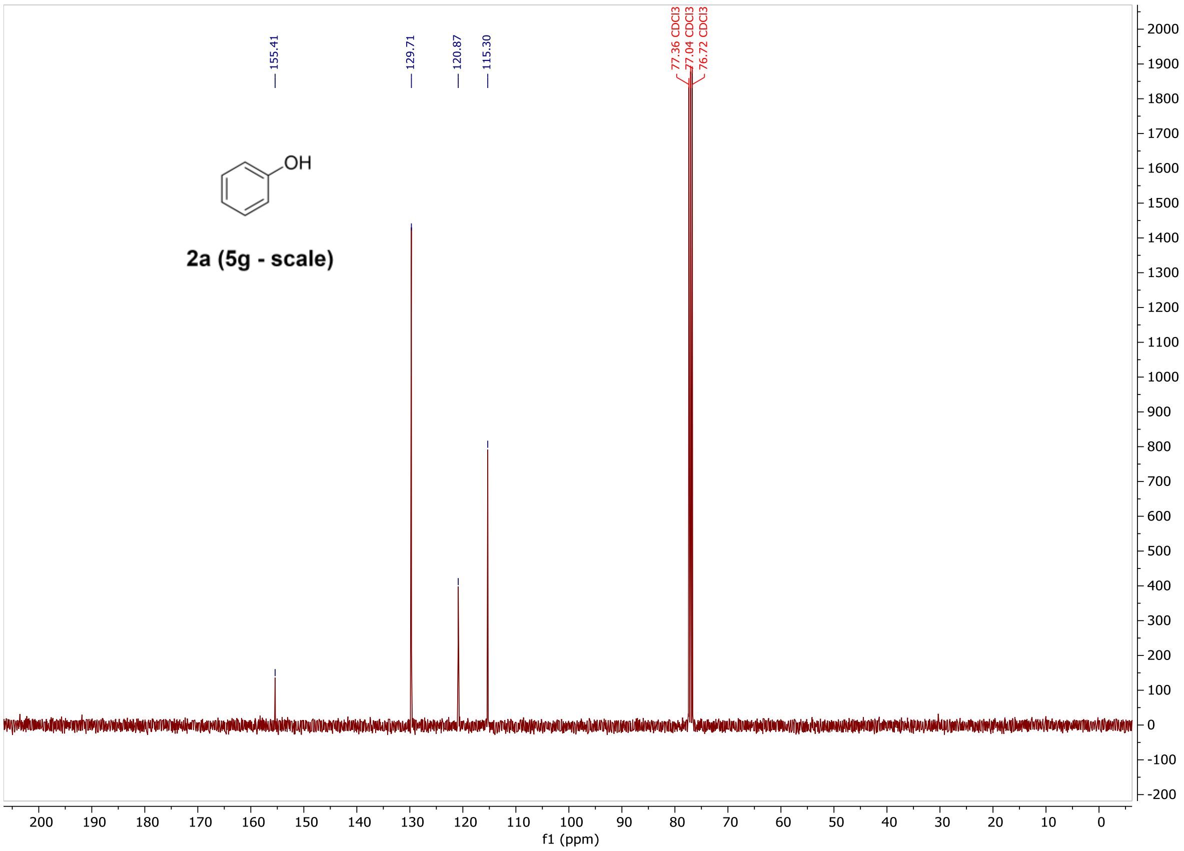

^{1}H-NMR

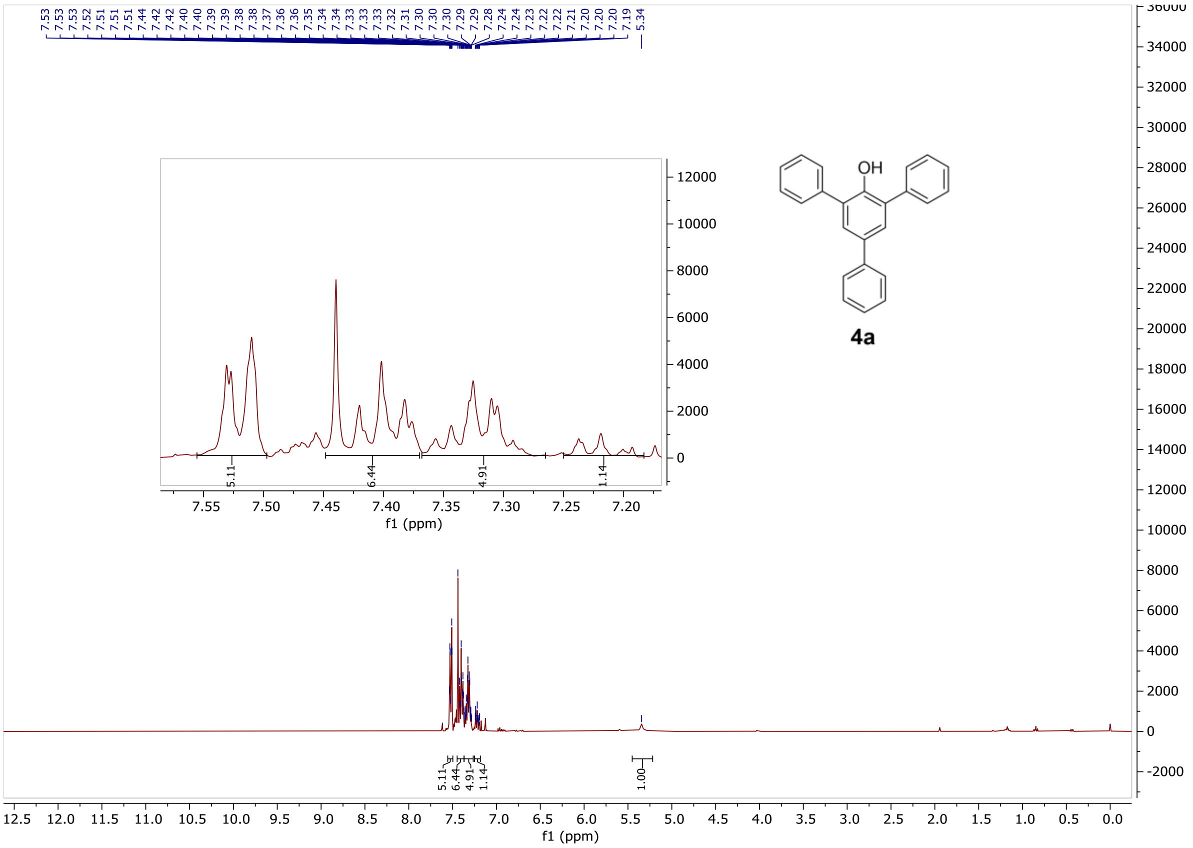

^{13}C-NMR

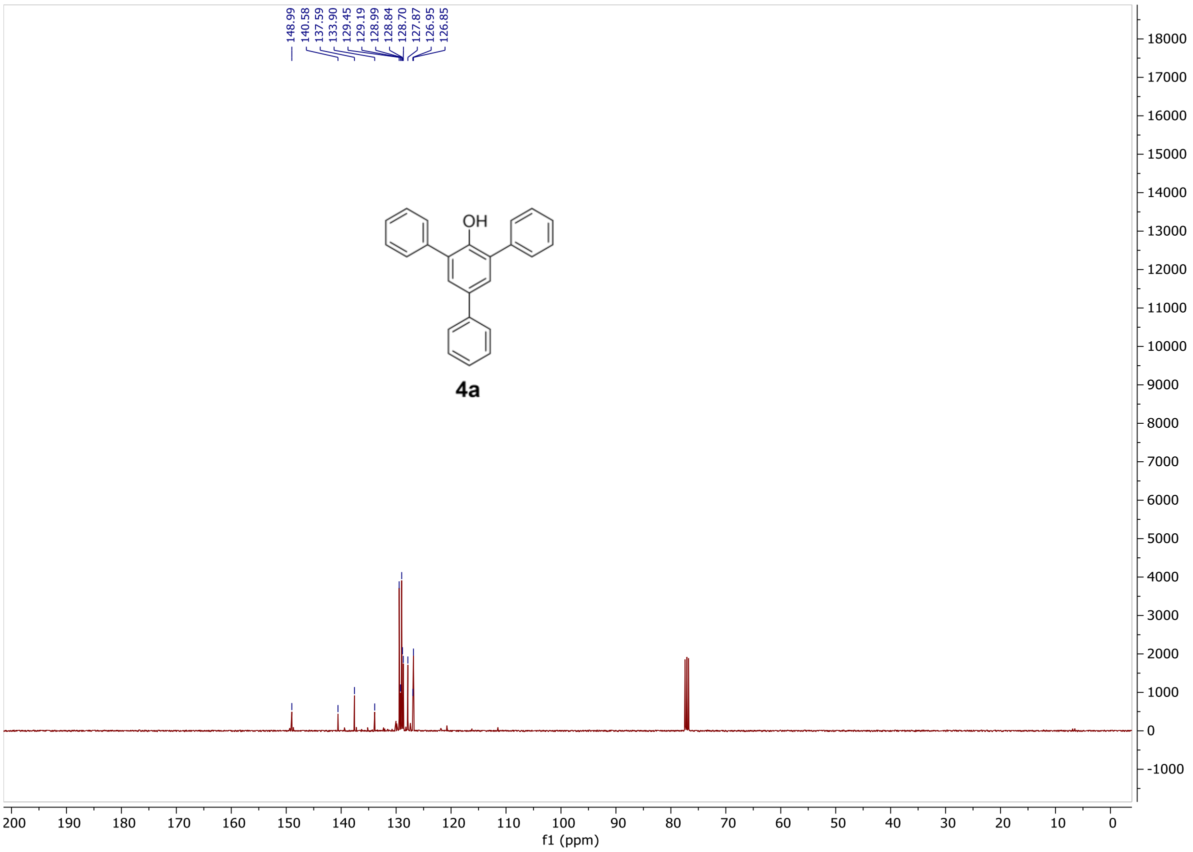

^{1}H-NMR

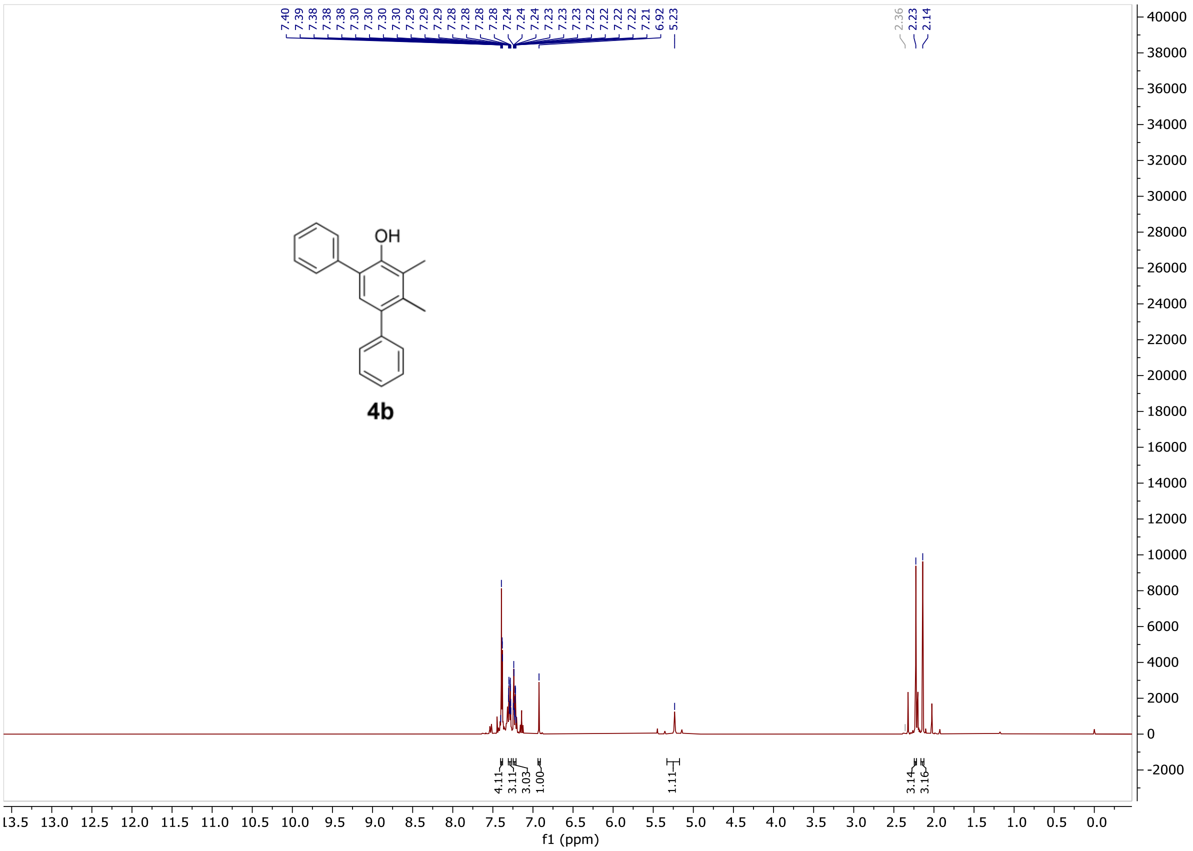

^{13}C-NMR

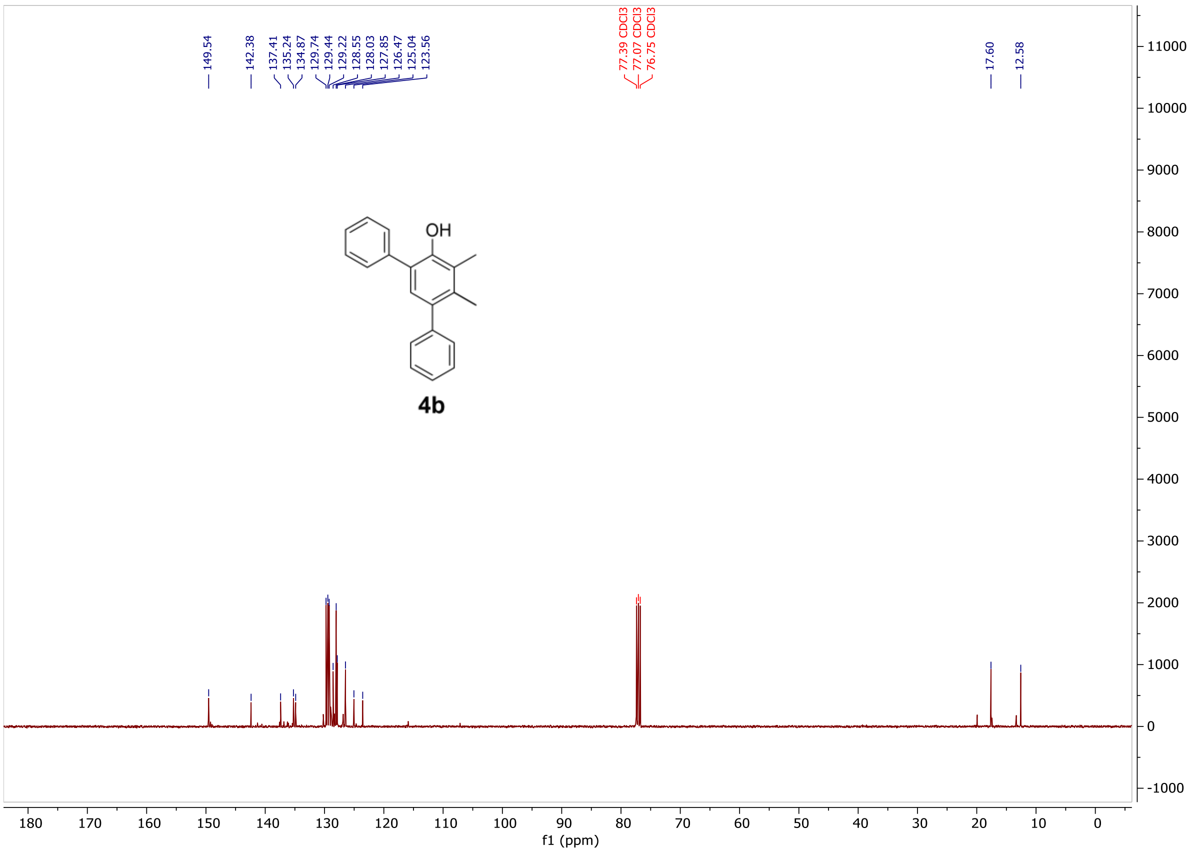

^{1}H-NMR

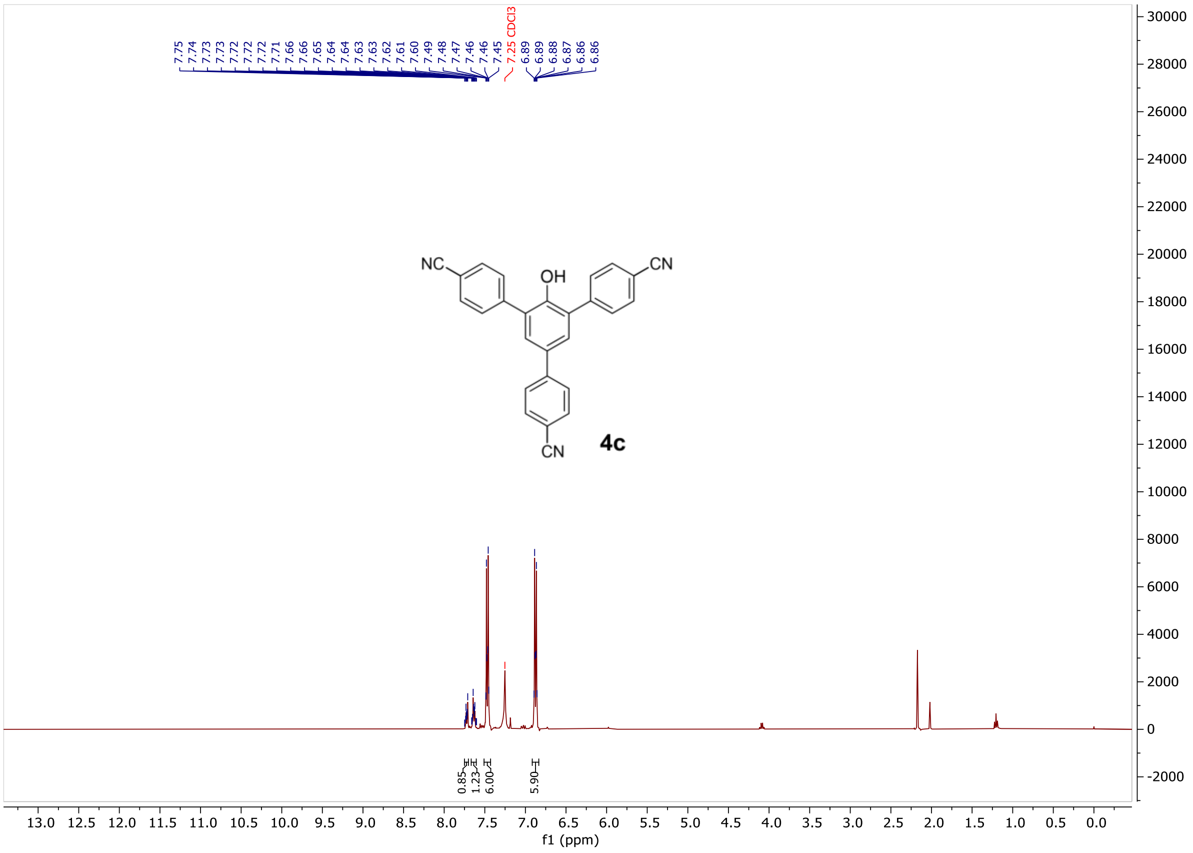

^{13}C-NMR

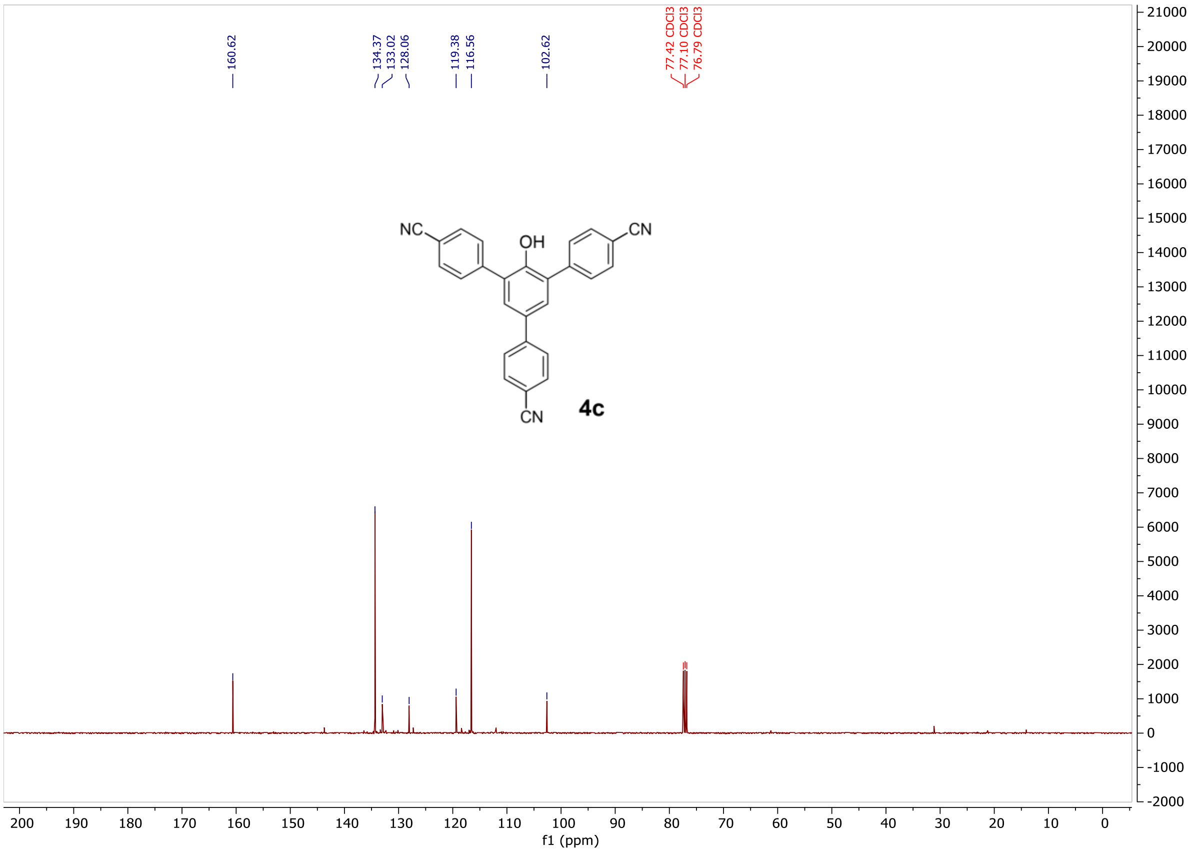

GC-MS (2a) (3 equiv. of H_2O_2)

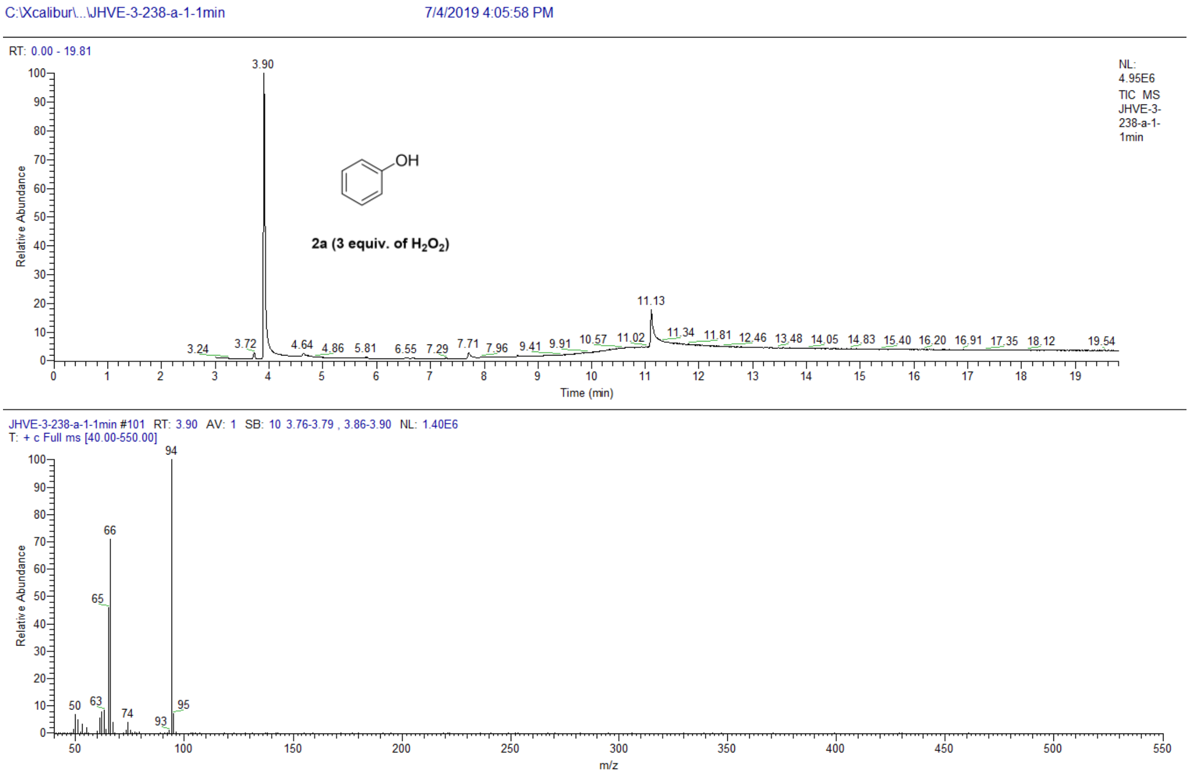

GC-MS (2a) (1.1 equiv. of H_2O_2)

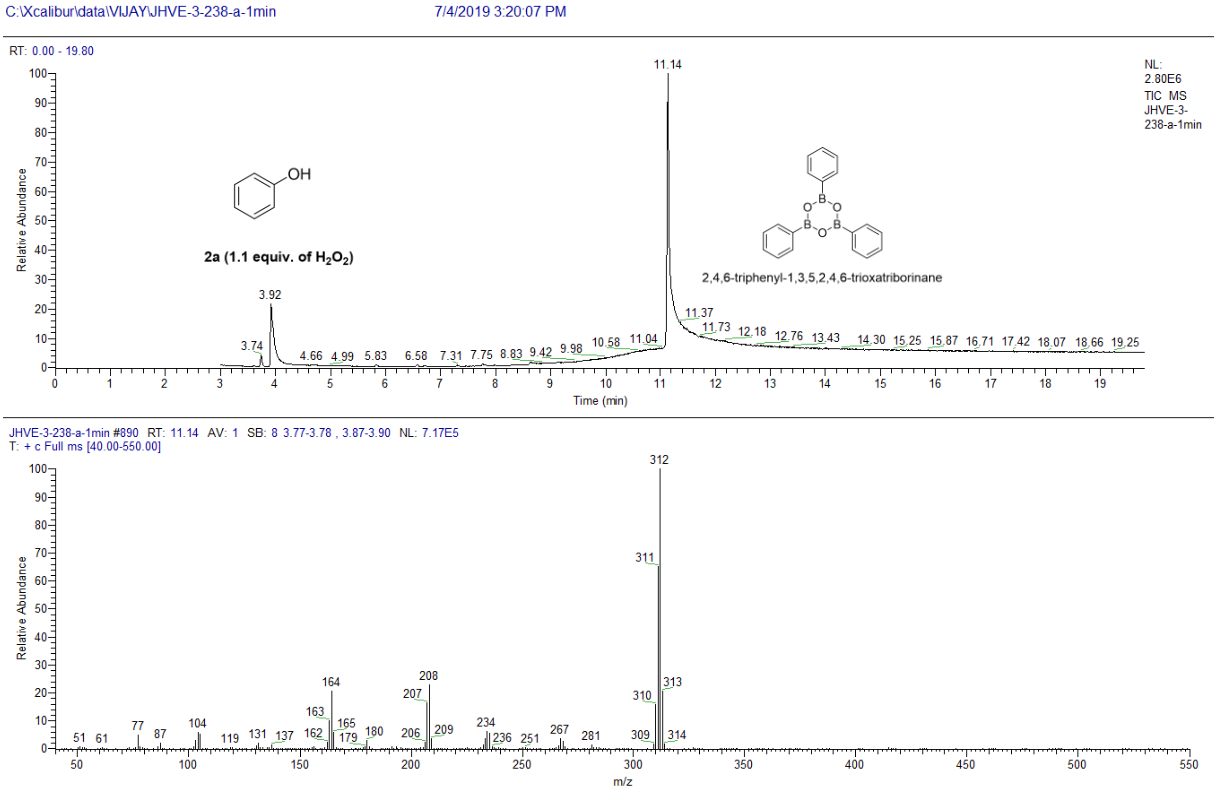

GC-MS (3a) (3 equiv. of H_2O_2 & 3 equiv. of HBr)

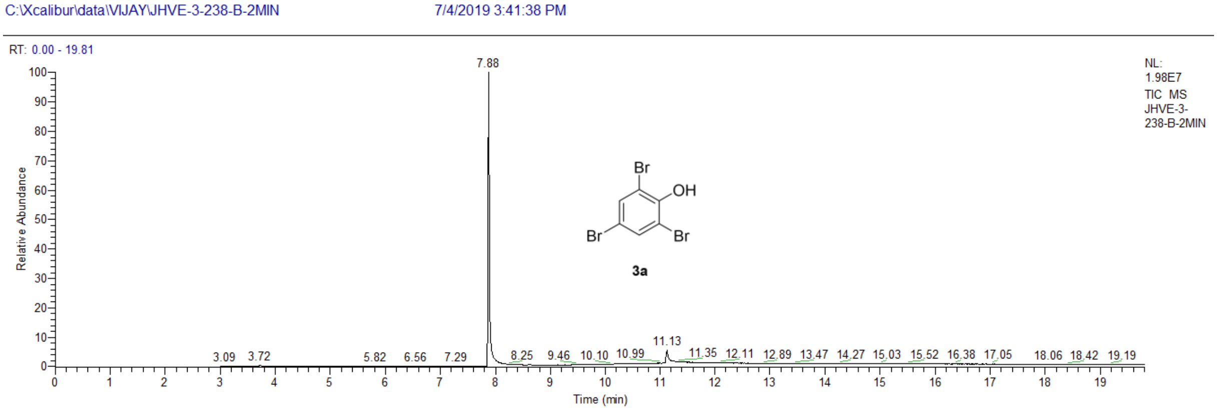

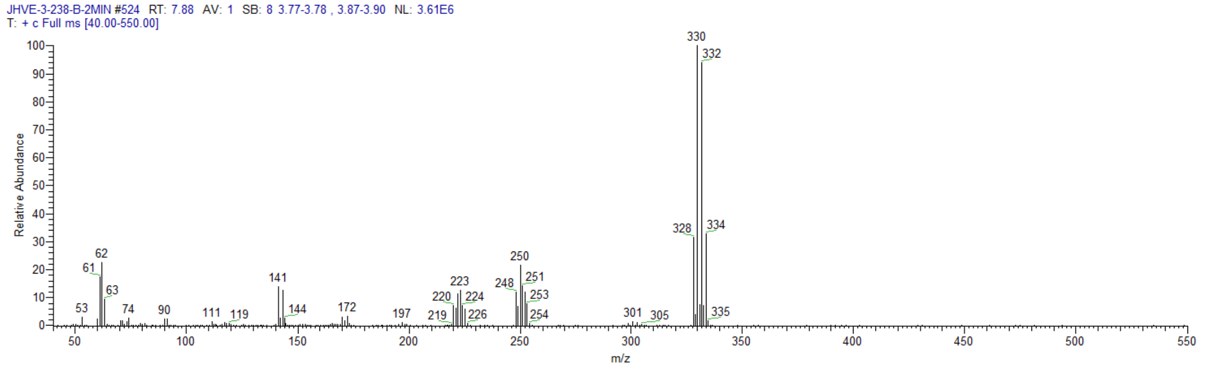

Supplement: RA-010-D0RA08580D-s001 [file RA-010-D0RA08580D-s001.pdf]
